# Endothelial cell‐derived oxysterol ablation attenuates experimental autoimmune encephalomyelitis

**DOI:** 10.15252/embr.202255328

**Published:** 2023-01-30

**Authors:** Florian Ruiz, Benjamin Peter, Jessica Rebeaud, Solenne Vigne, Valentine Bressoud, Martin Roumain, Tania Wyss, Yannick Yersin, Ingrid Wagner, Mario Kreutzfeldt, Marisa Pimentel Mendes, Camille Kowalski, Gael Boivin, Leonard Roth, Markus Schwaninger, Doron Merkler, Giulio G Muccioli, Stephanie Hugues, Tatiana V Petrova, Caroline Pot

**Affiliations:** ^1^ Laboratories of Neuroimmunology, Service of Neurology and Neuroscience Research Center, Department of Clinical Neurosciences Lausanne University Hospital and University of Lausanne Lausanne Switzerland; ^2^ Bioanalysis and Pharmacology of Bioactive Lipids Research Group, Louvain Drug Research Institute UCLouvain, Université Catholique de Louvain Brussels Belgium; ^3^ Department of Oncology University of Lausanne and Ludwig Institute for Cancer Research Lausanne Switzerland; ^4^ SIB Swiss Institute of Bioinformatics Lausanne Switzerland; ^5^ Department of Pathology and Immunology University of Geneva Geneva Switzerland; ^6^ Division of Clinical Pathology, Diagnostic Department University Hospitals of Geneva Geneva Switzerland; ^7^ Department of Pathology and Immunology Geneva Medical School Geneva Switzerland; ^8^ Radio‐Oncology Laboratory, Department of Oncology Lausanne University Hospital and University of Lausanne Lausanne Switzerland; ^9^ Department of Epidemiology and Health Systems, Centre for Primary Care and Public Health (Unisanté) University of Lausanne Lausanne Switzerland; ^10^ Institute for Experimental and Clinical Pharmacology and Toxicology University of Lübeck Luebeck Germany

**Keywords:** cholesterol‐25‐hydroxylase, endothelial cells, experimental autoimmune encephalomyelitis, oxysterols, polymorphonuclear myeloid‐derived suppressor cells, Immunology, Metabolism, Molecular Biology of Disease

## Abstract

The vasculature is a key regulator of leukocyte trafficking into the central nervous system (CNS) during inflammatory diseases including multiple sclerosis (MS). However, the impact of endothelial‐derived factors on CNS immune responses remains unknown. Bioactive lipids, in particular oxysterols downstream of Cholesterol‐25‐hydroxylase (Ch25h), promote neuroinflammation but their functions in the CNS are not well‐understood. Using floxed‐reporter Ch25h knock‐in mice, we trace Ch25h expression to CNS endothelial cells (ECs) and myeloid cells and demonstrate that Ch25h ablation specifically from ECs attenuates experimental autoimmune encephalomyelitis (EAE). Mechanistically, inflamed Ch25h‐deficient CNS ECs display altered lipid metabolism favoring polymorphonuclear myeloid‐derived suppressor cell (PMN‐MDSC) expansion, which suppresses encephalitogenic T lymphocyte proliferation. Additionally, endothelial Ch25h‐deficiency combined with immature neutrophil mobilization into the blood circulation nearly completely protects mice from EAE. Our findings reveal a central role for CNS endothelial Ch25h in promoting neuroinflammation by inhibiting the expansion of immunosuppressive myeloid cell populations.

## Graphical abstract

Cholesterol 25‐hydroxylase (Ch25h) expression increases in central nervous system‐endothelial cells (ECs) during inflammation and regulates lipid secretion. Partial loss of Ch25h in ECs promotes polymorphonuclear myeloid‐derived suppressor cells (PMN‐MDSC) expansion and reduces experimental autoimmune encephalomyelitis (EAE) severity.

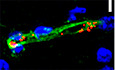

## Introduction

Central nervous system endothelial cells (CNS ECs) are critically involved in multiple sclerosis (MS) pathogenesis through their capacity to regulate leukocyte infiltration within the CNS parenchyma. Moreover, recent evidence suggests that brain microvascular endothelial cell dysfunction is at the forefront of MS pathophysiology (Nishihara *et al*, 2022). However, the underlying molecular mechanisms are incompletely understood.

The development of MS is under the control of both genetic and environmental factors, among which viral infections, in particular exposure to Epstein–Barr virus (EBV) and adolescent obesity (Olsson *et al*, 2017). We and others previously proposed that cholesterol metabolites promote neuroinflammation (Chalmin *et al*, 2015; Durfinova *et al*, 2018). Immunomodulatory cholesterol metabolites include the family of oxidized cholesterol derivatives oxysterols. Cholesterol 25‐hydroxylase (Ch25h) is the rate‐limiting enzyme for the synthesis of 25‐hydroxycholesterol (25‐OHC) and 7α,25‐hydroxycholesterol (7α,25‐diOHC; Mutemberezi *et al*, 2016a), the strongest ligand of G‐protein‐coupled receptor Epstein–Barr virus‐induced gene‐2 (EBI2; Liu *et al*, 2011). Multiple sclerosis and its animal model, the experimental autoimmune encephalomyelitis (EAE), are characterized by inflammatory cell infiltrates and CNS demyelination (Dendrou *et al*, 2015). In this line, we showed that *Ch25h*‐deficient mice display an attenuated EAE disease course compared with wild‐type littermates and that oxysterols downstream Ch25h display pro‐inflammatory properties. Those oxysterols thus favor EAE development (Chalmin *et al*, 2015), and possibly MS by driving pro‐inflammatory lymphocyte trafficking in particular EBI2‐expressing Th17 cells (Chalmin *et al*, 2015; Clottu *et al*, 2017; Wanke *et al*, 2017). By contrast, others showed that Ch25h attenuates interleukine‐1β production from macrophages, dampens sensitivity to septic shock and that *Ch25h* germline knockout mice display an exacerbated EAE compared with Ch25h heterozygote control mice (Reboldi *et al*, 2014). Thus, the role of Ch25h‐pathway during neuroinflammation remains debated.

Since then, independent groups reported that *Ch25h* expression, together with 25‐OHC and 7α,25‐diOHC levels, was increased in the CNS during EAE (Wanke *et al*, 2017; Mutemberezi *et al*, 2018). However, the most critical cellular source of Ch25h‐derived oxysterols during neuroinflammation remains debated, as well as  the function of 25‐OHC, which is a weak agonist of EBI2 and thus unlikely to drive Th17 cell chemotaxis (Liu *et al*, 2011). Ch25h is pleiotropically expressed along the hematopoietic lineage, including macrophages and monocyte‐derived dendritic cells (moDC), known to infiltrate the CNS early during EAE (Chalmin *et al*, 2015). Others have proposed that microglial cells could be the source of Ch25h‐derived oxysterols (Wanke *et al*, 2017). In addition, several studies using different disease mouse models and organs indicate that Ch25h is expressed by nonhematopoietic cells, such as fibroblastic reticular cells, blood endothelial cells (BECs), and lymphatic endothelial cells (LECs; Yi *et al*, 2012; Emgard *et al*, 2018). Recently, the *Ch25h* gene was identified in the blood–brain barrier (BBB) dysfunction module (Munji *et al*, 2019), a subset of 136 genes upregulated in CNS endothelial cells of various mouse disease models associated with BBB dysfunction.

Despite those results, the importance of ECs as a source of Ch25h‐derived oxysterols and the consequences of endothelial Ch25h inactivation during CNS inflammation have not been explored. Additionally, the function of Ch25h during EAE was mostly assessed in the context of immune cell trafficking (Chalmin *et al*, 2015; Wanke *et al*, 2017). In line with this, studies on CNS ECs in neuroinflammation focus on their function in leukocyte diapedesis regulation. Much less is known about the impact of endothelial‐secreted factors, in particular lipids and oxysterols, in the regulation of other aspects of leukocyte activity, such as their expansion or polarization.

Polymorphonuclear myeloid‐derived suppressive cells (PMN‐MDSC) are pathologically activated immunosuppressive neutrophils primarily studied in cancer (Veglia *et al*, 2021) that promote EAE recovery (Knier *et al*, 2018). In cancer, the current  model proposes that their emergence is controlled by two partially overlapping phases: The first step takes place within the bone marrow and spleen and is induced by growth factors derived from tumors, while the second step is favored by pro‐inflammatory signals primarily secreted by the tumor stroma (Condamine *et al*, 2015). However, little is known about the signals driving their expansion during neuroinflammation, and the role of BBB ECs in their generation is virtually unexplored.

In this study, we generated a floxed‐reporter *Ch25h* knock‐in mice and demonstrated that endothelial‐specific Ch25h deletion dampens EAE development. We further showed that *Ch25h* deficiency induces a remodeling of endothelial‐secreted lipids favoring PMN‐MDSC expansion. Accordingly, Ch25h endothelial‐deficient mice display an increased CNS PMN‐MDSC infiltration during EAE. Finally, a combination of Ch25h endothelial deficiency with mature neutrophil depletion resulted in almost complete protection from EAE and favored CNS PMN‐MDSC accumulation. Altogether, our results reveal a novel function of both Ch25h and ECs in the regulation of PMN‐MDSC expansion during neuroinflammation.

## Results

### Ch25h is upregulated in blood endothelial cells during EAE

**Figure 1:**
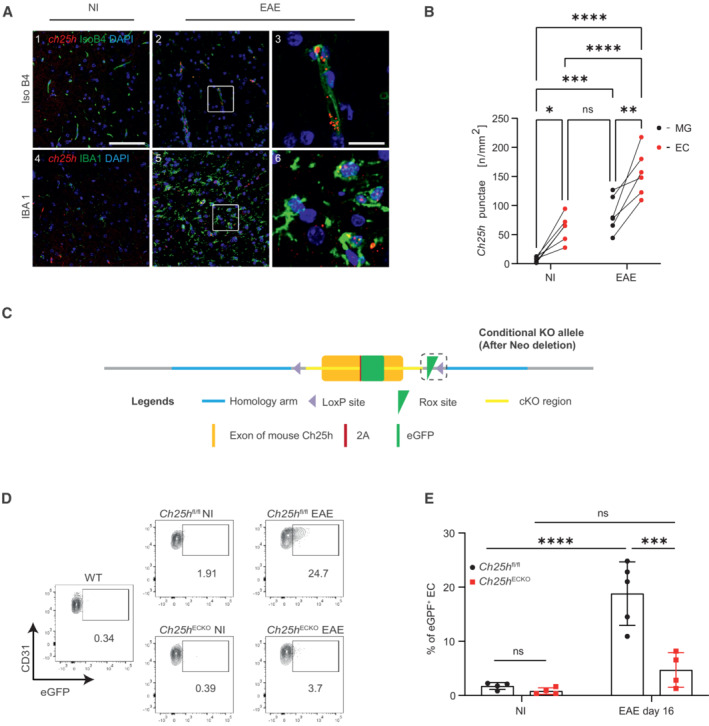
*Ch25h* expression in the central nervous system during EAE RNAscope fluorescence *in‐situ* hybridization of *Ch25h* transcripts (red) in the spinal cord of nonimmunized mice (NI) (left panels) and mice 17 days after EAE immunization (peak disease; two right panels) shown in endothelial cells (Isolectin B4 (IsoB4) green top panels) and activated macrophages/microglia (Ionized calcium biding adaptor molecule 1 (IBA1) green lower panels). Nuclei are shown in blue. Scale bars, 100 μm; insets, 20 μm.Quantitative analysis of Ch25h mRNA expression in the spinal cord of NI and EAE mice comparing expression in macrophages/microglia (MG) and endothelial cells (EC), *n* = 6 biological replicates/group.Construct of *Ch25h‐*eGFP^fl/fl^ mice.Flow cytometry analysis of Ch25h‐eGFP reporter expression in CNS endothelial cells (CNS ECs: Live cells CD45^−^Ter119^−^CD13^−^CD31^+^). Wild‐type mice were used as negative controls for eGFP signal. CNS ECs from Ch25h‐eGFP (*Ch25h* ^fl/fl^) mice and Ch25h‐eGFP^fl/fl^‐Ve‐CadherinCreER^T2^ (*Ch25h* ^ECKO^) mice where the Cre recombinase is expressed in the endothelial cells are compared. Nonimmunized mice (NI) are compared with mice at the peak of EAE 16 days after immunization.Percentage of eGFP^+^ CNS ECs in the same condition as in (D). Symbols indicate individual mice and bars indicate mean ± SD. *Ch25h* ^fl/fl^ NI: *n* = 4 biological replicates, *Ch25h* ^fl/fl^ EAE: *n* = 5 biological replicates, *Ch25h* ^ECKO^ NI: *n* = 4 biological replicates, *Ch25h* ^ECKO^ EAE: *n* = 4 biological replicates. Representative results of one experiment. Data information: ns, nonsignificant, *P ≤ 0.05, **P ≤ 0.005, ***P ≤ 0.0005, ****P ≤ 0.00005. *P*‐values were determined by two‐way ANOVA with Sidak's *post hoc* test.

To identify Ch25h cellular source during EAE, we performed RNA *in‐situ* hybridization of Ch25h in CNS tissue sections, comparing tissues from nonimmunized (NI) mice and mice at the peak of EAE (Score 2.5–3). As Ch25h was shown to be expressed in microglia (Wanke *et al*, 2017) and BECs (Yi *et al*, 2012), Isolectine B4 (IsoB4) and ionized calcium binding adaptor molecule 1 (IBA1) were used to identify ECs and activated macrophages/microglia, respectively. We observed increased Ch25h expression in both ECs (Fig 1A top panels and B) and macrophages/microglia (Fig 1A lower panels and B) during EAE (Fig 1A image 2 low magnification and image 3 high magnification for IsoB4 and image 5 and 6 for IBA1 respectively) compared with nonimmunized (NI) animals (Fig 1A image 1 for IsoB4 and image 4 for IBA1 at low magnification). Interestingly, Ch25h expression was significantly higher in ECs both at steady state and during EAE compared with activated macrophages/microglia (Fig 1B).

To evaluate Ch25h cellular source during neuroinflammation, we generated a floxed‐reporter Ch25h knock‐in mouse, where eGFP is used as a reporter for Ch25h (*Ch25h* ^fl/fl^ mice; Fig 1C). We first characterized eGFP reporter signal in the CNS at baseline and during EAE by flow cytometry. Ch25h‐eGFP expression in CD45^−^Ter119^−^CD13^−^CD31^+^ ECs from the CNS was low in nonimmunized (NI) *Ch25h* ^fl/fl^ mice (Fig 1D, top panel and E, gating strategy shown in Appendix Fig S1A). Strikingly, 16 days after EAE induction, at the peak of disease severity, we noticed a 20‐fold increase in Ch25h‐eGFP signal in CNS ECs (Fig 1D top panel and E) in accordance with our RNA‐scope results (Fig 1B). Ch25h‐eGFP was also detected in CD45^int^CD11b^+^ microglia by flow cytometry (Appendix Fig S1B–D with gating strategy and representative illustration) with a clear trend toward increased expression during EAE (Appendix Fig S1C). We could not compare eGFP expression level in ECs and microglial cells by flow cytometry as different extraction protocols are needed to study the two cell populations.

To further study the role of *Ch25h* in ECs, we crossed *Ch25h* ^fl/fl^ mice with *VE‐cadherin*‐CreER^T2^ mice that express the tamoxifen‐inducible Cre recombinase in endothelial cells (*Ch25h* ^ECKO^; Wang *et al*, 2010). We immunized mice for EAE 2 weeks after tamoxifen‐induced *Ch25h* deletion and validated the robustness of CNS ECs deletion (Fig 1D lower panel and E).

### Deletion of Ch25h in blood endothelial cells dampens EAE

**Figure 2:**
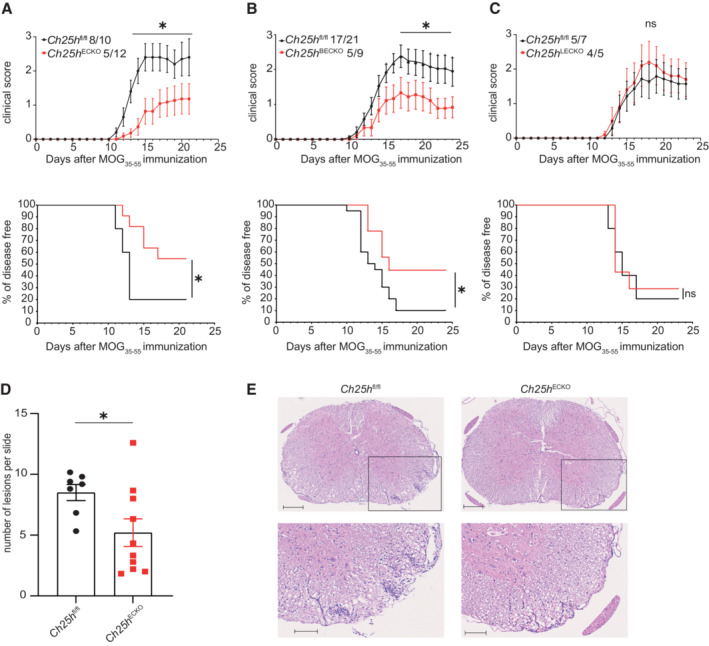
*Ch25h* deletion in blood endothelial cells dampens EAE AEAE disease course in *Ch25h* ^ECKO^ and Cre‐negative littermates (*Ch25h* ^fl/fl^). *Top panel*: EAE clinical score. Bars indicate mean ± SEM. *Bottom panel*: Survival analysis of EAE, depicting disease incidence. Representative results of two experiments with a minimum of *n* = 5 biological replicates/group as indicated on the graph.BAs in (A) except that *Ch25h* ^fl/fl^‐*Pdgfb*‐iCreER^T2^ mice that express Cre recombinase in blood endothelial cells (*Ch25h* ^BECKO^) are shown. Bars indicate mean ± SEM. Representative results of two experiments with a minimum of *n* = 5 biological replicates/group as indicated on the graph.CAs in (A) except that *Ch25h* ^fl/fl^‐*Prox1*‐CreER^T2^ mice that express Cre recombinase in lymphatic endothelial cells (*Ch25h* ^LECKO^) are shown. Bars indicate mean ± SEM. Representative results of two experiments with a minimum of *n* = 5 biological replicates/group as indicated on the graph.D, EHistopathological quantifications (D) and representatives staining (E) of spinal cord sections of immunized *Ch25h* ^fl/fl^ mice (*n* = 7 biological replicates) and *Ch25h* ^ECKO^ mice (*n* = 10 biological replicates) at day 21 post‐immunization for cellular infiltration (HE). Five sections per mouse were quantified. Scale bars 200 μm (top panels), 100 μm (bottom panels). Data information: ns, nonsignificant, *P < 0.05, **P ≤ 0.005, ***P ≤ 0.0005, ****P ≤ 0.00005. *P*‐values were determined by two‐way ANOVA with Sidak's *post hoc* test (top panels A–C) and log‐rank (mantel‐cox) test (bottom panels A–C) or by two‐tailed unpaired *t*‐test (D).

We previously showed that *Ch25h* germline knockout mice develop a less severe disease compared with their wild‐type counterparts (Chalmin *et al*, 2015). Here, using the *Ch25h* ^ECKO^ mice, we found that *Ch25h* deletion in ECs is sufficient to delay the disease onset and to reduce its incidence, while the mean maximal score was similar between the two groups (Fig 2A and Table 1).

**Table 1:** Comparison of EAE disease course in *Ch25h* ^fl/fl^, *Ch25h* ^ECKO^, *Ch25h* ^BECKO^, and *Ch25h* ^LECKO^ mice.

Group	Disease incidence	Mean maximum score (SD)	Mean day of onset (SD)	AUC (SEM)
*Ch25h* ^fl/fl^	80%	3.5 ± 0.93	12.25 ± 0.89	19.55 ± 2.78
*Ch25h* ^ECKO^	45.5%*	2.7 ± 0.67	14.4 ± 1.95 (*P* = 0.05)	7.318 ± 2.55*
*Ch25h* ^fl/fl^	90%	2.94 ± 1.5	14.07 ± 1.91	23.56 ± 3.98
*Ch25h* ^BECKO^	55.5%*	3 ± 0	14.4 ± 1.34	12.29 ± 2.67 (*P* = 0.08)
*Ch25h* ^fl/fl^	71.40%	2.8 ± 0.45	14.4 ± 0.89	14.6 ± 2.7
*Ch25h* ^LECKO^	80%	2.75 ± 0.5	14.5 ± 1.78	17.25 ± 2.73

Related to Fig 1. Ch25h deletion in blood ECs is protective during EAE. AUC, Area under the curve. Mean ± SD or SEM for AUC are indicated. *P*‐values were determined by unpaired student's *t*‐test or log‐rank (mantel‐cox) test for diseases incidence. *P < 0.05. Representative results of two independent experiments per genotype with a minimum of *n* = 5 biological replicates/group.

*VE‐cadherin*‐CreER^T2^ mice express the Cre recombinase in both lymphatic endothelial cells (LECs) and BECs and both cell types have been shown to express *Ch25h* (Yi *et al*, 2012; Emgard *et al*, 2018). To distinguish the role of Ch25h in these endothelial subtypes, we generated mouse strains with specific deletion of *Ch25h* in BECs and LECs by crossing *Ch25h* ^fl/fl^ mice with *Pdgfb*‐iCreER^T2^ (Claxton *et al*, 2008) and *Prox1*‐CreER^T2^ (Bernier‐Latmani *et al*, 2015) lines, respectively. We then compared the EAE phenotype of *Ch25h* ^fl/fl^; *Pdgfb*‐iCreER^T2+^ mice (*Ch25h* ^BECKO^), *Ch25h* ^fl/fl^; *Prox1*‐CreER^T2+^ mice (*Ch25h* ^LECKO^); and Cre‐negative control littermates injected with tamoxifen. *Ch25h* ^BECKO^ mice displayed partial protection, similar to *Ch25h* ^ECKO^ mice (Fig 2B and Table 1), whereas inactivation of *Ch25h* in LECs had no effect on EAE phenotype (Fig 2C and Table 1). We further evaluated CNS infiltrates in EAE‐diseased animals in *Ch25h* ^ECKO^ mice compared with *Ch25h* ^fl/fl^ mice. Significant differences were observed with decreased numbers of inflammatory lesions in *Ch25h* ^ECKO^ compared with *Ch25h* ^fl/fl^ mice assessed by histology (Fig 2D and E). Altogether, our results show that Ch25h expressed by BECs plays a central role in promoting EAE.

### Ch25h deletion in CNS ECs upregulates genes related to polyunsaturated fatty acid biosynthesis and metabolism

**Figure 3:**
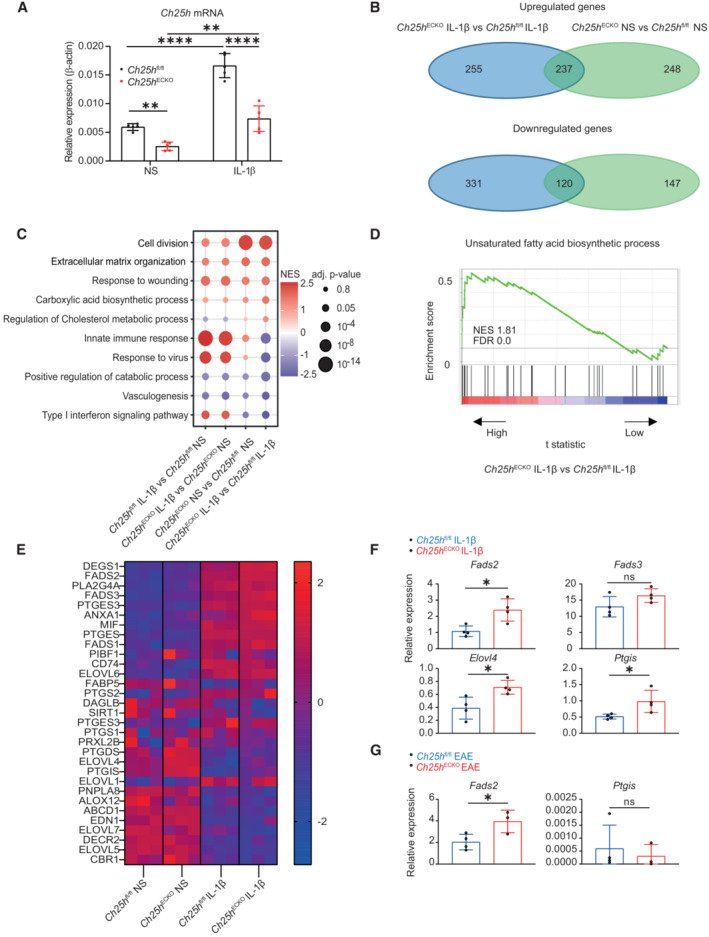
Transcriptomic alterations in *Ch25h* ^ECKO^ CNS endothelial cells RT‐qPCR analysis of Ch25h expression in primary mouse brain microvascular endothelial cells (pMBMECs) isolated from Ch25h^fl/fl^ and *Ch25h* ^ECKO^ mice. Cells were left unstimulated (NS) or stimulated with IL‐1β. *n* = 5 biological replicates/group. The experiment was performed three times.Venn diagram of differentially expressed genes (DEG) between *Ch25h* ^ECKO^ and *Ch25h* ^fl/fl^ pMBMECs assessed by RNA sequencing comparing nonstimulated (NS) and IL‐1β stimulated cells. *n* = 3 biological replicates/group. The experiment was realized once.Dot plot of gene set enrichment analysis (GSEA) showing selected pathways. NES, normalized enrichment score.GSEA comparing enrichment of genes related to unsaturated fatty acid biosynthesis in *Ch25h* ^ECKO^ vs. *Ch25h* ^fl/fl^ IL‐1β stimulated pMBMECs. NES, normalized enrichment score; FDR, false discovery rate *q*‐value.Heatmap showing normalized expression (*z*‐scores) of gene counts from the RNA‐seq analysis related to the unsaturated fatty‐acid biosynthetic pathway.RT‐qPCR of pMBMECs isolated from *Ch25h* ^ECKO^ and *Ch25h* ^fl/fl^ mice and stimulated with IL‐1β. *n* = 4 biological replicates/group. The experiment was realized once.RT‐qPCR of CNS endothelial cells sorted from *Ch25h* ^ECKO^ and *Ch25h* ^fl/fl^ mice during EAE (Day 10 after immunization). *Ch25h* ^ECKO^ *n* = 3 biological replicates, *Ch25h* ^fl/fl^ *n* = 4 biological replicates. The experiment was performed once. Data information: Bars indicate mean ± SD. ns, nonsignificant, *P ≤ 0.05, **P ≤ 0.005, ****P ≤ 0.00005. *P*‐values were determined by two‐way ANOVA with Sidak's *post hoc* test (A) or by two‐tailed unpaired *t*‐test (F, G).

To gain mechanistic insights into the function of endothelial Ch25h in inflammation, we isolated and cultured primary mouse brain microvascular endothelial cells (pMBMECs) from tamoxifen‐injected *Ch25h* ^ECKO^ and *Ch25h* ^fl/fl^ control mice. Confluent pMBMECs were stimulated or not with the pro‐inflammatory cytokine IL‐1β since IL‐1 signaling in ECs of the BBB plays a crucial role in driving EAE (Hauptmann *et al*, 2020). *Ch25h* mRNA expression was significantly upregulated by IL‐1β stimulation (Fig 3A). Additionally, *Ch25h* transcripts were reduced in brain ECs isolated from *Ch25h* ^ECKO^ mice as compared with *Ch25h* ^fl/fl^ ECs in accordance with the results obtained *in vivo* during EAE. We next analyzed the transcriptome of pMBMECs at baseline conditions or upon IL‐1β stimulation by RNA sequencing (RNA‐seq). Using a false discovery rate (FDR) < 0.05 as cutoff and comparing *Ch25h* ^ECKO^ pMBMECs to *Ch25h* ^fl/fl^ ECs either at baseline or upon IL‐1β stimulation, we identified 1,338 differentially expressed genes, 740 of which were upregulated in *Ch25h* ^ECKO^ pMBMECs, while 598 were downregulated. More than 20% of these genes were altered both at baseline and upon IL‐1β stimulation (Fig 3B and Datasets [Link], [Link]). To identify the pathways altered in the absence of Ch25h, we performed a gene set enrichment analysis (GSEA). One of the most striking findings was that *Ch25h* deletion enhanced cell division‐related gene expression independently from IL‐1β stimulation (Fig 3C). This is consistent with recent findings showing that Ch25h has angiostatic effects (Lu *et al*, 2021b). Interestingly, IL‐1β stimulation enhanced the expression of genes related to extracellular matrix organization and response to wounding, which were further increased in *Ch25h* ^ECKO^ compared with *Ch25h* ^fl/fl^ pMBMECs (Fig 3C). We also identified enrichment in genes related to carboxylic acid biosynthetic process, which was only significantly increased when IL‐1β‐stimulated *Ch25h* ^ECKO^ pMBMECs were compared with *Ch25h* ^fl/fl^ pMBMECs, suggesting that this pathway is regulated by Ch25h specifically under inflammatory conditions (Fig 3C). Ch25h and 25‐OHC can regulate cholesterol metabolism (Adams *et al*, 2004). However, GSEA indicated that alteration of cholesterol metabolism was not at the forefront of transcriptomic changes observed upon *Ch25h* deletion (Fig 3C). Downregulated pathways in *Ch25h* ^ECKO^ pMBMECs compared with control cells included, among others, innate immune response, response to virus, positive regulation of the catabolic process, vasculogenesis, and type I interferon signaling pathway (Fig 3C). As the carboxylic acid biosynthesis pathway was only enriched in IL‐1β‐stimulated *Ch25h* ^ECKO^ compared with *Ch25h* ^fl/fl^ pMBMECs, we focused our attention on genes from this gene set. Carboxylic acids include unsaturated fatty acids, which are regulators of the immune response. We thus specifically tested the enrichment of genes associated with unsaturated fatty acid biosynthesis and found that they were increased in IL‐1β‐stimulated *Ch25h* ^ECKO^ compared with *Ch25h* ^fl/fl^ pMBMECs (Fig 3D). Intriguingly, this latter gene set included prostaglandin I_2_ synthase (PTGIS), an enzyme that catalyzes the biosynthesis and metabolism of eicosanoids, in particular the isomerization of prostaglandin H_2_ to prostaglandin I_2_ (PGI_2_). Prostaglandin I_2_ can be secreted by vascular endothelial cells and has been shown to promote neuronal remodeling in a localized model of EAE (Muramatsu *et al*, 2012). We also observed an increase in fatty acid desaturase 2 (FADS2) expression, which promotes the production of anti‐inflammatory lipids (Liu *et al*, 2020). The elongase ELOVL4 and fatty acid desaturase 3 (FADS3) were also upregulated in *Ch25h* ^ECKO^ pMBMECs. We further generated a heatmap of genes implicated in the biosynthesis of unsaturated fatty acids and observed that among the abovementioned genes, FADS2 and FADS3 were upregulated by IL‐1β and increased in absence of Ch25h (Fig 3E and Datasets [Link], [Link]). Next, we confirmed by RT‐qPCR that FADS2, PTGIS, and ELOVL4 were significantly upregulated in *Ch25h* ^ECKO^ versus *Ch25h* ^fl/fl^ pMBMECs under IL‐1β stimulation (Fig 3F). We then compared our RNA‐seq to genes differentially expressed in CNS ECs in response to EAE (Munji *et al*, 2019), searching for matching genes after Ch25h deletion, IL‐1β stimulation or during EAE. We found that FADS2 and PTGIS were upregulated in CNS ECs during EAE (Munji *et al*, 2019). To confirm these results, we FACS‐sorted CNS ECs from *Ch25h* ^ECKO^ and *Ch25h* ^fl/fl^ EAE mice and evaluated mRNA levels of FADS2 and PTGIS. We confirmed *ex vivo* that FADS2 mRNA transcripts were significantly higher in CNS ECs of *Ch25h* ^ECKO^ mice (Fig 3G). Overall, during inflammation, loss of *Ch25h* in ECs alters lipid biosynthetic pathways, among which the upregulation of FADS2 was confirmed both *in vitro* and *in vivo* in the CNS during EAE.

### *Ch25h* deletion in pMBMECS during inflammation alters lipid secretion

**Figure 4:**
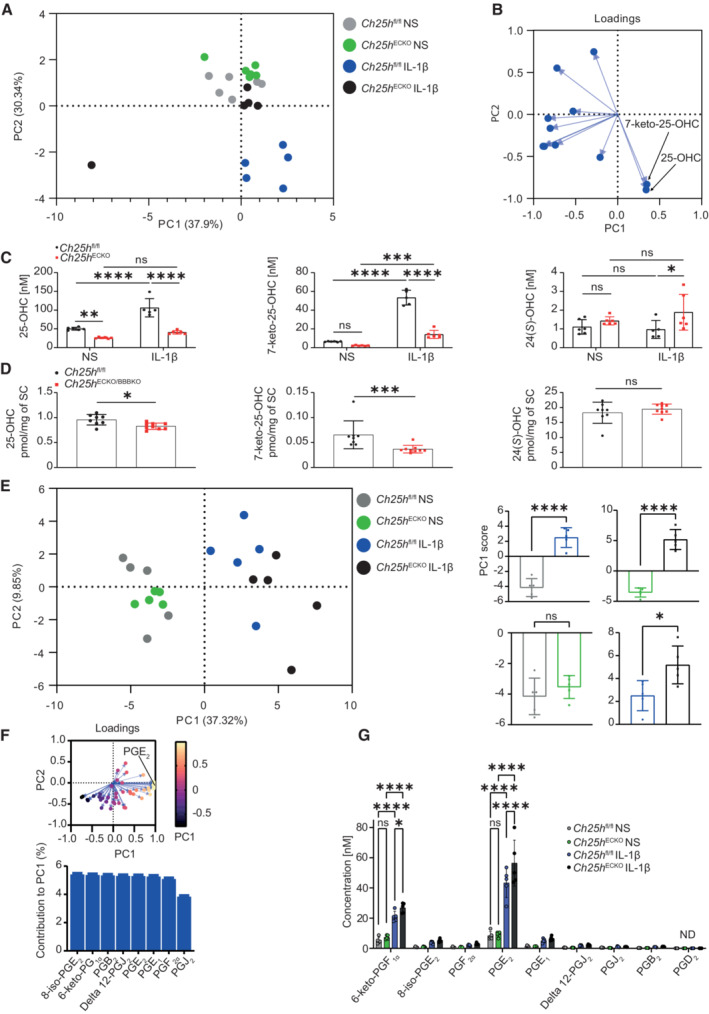
Ch25h deletion in CNS endothelial cells induces a remodeling of secreted lipids Principal component analysis of 10 oxysterols measured by HPLC‐MS in the supernatant of pMBMECs isolated from *Ch25h* ^fl/fl^ mice and *Ch25h* ^ECKO^ mice. Cells were left unstimulated (NS) or stimulated by IL‐1β.Loading plot showing the contribution of oxysterols to PC1 and PC2.25‐OHC, 7‐keto‐25‐OHC, and 24*(S)*‐OHC concentration in the same conditions as in (B). Bars represent mean ± SD. *n* = 6 biological replicates/group except for *Ch25* ^fl/fl^ IL‐1β were *n* = 5 biological replicates. The experiment was performed once.25‐OHC, 7‐keto‐25‐OHC, and 24*(S)*‐OHC levels in spinal cord tissue extracted from *Ch25h* ^fl/fl^ (*n* = 8), *Ch25h* ^ECKO^ (*n* = 3) and *Ch25h* ^BBBKO^ (*n* = 6) at the peak of EAE. Bars indicate mean ± SD.
*Left panel*: Principal component analysis of 49 eicosanoids measured by HPLC‐MS in the supernatant of pMBMECs in the same conditions as in (A). *n* = 5 biological replicates/group. *Right panel*: Comparison of PC1 scores between the different conditions. Bars indicate mean ± SD.
*Upper panel*: loading plot showing the contribution of each detected eicosanoid to PC1 and PC2. *Lower panel*: Relative contribution of prostaglandins to PC1.Prostaglandin concentration (nM) in the supernatant of pMBMECs comparing conditions mentioned in (A). *n* = 5 biological replicates/group and bars represent mean ± SD. Data information: ns, nonsignificant, *P < 0.05, ***P ≤ 0.0005, ****P ≤ 0.00005. *P*‐values were determined by two‐tailed unpaired *t*‐test (D, E) or by two‐way ANOVA with Sidak's *post hoc* test (C, G).

**Figure EV1:**
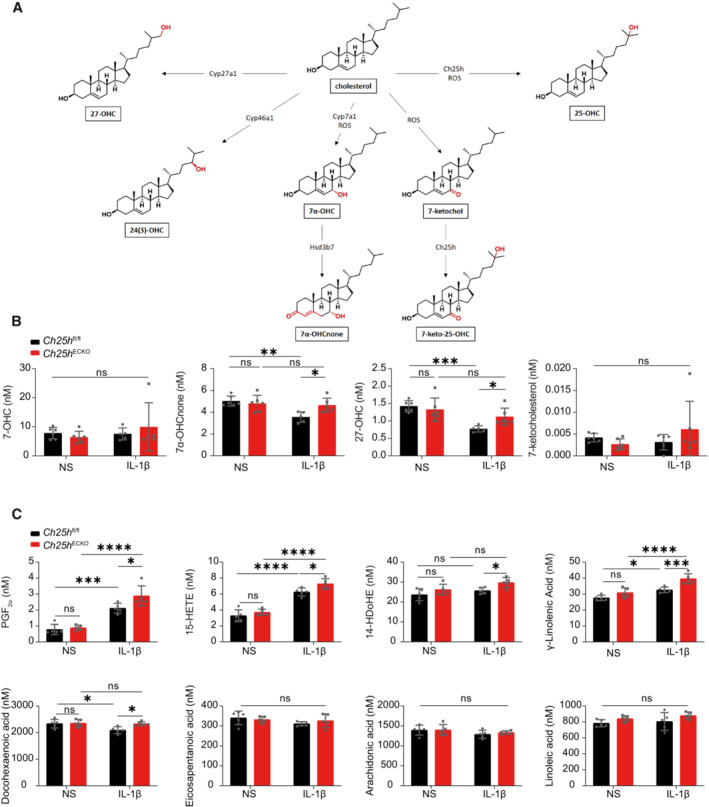
Related to Fig 4 Oxysterol and eicosanoids levels Schematic representation of oxysterol metabolic pathways. Dashed arrow indicates a proposed pathway (as in Myers *et al*, 2013).Primary mouse brain microvascular endothelial cells (pMBMEC) were isolated from *Ch25h* ^fl/fl^ and *Ch25h* ^fl/fl^‐Ve‐CadherinCreERT2 mice (*Ch25h* ^ECKO^) injected with tamoxifen. Cells were left unstimulated (NS) or stimulated with IL‐1β (10 ng/ml) during 24 h. Supernatant was then collected. Oxysterols were measured by HPLC‐MS. 7‐hydroxycholesterol (7‐OHC), 7α‐hydroxycholestenone (7α‐OHCnone), 27‐hydroxycholesterol (27‐OHC), 7‐ketocholesterol concentrations. *n* = 6 biological replicates/group except for *Ch25h* ^fl/fl^ IL‐1β *n* = 5. Bars indicate mean ± SD.Same conditions as in (B), except that eicosanoids were measured by Liquid Chromatography‐Mass Spectrometry. Prostaglandin F_2α_ (PGF_2α_), 15‐Hydroxyeicosatetraenoic acid (15‐HETE), 14‐hydroxy‐4Z,7Z,10Z,12 E,16Z,19Z‐docosahexaenoic acid (14‐HDoHE), γ‐linolenic acid, Docohexanoic acid, Eicosapentanoic acid, Arachidonic acid, Linoleic acid concentrations. Bars indicates mean ± SD. *n* = 5 biological replicates/group. Data information: ns, nonsignificant, *P < 0.05, **P ≤ 0.005, ***P ≤ 0.0005, ****P ≤ 0.00005. *P*‐values were determined by two‐way ANOVA with Sidak's *post hoc* test.

Ch25h is the rate‐limiting enzyme for the synthesis of 25‐OHC and is also implicated in the production of 7α,25‐diOHC and 7‐keto‐25‐OHC (Beck *et al*, 2019). Moreover, FADS2 is a key enzyme for the synthesis of a vast number of eicosanoids (Liu *et al*, 2020). We thus reasoned that Ch25h deletion in ECs could have a broad impact on endothelial‐secreted lipids. We evaluated lipid production by CNS ECs by first measuring 10 oxysterols (schematic representation of cholesterol metabolism, Fig EV1A) in the supernatant of control *Ch25h* ^fl/fl^ or *Ch25h* ^ECKO^ pMBMECs at baseline and upon IL‐1β stimulation. Using principal component analysis (PCA), we found that IL‐1β stimulation altered oxysterol production in *Ch25h* ^fl/fl^ but less in *Ch25h* ^ECKO^ pMBMECs (Fig 4A). To identify which oxysterols were responsible for the observed differences, we generated a loading plot to visualize the relative contributions of each oxysterol to PC1 and PC2 (Fig 4B). With this approach, we found that 25‐OHC and 7‐keto‐25‐OHC were the only two oxysterols that were increased (Fig 4B). In addition, 25‐OHC and 7‐keto‐25‐OHC concentrations were higher than the other oxysterols measured under IL‐1β stimulation (Figs 4C and EV1B, and Appendix Fig S2A for representative chromatograms). Further analysis revealed that IL‐1β stimulation increased the production of 25‐OHC and 7‐keto‐25OHC in *Ch25h* ^fl/fl^ pMBMECs, while their production was significantly reduced in *Ch25h* ^ECKO^ cells compared with *Ch25h* ^fl/fl^ cells (Fig 4C). Evaluating oxysterol production in *Ch25h* ^fl/fl^ and *Ch25h* ^ECKO^ spinal cords obtained from EAE mice, we found that 25‐OHC and 7‐keto‐25‐OHC were decreased in *Ch25h* ^ECKO^ mice compared to *Ch25h* ^fl/fl^ mice (Fig 4D). However, the magnitude of the decrease of 25‐OHC and 7‐keto‐25‐OHC in *Ch25h* ^ECKO^ mice was lower than what we found *in vitro* in pMBMECs. This is in accordance with our observation that other cells, in particular microglia, express Ch25h, which expression is not affected in *Ch25h* ^ECKO^mice.

In pMBMECs supernatant, 24*(S)*‐OHC levels were 10 times lower than 25‐OHC (Fig 4C) and 7‐keto‐25‐OHC. 25‐OHC was not increased in absence of Ch25h in the IL‐1β stimulated condition while 7‐keto‐25‐OHC was significantly increased by 1.5‐fold in absence of Ch25h in the IL‐1β stimulated condition (Fig 4C). We did not detect Cyp46a1, the main enzyme‐producing 24*(S)*‐OHC in our pMBMECs on the RNA‐seq data, it is thus not excluded that the 24(*S*)‐OHC detected comes from the fetal bovine serum used in pMBMECs culture media. 24(*S*)‐OHC is metabolized principally by neurons and was in contrast to pMBMECs detected at high levels *ex vivo* in the spinal cord of EAE mice (Fig 4D). Moreover, in mouse spinal cords, 24(*S*)‐OHC levels were 10 times higher than the levels of 25‐OHC and 7‐keto‐25‐OHC (Fig 4D). Those results suggest that 25‐OHC is the major oxysterol produced by microvascular CNS endothelial cells and the concentrations of this oxysterol at the level of the microvessels might be substantially higher than its systemic levels. Indeed, the other oxysterols measured *in vitro* in the pMBMEC supernatants were detected at lower levels than 25‐OHC. 27‐OHC (Fig EV1B) and 7α‐hydroxycholestenone (7α‐OHCnone; Fig EV1B) were reduced under inflammatory conditions and Ch25h deletion increased 27‐OHC production by 1.5‐fold while 7‐ketocholesterol and 7‐OHC levels remained unchanged (Fig EV1B). Oxysterols can be generated by enzymatic pathways and by ROS‐mediated cholesterol oxidation (so‐called auto‐oxidation). 7‐ketocholesterol is a marker of cholesterol auto‐oxidation levels and as its levels remained unchanged (Fig EV1B), this indicates that Ch25h enzymatic activity is likely responsible for the observed changes.

To extend our lipidomic analysis to FADS2 downstream metabolites, we measured a panel of 100 eicosanoids in the supernatant of IL‐1β treated or control pMBMECs (see Materials and Methods for the full list). We retained 49 eicosanoids for further analysis as they were above the detection threshold and detected in all the samples. Principal component analysis was performed to evaluate differences in eicosanoid production among conditions (Fig 4E, left panel). PC1 alone explained 38% of the total variance of eicosanoids and was retained to compare experimental groups. Comparison of PC1 scores for each sample in the different conditions revealed that IL‐1β strongly altered eicosanoids production in pMBMECs (Fig 4E left and right panel) and that Ch25h deletion altered the secretion of several lipids only in pMBMECs stimulated with IL‐1β (Fig 4E right panel). Using the same approach as for oxysterols, we identified a group of eicosanoids contributing to PC1 (Fig 4F, top panel). Most of these variables belong to the prostaglandin family and the sum of their relative contribution to PC1 reached 40% (Fig 4F, bottom panel). Specifically, prostaglandin E_2_ (PGE_2_) was a strong contributor (Fig 4F, top panel). Comparison of the concentration of prostaglandins in pMBMECs supernatants revealed that IL‐1β increased the secretion of PGE_2_ and the stable metabolite of PGI_2_ (6‐keto‐PGF_1α_; Fig 4G and Dataset EV5 for the list of internal standards used and Appendix Fig S2B for representative chromatograms). These were the two prostaglandins detected at the highest level in IL‐1β‐stimulated pMBMECs supernatant (Fig 4G). Most importantly, Ch25h deletion significantly increased the secretion of these two metabolites (Fig 4G).

FADS2 is the rate‐limiting enzyme for the desaturation of linoleic acid into γ‐linoleic acid which itself is a precursor of prostaglandins (Nakamura & Nara, 2004). In accordance with this, γ‐linolenic acid levels were significantly increased by IL‐1β stimulation and further enhanced by *Ch25h* deletion (Fig EV1C). We observed a similar pattern in prostaglandin F_2α_ (PGF_2α_) and 15‐hydroxyeicosatetranoic acid (15‐HETE; Fig EV1C). 14‐hydroxydocosahexaenoic acid (14‐HDoHE) was only increased in *Ch25h*‐deficient endothelial cells stimulated by IL‐1β (Fig EV1C) while docosahexaenoic acid levels were reduced by IL‐1β only in *Ch25h* ^fl/fl^ samples but maintained in the absence of *Ch25h* (Fig EV1C). Other eicosanoids such as eicosapentaenoic acid, arachidonic acid, or linoleic acid were not affected by Ch25h deletion nor IL‐1β stimulation (Fig EV1C). Prostaglandin‐endoperoxide synthase 2 (PTGS2 or COX2) or prostaglandin E synthase expressions were not affected by Ch25h deletion (Dataset EV3) in inflammatory conditions. In conclusion, Ch25h inactivation and IL‐1β stimulation in pMBMECs alter oxysterols and eicosanoids secretion. Specifically, eicosanoid and prostaglandin precursor γ‐linolenic acid produced by FADS2 and multiple eicosanoids were increased in *Ch25h*‐deficient endothelial cells under inflammatory conditions. Thus, we propose that FADS2 upregulation induced by *Ch25h* deletion could be at the origin of the differences in eicosanoid levels described above and that Ch25h is an upstream regulator of this enzyme.

### Increased infiltration of PMN‐MDSC in the CNS of *Ch25h* ^ECKO^ mice during EAE

**Figure 5:**
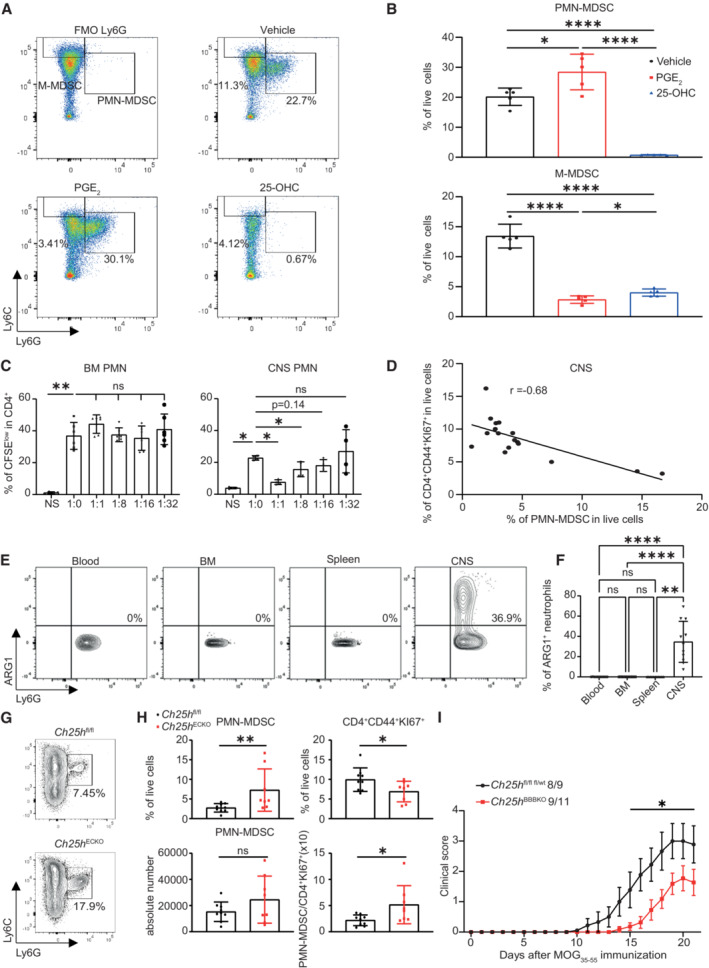
Ch25h deletion in endothelial cells favors PMN‐MDSC expansion in the CNS during EAE Flow cytometry analysis of bone marrow‐derived cells cultured in MDSC polarizing conditions treated with vehicle control (EtOH), PGE2 (20 nM) and 25‐OHC (1 μM). M‐MDSC = Live cells CD11b^+^ Ly6C^high^ Ly6G^−^, PMN‐MDSC = Live cells CD11b^+^ Ly6C^int^ Ly6G^+^.Same conditions as in (A) except that bar graphs are shown. Symbols depict mean percentage of PMN‐MDSC (upper panel) and M‐MDSC (lower panel) in live cells. *n* = 5 biological replicates/group. Representative results of two independent experiments. Bars indicate mean ± SD.Impact of CNS PMN‐MDSC and BM‐PMN on CD4^+^ T cell proliferation assessed by CFSE dilution using flow cytometry. PMN‐MDSC were FACS‐sorted from the CNS (right panel) and BM (left panel) of WT mice at the peak of EAE. NS, nonstimulated. *n* = 4 biological replicates/group. Bars indicate mean ± SD. CNS PMN‐MDSC/CD4^+^ T cells: representative results of two independent experiments; BM‐PMN: 1 experiment.Correlation of the percentage of PMN‐MDSC in live cells with the percentage CD4^+^CD44^+^Ki67^+^ cells in live cells in the CNS at the peak of EAE assessed by flow cytometry. *n* = 17 biological replicates.Flow cytometry analysis of PMN‐MDSC in the blood, BM, spleen and CNS for Arginase‐1 staining.Quantification of (E) *n* = 12 biological replicates. The experiment was perfomed once. Bars indicate mean ± SD.Flow cytometry analysis of PMN‐MDSC in the CNS at the peak of EAE in *Ch25h* ^fl/fl^ and *Ch25h* ^ECKO^ mice.Percentage (upper left panel) and absolute number (lower left panel) of CNS live cells CD45^+^CD11b^+^Ly6C^int^Ly6G^+^ PMN‐MDSC; Percentage of live CD4^+^CD44^+^Ki67^+^ cells (upper right panel) and ratio (lower right panel) of absolute number of PMN‐MDSC and proliferating CD4^+^ T cells in the CNS at the peak of the disease of *Ch25h* ^fl/fl^ (*n* = 9) and *Ch25h* ^ECKO^ mice (*n* = 8). Symbols depict individual mice and bars indicate mean ± SD. Combined results of two independent experiments.EAE disease course in *Ch25h* ^BBBKO^ mice (*n* = 11) where the Cre‐recombinase is expressed in endothelial cells of the CNS and Cre‐negative littermates (*Ch25h* ^fl/fl^: *n* = 7 and *Ch25h* ^fl/wt^: *n* = 2). Bars indicate mean ± SEM. Representative results of two independent experiments. Data information: ns, nonsignificant, *P < 0.05, **P ≤ 0.005, ****P ≤ 0.00005. *P*‐values were determined by two‐tailed unpaired *t*‐test (B, C, H), Spearman correlation (D) and by two‐way ANOVA with Sidak's *post hoc* test (F, I).

We observed that PGE_2_ production in response to IL‐1β was potentiated in the absence of *Ch25h* in cultured ECs. Mechanistically, PGE_2_ signaling through EP4 expands MDSC (Lu *et al*, 2021a), and PMN‐MDSC are protective during EAE (Knier *et al*, 2018). We thus hypothesized that pMBMEC‐secreted lipids induced by IL‐1β, in particular PGE_2_ and 25‐OHC, affect MDSC expansion. Using bone marrow‐derived cells (BMDCs) cultivated under MDSC polarizing conditions (Marigo *et al*, 2010), we observed that the addition of PGE_2_ increased the population of CD11b^+^Ly6C^int^Ly6G^+^ cells, representing PMN‐MDSC (Fig 5A and B upper panel) but decreased the population of CD11b^+^Ly6C^high^Ly6G^−^ cells, representing monocytic‐MDSC (M‐MDSC; Fig 5A and B, lower panel) after 4 days of culture. The addition of 25‐OHC reduced the percentage of M‐MDSC to a similar extent as PGE_2_ (Fig 5A and B). Strikingly, 25‐OHC almost completely abrogated PMN‐MDSC expansion *in vitro* (Fig 5A and B). Hence, we propose that (i) PGE_2_ and 25‐OHC exert opposed effects on PMN‐MDSC expansion and that (ii) alteration of the secreted lipid profile induced by Ch25h deletion in ECs under inflammatory conditions favors the expansion of PMN‐MDSC.

Given the importance of CD4^+^ T cells for EAE physiopathology and the lack of specific markers for PMN‐MDSC, we first asked whether CD11b^+^Ly6C^int^Ly6G^+^ cells isolated from the CNS of mice during EAE suppress CD4^+^ T cell proliferation. We sorted this population from the Bone Marrow (BM) and CNS of EAE wild‐type mice at the peak of the disease (gating strategy shown in Appendix Fig S3) and co‐cultured them with Carboxyfluorescein succinimidyl ester (CFSE) labeled CD4^+^ T cells isolated from the spleen and stimulated with anti‐CD3/CD28 beads. BM‐derived CD11b^+^Ly6C^int^Ly6G^+^ cells were not able to suppress CD4^+^ T cell proliferation (Fig 5C left panel) while CNS‐derived CD11b^+^Ly6C^int^Ly6G^+^ cells potently suppressed CD4^+^ T cell proliferation even when they were cultivated at the ratio of 1:8 (Fig 5C right panel). Those results suggest that the suppressive response is CNS‐specific. Moreover, we observed that the abundance of CNS‐derived CD11b^+^Ly6C^int^Ly6G^+^ cells population was negatively correlated with proliferating Ki67^+^CD4^+^CD44^+^ cells in the CNS during EAE (Fig 5D). These results indicate that CNS‐infiltrating CD11b^+^Ly6C^int^Ly6G^+^ cells at the peak of EAE can suppress CD4^+^ T cell proliferation and hence can be functionally characterized as PMN‐MDSC. To confirm that the CNS‐PMNs are MDSCs, we performed an arginase 1 (Arg1) staining on the blood, spleen, BM, and CNS. We observed that Arg1 expression was restricted to CNS live cells CD45^+^CD11b^+^Ly6C^int^Ly6G^+^ during EAE and was not detected in the same cells obtained simultaneously in blood, BM, or, spleen PMNs (Fig 5E with representative FACS staining and F). Those results suggest that CD45^+^CD11b^+^Ly6C^int^Ly6G^+^ acquire their suppressive properties in the CNS during EAE.

As *Ch25h* deletion in cultured CNS ECs under inflammatory conditions induced a lipid remodeling favoring PMN‐MDSC expansion, we assessed whether *Ch25h* ^ECKO^ mice display an increased infiltration of PMN‐MDSC in the CNS during EAE by FACS. In accordance with our *in vitro* results, we observed that *Ch25h* deletion in ECs resulted in increased infiltration of PMN‐MDSC in the CNS (Fig 5G illustrative FACS plots and H quantification) together with a reduction of CD4^+^CD44^+^Ki67^+^ T cells (Fig 5H top right panel). We did not observe significant differences in the abundance of these two populations using absolute numbers as readout (Fig 5H lower left panel). However, we observe an increased variance of both live cells CD45^+^CD4^+^CD44^+^KI67^+^ and live cells CD45^+^CD11b^+^Ly6C^int^Ly6G^+^ (PMN‐MDSC) in *Ch25h* ^ECKO^ mice compared with *Ch25h* ^fl/fl^ mice (*F* test, *P*‐values = 0.0053 and 0.0236, respectively). Furthermore, we observed that the ratio of PMN‐MDSC/proliferating CD4^+^ T cells is increased (Fig 5H lower right panel). These data suggest a remodeling of CNS‐infiltrating leukocytes in *Ch25h* ^ECKO^ mice in favor of PMN‐MDSC expansion.

**Figure EV2:**
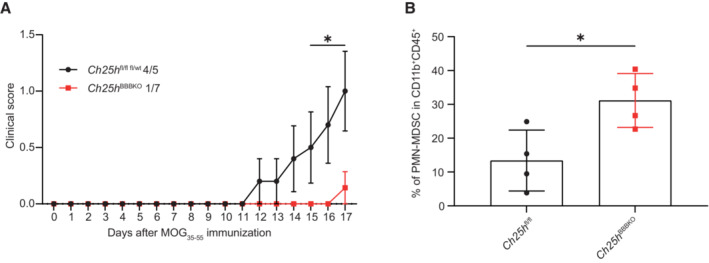
Related to Fig 5 CNS‐specific *Ch25h* ECs deletion promotes CNS PMN‐MDSC expansion EAE disease course in *Ch25h* ^BBBKO^ (*n* = 7 biological replicates) and Cre‐negative littermates (*Ch25h* ^fl/fl^, *n* = 5 biological replicates). Bars indicate mean ± SEM.Percentage of CNS PMN‐MDSC (live cells CD45^+^CD11b^+^Ly6C^int^Ly6G^+^) gated on CD45^+^CD11b^+^population assessed by flow cytometry in *Ch25h* ^BBBKO^ mice and *Ch25h* ^fl/fl^ at day 15 post‐immunization (*n* = 4 biological replicates/group). Symbols depict individual mice and bars indicate mean ± SD. Data information: *P < 0.05, ****P < 0.00005. *P*‐values were determined by two‐way ANOVA with Sidak's *post hoc* test (A) and by two‐tailed unpaired *t*‐test (B). The experiment was performed three times.

Finally, to confirm that our observations were specific to the CNS, we crossed our *Ch25h* ^fl/fl^ mice with the tamoxifen‐inducible BBB‐EC‐specific *Slco1c1‐CreER* ^*T2*^ mice (Ridder *et al*, 2011), allowing for CNS‐specific deletion of Ch25h in ECs, hereafter termed *Ch25h* ^BBBKO^ mice. Experimental autoimmune encephalomyelitis was induced in *Ch25h* ^BBBKO^ and control mice and we observed that the sole deletion of *Ch25h* in CNS ECs was sufficient to reproduce the EAE phenotype observed in the *Ch25h* ^ECKO^ and *Ch25h* ^BECKO^ mice (Fig 5I). We also observed a significant increase in CNS PMN‐MDSC in *Ch25h* ^BBBKO^ mice compared with *Ch25h* ^fl/fl^ mice (Fig EV2A EAE disease course with corresponding PMN‐MDSC staining in B). In conclusion, we found that Ch25h deletion in ECs promotes PMN‐MDSC accumulation in the CNS and that the attenuation of EAE mediated by *Ch25h* deletion in ECs is specific to the CNS.

### Mature neutrophil depletion promotes CNS PMN‐MDSC accumulation and protects *Ch25h* ^ECKO^ during EAE

**Figure 6:**
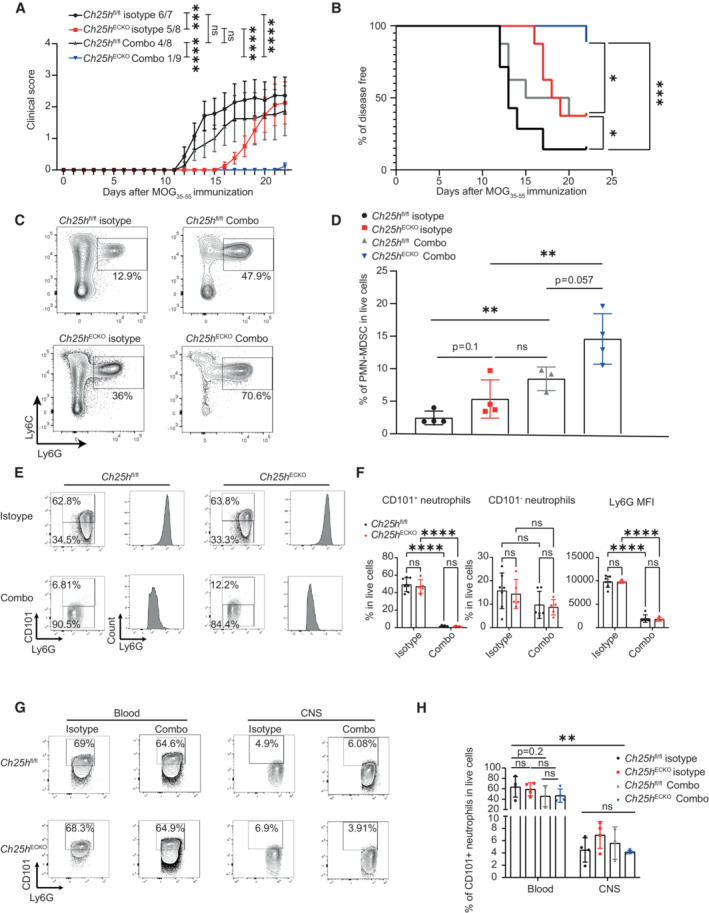
CD101^+^ neutrophils depletion protects *Ch25h* ^ECKO^ mice from EAE and favors CNS PMN‐MDSC accumulation EAE disease course in *Ch25h* ^fl/fl^ and *Ch25h* ^*ECKO*^ mice treated with isotype control antibody or Combo protocol (Ly6G^high^ cell depletion). Treatment was initiated on the day of first symptoms occurrence (Day 12 postimmunization). Symbols depict mean clinical score and bars mean ± SEM. *Ch25h* ^fl/fl^ isotype: *n* = 7, *Ch25h* ^ECKO^ isotype: *n* = 8, *Ch25h* ^fl/fl^ Combo: *n* = 8, *Ch25h* ^ECKO^ Combo: *n* = 9. The experiment was perfomed once.Survival analysis in the same conditions as in (A). *Ch25h* ^fl/fl^ isotype: *n* = 7, *Ch25h* ^ECKO^ isotype: *n* = 8, *Ch25h* ^fl/fl^ Combo: *n* = 8, *Ch25h* ^ECKO^ Combo: *n* = 9.Representative contour plots of PMN‐MDSC in the CNS at Day 22 of EAE assessed by flow cytometry in the same conditions as in (A).As in (C) except that statistical analysis and percentage of PMN‐MDSC in live cells are shown. Symbols depict individual mice and bars mean ± SD. *Ch25h* ^fl/fl^ Isotype *n* = 4, *Ch25h* ^ECKO^ isotype *n* = 4, *Ch25h* ^fl/fl^ Combo, *n* = 3, *Ch25h* ^ECKO^ Combo *n* = 4.Representative contour plots of CD101 and representative histograms of Ly6G expression in blood neutrophils at Day 16 of EAE assessed by flow cytometry. *Ch25h* ^ECKO^ and *Ch25h* ^fl/fl^ mice were treated with isotype or Combo treatment which was initiated at the first symptoms of EAE (Day 14 after immunization).As in (E) except that statistical analysis is shown. Symbols depict individual mice, bars mean ± SD. *Ch25h* ^fl/lfl^ isotype *n* = 7, *Ch25h* ^ECKO^ isotype *n* = 5, *Ch25h* ^fl/lfl^ Combo *n* = 6, *Ch25h* ^ECKO^ Combo *n* = 5. The experiment was performed once.Representative contour plots of CD101 expression in blood and CNS neutrophils of the same EAE mice as in (A) assessed by flow cytometry.As in (G) except that statistical analysis is shown. Symbols depict individual mice, bars indicate mean ± SD. *Ch25h* ^fl/lfl^ isotype *n* = 4, *Ch25h* ^ECKO^ isotype *n* = 4, *Ch25h* ^fl/lfl^ Combo *n* = 3, *Ch25h* ^ECKO^ Combo *n* = 4. The experiment was performed once. Data information: ns, nonsignificant, *P < 0.05, **P ≤ 0.005, ***P ≤ 0.0005, ****P ≤ 0.00005. P‐values were determined by two‐way ANOVA with Sidak's *post hoc* test (A, F, H), log‐rank (Mantel‐cox) test (B), and two‐tailed unpaired *t*‐test (D).

To further assess the function of PMN‐MDSC in EAE, we targeted surface Ly6G high cells by combining anti‐Ly6G antibody injections with a mouse anti‐rat IgG2a (so‐called “Combo treatment”; Boivin *et al*, 2020), a strategy aiming at depleting neutrophils and enhancing their turnover (Boivin *et al*, 2020). Experimental autoimmune encephalomyelitis was induced in *Ch25h* ^ECKO^ and *Ch25h* ^fl/fl^ mice, and each group was then divided into two arms receiving either the isotype or the Combo treatment during the symptomatic phase of EAE. Assessing the EAE disease course in the four groups described above, we first observed a significantly reduced severity in *Ch25h* ^ECKO^ compared with the *Ch25h* ^fl/fl^ mice in the isotype group, indicating that the treatment with the isotype control did not impact the phenotype previously observed in *Ch25h* ^ECKO^ mice (Fig 6A). Strikingly, the Combo treatment resulted in an almost complete EAE protection in *Ch25h* ^ECKO^ mice while it did not significantly alter the course of EAE in *Ch25h* ^fl/fl^ mice (Fig 6A). Remarkably, the incidence of EAE was reduced in the *Ch25h* ^ECKO^ Combo‐treated group (Fig 6B). Surprisingly, we observed an increased accumulation of CD45^+^CD11b^+^Ly6C^int^Ly6G intracellular^+^ granulocytes in the CNS of *Ch25h* ^ECKO^ Combo‐treated mice compared to *Ch25h* ^ECKO^ and *Ch25h* ^fl/fl^ isotype treated mice (Fig 6C and D).

To better evaluate the effect of the Combo treatment on neutrophils during EAE, we assessed their prevalence in the blood by flow cytometry 1.5 days after treatment initiation at the symptomatic phase of the disease. We observed that the Combo treatment differentially impacted the circulating pools of mature neutrophils (CD101^+^) and immature neutrophils (CD101^−^; Fig 6E and F and gating strategy Appendix Fig S4). Indeed, while almost no CD101^+^ neutrophils were detected at this time point, the circulation of CD101^−^ neutrophils was only mildly impacted (Fig 6E and F). Moreover, when comparing Ly6G Mean Fluorescence Intensity (MFI) in blood neutrophils, we found that the remaining circulating neutrophils in the Combo‐treated group displayed a significantly reduced surface expression, consistent with an immature phenotype (Fig 6E and F). After 10 days of treatment, we observed a trend toward a reduced prevalence of CD101^+^ neutrophils in blood (Fig 6G and H) but the impact of the Combo treatment did not reach significance. Additionally, CNS‐infiltrating neutrophils were immature as they expressed significantly less CD101 compared with blood 22 days after immunization (Fig 6G and H).

Those results show that the Combo protocol efficiently depletes mature neutrophils while immature neutrophils preferentially infiltrating the CNS are not strongly impacted. Neutrophil progenitors can be polarized in PMN‐MDSC (Veglia *et al*, 2021) that can further proliferate and acquire their suppressive phenotype directly in the CNS (Knier *et al*, 2018). We thus propose that reducing the mature‐to‐immature neutrophil ratio favors the accumulation and conversion of immature neutrophils in PMN‐MDSC in the CNS. Furthermore, *Ch25h* expression in endothelial cells negatively regulates the protective effect of PMN‐MDSC accumulation in EAE.

## Discussion

In this study, we demonstrated that Ch25h expression is increased in CNS endothelial cells during EAE and that 25‐OHC, the Ch25h‐downstream metabolite promotes neuroinflammation by reducing the expansion of PMN‐MDSC. Mechanistically, we propose that 25‐OHC and PGE_2_ are secreted by inflamed CNS endothelial cells and exert opposed effects on PMN‐MDSC. Hence, reduced levels of 25‐OHC and increased levels of PGE_2_ resulting from Ch25h deletion in endothelial cells act together to favor PMN‐MDSC expansion. Depleting mature neutrophils by using a double antibody‐based strategy during EAE promotes CNS PMN‐MDSC accumulation in absence of endothelial Ch25h. Taken together, our results reveal a novel function for both Ch25h and ECs in the regulation of PMN‐MDSC during neuroinflammation.

We observed that the sole deletion of Ch25h in endothelial cells was sufficient to reduce 25‐OHC levels, suggesting that Ch25h is the main enzyme involved in 25‐OHC secretion in this cell type. Indeed, Ch25h loss can be potentially compensated in other cell types such as hepatocytes by other enzymes displaying 25‐hydroxylase activity (e.g., Cyp3a4, Cyp27a1, and Cyp46a1; Honda *et al*, 2011). However, they were not produced by pMBMEC either at baseline nor under IL‐1β stimulation. Additionally, we previously showed that 25‐OHC levels were reduced in Ch25h KO Type 1 Regulatory T cells (Vigne *et al*, 2017). Hence, for these two cell types, Ch25h is the main 25‐OHC synthesizing enzyme.

Our *in vitro* data indicate that the inactivation of Ch25h affects local concentrations of 25‐OHC and related oxysterol including 7‐keto‐25‐OHC. Moreover, *Ch25h* was strongly upregulated in CNS ECs during EAE and *Ch25h*‐deficient brain endothelial cells displayed alterations in eicosanoids and oxysterols secretion (other than 25‐OHC and 7‐keto‐25‐OHC) only under inflammatory conditions. Finally, 25‐OHC primarily acts as a paracrine or autocrine mediator and its levels are increased in inflammatory conditions. Based on these data, our results suggest that locally, in the CNS, during EAE, deletion of Ch25h in ECs results in: (i) a reduction in the autocrine signaling of 25‐OHC, favoring PGE_2_ secretion and (ii) a reduction in 25‐OHC paracrine signaling. Ch25h expression by ECs regulates the PGE_2_ to 25‐OHC ratio, which might be an important determinant of PMN‐MDSC expansion in the inflamed CNS.

We observed that EC‐derived Ch25h and 25‐OHC promote inflammation during EAE. The role of Ch25h during inflammation is controversial, with reports suggesting both pro‐ and anti‐inflammatory functions (Gold *et al*, 2014; Reboldi *et al*, 2014; Chalmin *et al*, 2015; Dang *et al*, 2017; Wanke *et al*, 2017; Emgard *et al*, 2018; Jia *et al*, 2018; Wyss *et al*, 2019; Madenspacher *et al*, 2020; Russo *et al*, 2020). Those discrepancies could result from the different disease models used in these studies that investigated Ch25h function in various cell compartments and organs (e.g., colitis, lungs, and CNS inflammation). As for neuroinflammation, we have shown that Ch25h‐deficient mice depict an attenuated EAE disease (Chalmin *et al*, 2015) while others have reported an exacerbation of the same EAE model (Reboldi *et al*, 2014) but using different Ch25h‐deficient mouse strains and different controls. Indeed, in the later study, *Ch25h* heterozygous mice have been used as controls for Ch25h‐deficient mice. Thus, we cannot exclude that partial deletion of Ch25h also results in a phenotype *per se*. Furthermore, environmental factors such as gut microbiota (Berer *et al*, 2011), diet (Sonner *et al*, 2019), the month of birth (Reynolds *et al*, 2017), and genetic background (Sisay *et al*, 2013) can have a broad impact on EAE development and could contribute to the differences observed across different laboratories. However, our current study supports a pro‐inflammatory role for Ch25h during EAE.

We additionally found that the sole deletion of Ch25h in ECs was sufficient to dampen EAE. The cellular source of Ch25h is disputed during EAE as both moDCs (Chalmin *et al*, 2015) and microglial cells (Wanke *et al*, 2017) have been proposed to express *Ch25h*. ECs express Ch25h at high levels in lymph nodes and play a role in B cell positioning during a humoral response (Yi *et al*, 2012). In addition, Ch25h expression by ECs contributes to atherosclerosis development (Li *et al*, 2017). However, while Ch25h expression has been observed in ECs, and reported as upregulated in various murine models associated with a BBB dysfunction, its expression at the protein level and function during EAE had never been explored so far. Our data show that Ch25h expression in ECs plays a crucial role during EAE.

We here observed an increase in fatty acid desaturase 2 (FADS2) expression in the absence of Ch25h in ECs. FADS2 promotes the production of anti‐inflammatory lipids (Liu *et al*, 2020). These data prompted us to speculate that FADS2 increased activity contributes to the attenuated EAE phenotype observed in the absence of Ch25h in ECs. Mechanistically, 25‐OHC restrains cholesterol synthesis through inhibition of Sterol Regulatory Element Binding Transcription Factor 2 (SREBP2; Waltl *et al*, 2013) and FADS2 has been described as a target of SREBP2 (Triki *et al*, 2020). We hence propose that the reduction in 25‐OHC in ECs induced by Ch25h deletion increases FADS2 expression through the release of SREBP2 inhibition. Interestingly, two single nucleotide polymorphisms (SNPs) within the FADS2 gene locus have been associated with a reduced risk of multiple sclerosis (Langer‐Gould *et al*, 2020). However, the impact of these SNPs on FADS2 activity remains unknown. Additionally, the role of FADS2 in EAE and ECs is virtually unexplored. We here establish that IL‐1β signaling in ECs upregulates FADS2 expression and that Ch25h is an upstream regulator of this enzyme. Moreover, our results suggest that the increased activity of FADS2 in ECs favors PMN‐MDSC expansion through PGE_2_ secretion and could be a protective mechanism in EAE. In line with this, PGE_2_ is increased in the CNS of mice during EAE (Kihara *et al*, 2009) and injections of a stable form of PGE_2_ or an agonist of its receptor E‐type Prostanoid receptor 4 (EP4) protects mice from EAE (Esaki *et al*, 2010). Diet supplementation with a plant containing high levels of γ‐linoleic acid has been shown to attenuate EAE and increase PGE_2_ production by splenocytes in SJL mice (Harbige *et al*, 2000). As mentioned earlier, FADS2 is the rate‐limiting enzyme for the synthesis of γ‐linoleic acid, which is a precursor of prostaglandins (Nakamura & Nara, 2004). Furthermore, another isoform of SREBP; SREBP‐1a, has also been described to regulate FADS2 synthesis and γ‐linoleic acid production (Horton *et al*, 2003; Dong *et al*, 2017). Therefore, increased SREBP‐1a activity under reduced 25‐OHC conditions might also explain our results. Additional studies will be necessary to further clarify the role of FADS2 during neuroinflammation.

We discovered that Ch25h deletion in ECs resulted in an expansion of PMN‐MDSC in the CNS during EAE. In mice, the CD11b^+^Ly6C^int^Ly6G^+^ population can be defined both as bona fide neutrophils and PMN‐MDSC (Bronte *et al*, 2016). Hence, the expression of these markers is not sufficient to ensure the suppressive phenotype of these cells. Ly6G^+^ cells isolated from the CNS during the recovery phase of the EAE suppress B cell proliferation (Knier *et al*, 2018). We here show that CD11b^+^Ly6C^int^Ly6G^+^ isolated solely from the CNS at the peak of the disease express arginase‐1, a key enzyme in PMN‐MDSC‐mediated lymphocyte suppression, and can suppress CD4^+^ T cell proliferation, suggesting that this population at this time point of EAE corresponds to PMN‐MDSCs. However, it cannot be excluded that CD11b^+^Ly6C^int^Ly6G^+^ cells are heterogeneous and contain both bona fide neutrophils and PMN‐MDSC and that the suppressive capacity of this population is dependent on the relative proportion of these two subsets. In line with this, single‐cell RNA‐seq analysis of Ly6G^+^ cells isolated from the CNS in a mouse model of optic nerve injury identified three different cell clusters (Sas *et al*, 2020), suggesting that CNS‐infiltrating neutrophils are a heterogeneous population. Interestingly, arginase 1 expression was restricted to a cluster displaying a transcriptomic signature consistent with immature neutrophils (Sas *et al*, 2020). These cells were CD101^−^, as the vast majority (up to 92%) of CD11b^+^Ly6C^int^Ly6G^+^ cells infiltrating the CNS at the plateau/recovery phase of EAE in our study. Moreover, Ly6G^+^ cells isolated from the CNS at the onset and the recovery phase of EAE have a distinct transcriptomic profile and the PMN‐MDSC transcriptomic signature seems restricted to Ly6G^+^ cells from the EAE recovery phase (Knier *et al*, 2018). In other words, the maturation status of CNS‐infiltrating neutrophils could determine whether they will acquire a suppressive phenotype. When we depleted neutrophils using the “Combo protocol” at the time of the first EAE symptoms, we observed almost complete EAE protection in *Ch25h* ^ECKO^ mice while we did not observe significant protection in the control group. We also observed a paradoxical accumulation of CD11b^+^Ly6C^int^Ly6G^+^ in the CNS. We propose that the increased circulation of immature neutrophils relative to mature neutrophils observed in the Combo‐treated group favors the accumulation of PMN‐MDSC precursors in the CNS. However, we do not exclude that neutrophil depletion earlier in the disease course could explain this protection. Indeed, we previously showed that Ch25h KO mice display a delayed infiltration of Th17 cells in the CNS (Chalmin *et al*, 2015) and others have shown that Th17 cells can promote EAE by favoring neutrophil infiltration (McGinley *et al*, 2020). Hence, the protection could also be explained by a synergistic effect of delayed Th17 cell infiltration in *Ch25h* ^ECKO^ mice and neutrophil depletion mediated by the Combo protocol. However, the striking infiltration of CD11b^+^Ly6C^int^Ly6G^+^ cells in *Ch25h* ^ECKO^ Combo‐treated mice that did not display any symptoms does not support this hypothesis.

Overall, our results demonstrate a novel function of Ch25h and ECs in the regulation of PMN‐MDSC expansion during neuroinflammation. The fact that IL‐1β can upregulate Ch25h in pMBMECs and that Ch25h has been described to be upregulated in CNS ECs in other disease models suggests that the same mechanisms may be relevant in other pathologies. We thus propose that targeting ECs and the Ch25h pathway could be promising approaches to target inflammatory diseases.

## Materials and Methods

### Fluorescent *in‐situ* hybridization

Fluorescent *in‐situ* hybridization (FISH) was carried out using the RNAscope Fluorescent Multiplex Kit V2 (323110, Advanced Cell Diagnostics, Inc.). *In‐situ* hybridization protocol was performed following recommended specifications for murine formalin‐fixed paraffin‐embedded (FFPE) brain tissue. Probe against murine *CH25H* (424561) was commercially available from Advanced Cell Diagnostics, Inc. RNAscope. FISH protocol on murine brains was followed by fluorescence immunostaining for Iba1 and IsolectineB4. For image acquisition, slides were scanned with the Pannoramic 250 FLASH II (3DHISTECH) Digital Slide Scanner at 20× magnification.

### Mice

Ch25h‐eGFP^fl/fl^ mice (Fig 1A): These mice were generated by Cyagen as follow: a constitutive Knock‐In (KI) with conditional knockout (KO), using a floxed‐reporter *Ch25h* knock‐in, with eGFP was used as a reporter protein fused to the 3′ end of Ch25h. Furthermore, the entire gene was flanked with LoxP sites, taking care to avoid promoter disruption. Linker‐eGFP reporter has been inserted in the targeting cassette and is thus not expressed as a fusion protein before cre‐recombination.

*Cdh5*‐CreER^T2^ (MGI:3848982), *Pdgfb*‐iCreER^T2^ (MGI:3793852) mice, *Prox1*‐CreER^T2^ (MGI:5617984), and *Slco1c1*‐CreER^T2^ (MGI:5301361) mice were reported previously (PMID: 19144989, 10.1002/dvg.20367, 10.1172/JCI58050; Ridder *et al*, 2011). Wild‐type mice were obtained from Charles‐River Laboratories. All mouse strains were on pure C57BL/6 background. Eight to 12 weeks mice were used for all experiments. Animals were kept in a specific pathogen‐free facility at the Lausanne University. All experiments were carried out in respect with guidelines from the Cantonal Veterinary Service of the state of Vaud.

### EAE, tamoxifen injections, and Combo protocol

For induction of EAE, female mice were immunized with 100 μg myelin oligodendrocyte glycoprotein peptide 35–55 (MOG_35_–_55_, Anawa) in complete Freund's adjuvant supplemented with 5 mg/ml *Mycobacterium tuberculo*sis H37Ra (BD Difco). Two hundred microliters of emulsion was subcutaneously injected into four sites on the flanks of mice. At Days 0 and 2, after initial MOG_35‐55_ injections, mice received intravenous injection of 100 ng pertussis toxin (Sigma Aldrich). Mice were weighed and scored daily using the following system: 0: no symptom, 1: tail paralysis, 2: hind limb paresis, 2.5: partial hind limb paralysis, 3: Complete hind limb paralysis, 4: forelimb paresis and complete hind paralysis, 5: moribund or dead. Clinical scores were assessed by a blinded investigator.

For tamoxifen injections, mice between 8 and 10 weeks were injected intraperitoneally with tamoxifen in Koliphor (Sigma Aldrich) twice a day with a total of 2 mg/mice/day for 4 consecutive days. Two weeks of washout period were performed before EAE induction.

The Combo protocol was performed as described by Boivin *et al* (2020). Briefly, 25 μg of Anti‐Ly6G (clone 1A8, Bio X cell) antibody or isotype control (Rat IgG2a, Bio X cell) were injected intraperitoneally every day for 10 consecutive days, starting from the first symptoms of EAE. Every other day, mice were injected intraperitoneally with 50 μg of anti‐rat Kappa immunoglobulin (Clone MAR 18.5 Bio X cell) or Isotype control.

### Histology

Mice were perfused with cold PBS followed by 4% paraformaldehyde fixation. Spinal cord tissue was embedded in paraffin. For light microscopy, sections were stained with hematoxylin and eosin (HE; Sigma–Aldrich). Tissue sections were scanned using a Nanozoomer S60 or Pannoramic P250 Flash II whole slide scanner. Inflammatory foci per spinal cord were quantified on HE‐stained cross‐sections. Average values of five cross‐sections per animal were calculated. Quantifications were performed by two independent blinded investigators.

### Isolation of leukocytes and ECs from the CNS

For CNS preparation, mice were perfused through the left ventricle with cold PBS (Bichsel). Brains were dissected and spinal cords extruded by flushing the vertebral canal with cold PBS. CNS tissue was cut into pieces and digested for 45 min, in a DMEM containing collagenase D (2.5 mg/ml Sigma) and Dnase 1 (1 mg/ml Sigma) to give a single‐cell suspension. For ECs, meninges were removed before brain enzymatic digestion with Collagenase/Dispase (2 mg/ml), DNAse I (10 μg/ml) and Nα‐Tosyl‐L‐Lysin‐chlormethyl keton hydrochlorid (TLCK, 0.147 μg/ml). Mononuclear and ECs were isolated by passage of the tissue through a cell strainer (70 μm), followed by Percoll gradient centrifugation (70%/37% for CNS leukocytes and 37% for ECs). Leukocytes were removed from the interphase and for ECs the entire 37% Percoll suspension was collected, washed, and resuspended in culture medium for further analyses.

### Flow cytometry, cell sorting, and suppression assay

Single cells were suspended in PBS and then stained with LIVE/DEAD fixable Red stain kit (Invitrogen) according to the manufacturer's instructions. For extracellular staining, cells were incubated with anti‐CD16/32 (Invitrogen) in PBS+ 1% BSA and then stained with anti‐mouse fluorochrome‐conjugated antibodies: CD45 (30‐F11), CD3 (45‐2C11), CD44 (IM7), CD11b (M1/70), Ly6G (1A8), Ly6C (HK 1.4), TER‐119 (TER‐119), purchased from Biolegend, CD13 (123–242), CD4 (RM4‐5), purchased from BD Biosciences, CD31 (390), CD101 (Moushl/101), purchased from Invitrogen, at 4°C for 30 min. For intracellular staining, after surface staining, cells were fixed and permeabilized using Foxp3/transcription factor staining kit (Invitrogen) according to the manufacturer's protocol and then incubated with anti‐mouse fluorochrome‐conjugated antibodies: Ki67 (SolA15, Invitrogen), Ly6G (1A8), and Arg1 (A1exF5, Invitrogen) for 30 min. Samples were all acquired on a LSR‐II cytometer (BD Bioscience). For PMN‐MDSC suppression assay, PMN‐MDSC were isolated from the CNS by FACS sorting using specific fluorochrome‐conjugated antibodies. Isolated PMN‐MDSC were then co‐cultured at different ratios as indicated in the figure with purified CD4^+^ T (CD4^+^ T cell isolation Kit, Miltenyi Biotech, 1.10^4^ cells/well) previously labeled with 5 μM carboxyfluorescein succinimidyl ester (CFSE, Invitrogen). Co‐culture were stimulated with plate‐bound anti‐CD3/anti‐CD28 antibodies (1 μg/ml, BioXcell) for 72 h. The proliferative levels of CFSE‐CD4^+^ T cells were evaluated by the rates and intensity of CFSE dilution measured with flow cytometry.

### Isolation and culture of primary brain microvascular cells

Isolation and culture of primary mouse brain microvascular endothelial cells (pMBMECs) from 7–12‐week‐old female mice were performed as previously described (Coisne *et al*, 2005). Briefly, mice were euthanized by cervical dislocation, brains were dissected, and meninges, olfactory bulb, brainstem, and thalami were removed. A minimum of six brains per genotype were pooled, homogenized, and resuspended in a 30% dextran (Sigma) solution to obtain a final concentration of 15% dextran. Samples were centrifuged, the vascular pellet was collected, and then digested by incubation with Collagenase/Dispase (2 mg/ml), DNAse I (10 μg/ml) and Nα‐Tosyl‐L‐Lysin‐chlormethyl keton hydrochlorid (TLCK, 0.147 μg/ml) for 30 min at 37°C in a shaker incubator. Digested vessel fragments were then plated in Matrigel (Corning)‐coated 96‐well Nunclon Delta Surface (Thermo Scientific) plates at a seeding density of 51,000 digested capillaries/cm^2^ onto matrigel‐coated wells. pMBMECs were maintained in Dubelco's Modified Eagle Medium (DMEM, Gibco) with 10% FCS (Biowest), 50 μg/ml of gentamicin (Sigma), and 1 ng/ml of basic fibroblast growth factor (Sigma) at 37°C and 5% CO_2_ in a humidified incubator. Once confluent, cells were treated with either mouse recombinant IL‐1β (R&D systems, 10 ng/ml) or vehicle for 24 h.

### RT‐qPCR

Total RNA was extracted using the RNeasy Mini Kit (Qiagen) according to the manufacturer's protocol. cDNA was produced from RNA without amplification using the Superscript II RT (Invitrogen). PCR products were amplified with the PowerUp SYBR Green Master Mix (Applied Biosystem). Samples were analyzed on the StepOne Real‐Time PCR System. Glyceraldehyde 3‐phosphate dehydrogenase (GAPDH) or β‐actin was used as reference gene, and the comparative CT method was employed to evaluate relative mRNA expression. Primers were purchased from Microsynth AG (Balgach, Switzerland). The following primers were used: *GAPDH* (forward) 5′‐TGT GAA CGG ATT TGG CCG TA‐3′, (reverse) 5′‐ACT GTG CCG TTG AAT TTG CC‐3′, *β‐actin* (forward) 5′‐AAG TGT GAC GTT GAC ATC CGT AAA‐3′, (reverse) 5′‐CAG CTC AGT AAC AGT CCG CCT AGA‐3′, *Ch25h* (forward) 5′‐TGC ATC ACC AGA ACT CGT CC‐3′, (reverse) 5′‐TCA GCA CGT CGA AGA AGG‐3′, *FADS2* (forward) 5′‐CCC TTC TGT CCC ACA GAC AC‐3′, (reverse) 5′‐CCA GCC ATG GGA AAG ACA CT‐3′, *FADS3* (forward) 5′‐TCC TTG CCT AGG GGA GTA CC‐3′, (reverse) 5′‐AGC AGA CAC GTG ACT TAG CC‐3′, *ELOVL4* (forward) 5′‐TGG TAT CTC ATC GAA CGG CG‐3′, (reverse) 5′‐AGA CCC TCT GCT TCT GTT GC‐3′, *PTGIS* (forward) 5′‐GGT TGA GAA TCC TGC GGT CC‐3′, (reverse) 5′‐CTG GCA GCA TCT CTC CCA AA‐3′.

### RNA sequencing

*Ch25h* ^fl/fl^ and *Ch25h* ^ECKO^ female mice were injected with tamoxifen. Nine brains per genotype were pooled and primary brain microvascular endothelial cells (pMBMEC) were isolated and plated in a 96‐well plate (2 wells/brain). Confluent pMBMEC were left unstimulated or stimulated IL‐1β for 24 h. RNA of three wells was pooled to obtain one replicate for RNA sequencing. The Lausanne Genomic Technologies Facility performed the RNA‐seq. RNA quality was assessed on a Fragment Analyzer (Agilent Technologies), and all RNAs had a RQN between 8.7 and 10. RNA‐seq libraries were prepared from 500 ng of total RNA with the Illumina TruSeq Stranded mRNA reagents (Illumina) using a unique dual indexing strategy, and following the official protocol automated on a Sciclone liquid handling robot (PerkinElmer). Libraries were quantified by a fluorometric method (QubIT, Life Technologies) and their quality assessed on a Fragment Analyzer (Agilent Technologies).

Cluster generation was performed with 2 nM of an equimolar pool from the resulting libraries using the Illumina HiSeq 3000/4000 SR Cluster Kit reagents and sequenced on the Illumina HiSeq 4000 using HiSeq 3000/4000 SBS Kit reagents for 150 cycles (single end). Sequencing data were demultiplexed using the bcl2fastq2 Conversion Software (version 2.20, Illumina).

### Oxysterol measurements

Oxysterols were analyzed using a validated HPLC–MS method (Mutemberezi *et al*, 2016b). Briefly, 200 μl of cell supernatants was placed in glass vials containing d_7_‐4β‐hydroxycholesterol (133.3 pmol) and d_7_‐24‐hydroxycholesterol (200 pmol) as internal standards (Avanti Polar Lipids) as well as dichloromethane, methanol (containing 10 μg of butylated hydroxytoluene), and bidistilled water (containing 20 ng ethylenediaminetetraacetic acid; 8:4:2 v/v/v). After mixing and sonication, samples were centrifuged and the organic phase was recovered and dried under a nitrogen stream. The organic residue was resuspended and prepurified by solid phase extraction over silica. The eluate containing oxysterols was analyzed by HPLC‐MS using an LTQ‐Orbitrap XL MS (Thermo Fisher Scientific) coupled to an Accela HPLC system (Thermo Fisher Scientific). Chromatographic separation was performed using an Ascentis Express C‐18 column (2.7 μm, 150 × 4.6 mm, Sigma), kept at 15°C. Mobile phase was a gradient of methanol and water containing acetic acid. MS analyses were performed using an atmospheric pressure chemical ionization source in the positive mode. Data are expressed in nanomolar.

### Eicosanoids measurements

Supernatants (150 μl) from brain endothelial cells (cultured in IL‐1β stimulated and nonstimulated conditions) were mixed with 150 μl of extraction buffer (citric acid/Na_2_HPO_4_, pH = 5.6) and 10 μl of internal standard solution and extracted by solid phase extraction using an OASIS HLB LP 96‐well plates 60 μm (60 mg). Wells were conditioned and equilibrated with 1 ml of methanol and 1 ml of water, respectively. Loaded samples were washed with water/methanol (90:10 v/v), and eicosanoids were eluted with 750 μl of methanol. Then, solvent was evaporated to dryness under N2 gas (TurboVap, Biotage), and final extracts were reconstituted with 75 μl of methanol/water (6:1, v/v).

Extracted samples were analyzed by Reversed Phase Liquid Chromatography coupled to tandem mass spectrometry (RPLC – MS/MS; Kolmert *et al*, 2018) in negative ionization modes using a 6495 triple quadrupole system (QqQ) interfaced with 1290 UHPLC system (Agilent Technologies). The chromatographic separation was carried out in an Acquity BEH C18, 1.7 μm, 150 × 2.1 mm I.D. column (Waters, Massachusetts, US). Mobile phase was composed of A = water with 0.1% acetic acid and B = acetonitrile/isopropanol 90:10 v/v at a flow rate of 500 μl/min, column temperature 60°C and sample injection volume 2 μl. Gradient elution was performed with 80% of A as the starting condition, linearly decreased to 65% at 2.5 min, to 60% at 4.5 min, to 58% at 6 min, to 50% at 8 min, to 35% at 14 min, to 27.5% at 15.5 min, and to 0% at 16.6 min. The column was then washed with solvent B for 0.9 min and equilibrated to initial conditions. ESI source conditions were set as follows: dry gas temperature 290°C, nebulizer 25 psi and flow 12 l/min, sheath gas temperature 400°C and flow 12 l/min, nozzle voltage 2,000 V, and capillary voltage 3,000 V. Dynamic Multiple Reaction Monitoring (DMRM) was used as acquisition mode with a total cycle time of 250 ms. Optimized collision energies for each metabolite were applied (Kolmert *et al*, 2018).

Raw LC‐MS/MS data were processed using the Agilent Quantitative analysis software (version B.07.00, MassHunter Agilent technologies). Peak area integration was manually curated and corrected when necessary. Concentrations were calculated using the calibration curves and the ratio of MS response between the analyte and the stable isotope‐labeled internal standard (IS; Dataset EV5 for the list and references of all internal standards used), to correct for matrix effects.

The following metabolites were measured: 13‐HODE, 9‐HODE, 12(13)‐EpOME, 12,13‐DiHOME, 12‐HETE, 15‐HETE, 5‐HETE, 9(10)‐EpOME, 9,10,13‐TriHOME, 9,10‐DiHOME, 9,12,13‐TriHOME, 9‐HETE, 13‐KODE, 9‐KODE, 12‐HEPE, 15‐HEPE, 14‐HDoHE, 17‐HDoHE, 14,15‐DiHETE, EKODE, Arachidonic acid, Linoleic Acid, Docosahexaenoic Acid, Eicosapentaenoic Acid, TXB2, 11B‐PGF_2a_, 15‐deoxy‐PGJ_2_, 19,20‐DiHDPA, 8‐HETE, 11‐HEPE, PGB_2_, PGD_1_, 12‐KETE, 9‐HEPE, 20‐HETE, 8‐HEPE, 11‐HETE, 8‐HETrE, 15(S)‐HETrE, 20‐COOH‐LTB4, 5,6‐DiHETrE, 15‐KETE, 5‐HEPE, 8‐iso‐PGE_2_, LTB3, LTB4, PGE_1_,6‐keto‐PGF_1a_, PGD_2_, PGD_3_, PGE_2_, 10,17‐DiHDoHE, 11‐HDoHE, δ12‐PGJ_2_, 17(R)‐Resolvin‐D1, 11(12)‐EpETrE, PGF_2a_, 11,12‐DiHETrE, 14,15‐DiHETrE, PGE_3_, PGJ_2_, Resolvin D1, Resolvin D2, 12(13)‐EpODE,12(S)‐HHTrE, LTE4, 5‐HETrE, 14(15)‐EpETE, 14(15)‐EpETrE, 6‐trans‐LTB4, 8,9‐DiHETrE, 16(17)‐EpDPE, 17(18)‐EpETE, 17,18‐DiHETE, 19(20)‐EpDPE, 5(6)‐EpETrE, 5,15‐DiHETE, 5,6‐DiHETE, 5‐KETE, 8(9)‐EpETrE, 9‐HOTrE, 8‐HDoHE, 9‐KOTrE, 20‐OH‐LTB4, 13‐HOTrE, 8,15‐DiHETE, LTC4, 14,15‐LTC4, LTD4, LXA4, LXA5, LXB4, 18‐HEPE, 7,17‐hydroxy DPA, 7‐Maresin‐1, tetranor‐PGDM, 13,14‐dihydro‐15‐keto PGE_2_, tetranor‐PGEM, iPF2α‐IV, 5‐iPF2α‐VI, 8‐iso‐PGF_2α_, 8‐iso‐PGF_3α_, 11‐dehydro TXB2, TXB3, 11‐dehydro TXB3, 4‐HDoHE, 11‐HEDE, 15‐HEDE, 13‐HOTrE(γ), 15‐epi Lipoxin A4, TXB1, FOG9, 19‐HETE, 15‐OxoEDE, 14,15‐LTE4.

### Bone marrow‐derived cell culture and MDSC polarization

Tibias and femurs from male and female WT C57BL/6 were dissected, bone marrow was flushed and Red Blood Cells (RBC) lysed with RBC lysis buffer (Invitrogen) according to the manufacturer's protocol. 100,000 cells per well were plated in 24‐wells plates and were cultivated during 4 days with mouse recombinant GM‐CSF (Immunotools, 40 ng/ml) mouse recombinant IL‐6 (Peprotech, 40 ng/ml) and with either ethanol control, Prostaglandin E2 (Sigma‐Aldrich, 20 nM) or 25‐Hydroxycholesterol (Sigma‐Aldrich, 1 μM). At the end of the experiment, adherent and nonadherent cells were collected and processed for flow cytometry as explained above.

### Statistical analysis

Data analyses and graphs were performed using the GraphPad Prism software for Windows (GraphPad Software Inc., San Diego, CA, USA). A *P*‐value < 0.05 was considered as significant. *P*‐values of cell frequency, mRNA levels, and oxysterols or prostaglandins concentrations were determined by either unpaired Student *t*‐test or two‐way ANOVA with Sidak's *post hoc* test as specified in the legends. Comparison of EAE clinical scores was assessed with two‐way ANOVA with Sidak's *post hoc* test, EAE incidence with log‐rank (Mantel‐cox) test, Area Under Curve (AUC) was calculated with the AUC function of Graphpad Prism and *P*‐values determined by unpaired Student *t*‐test. Principal component analysis (PCA) and its associated loading plot were determined using the PCA function of GraphPad Prism. The sample size for EAE is based on the EAE expertise in our laboratory (previous power calculation). The sample size for each experiment is specified in the figures legends.

Preprocessing and statistical analysis of the RNA sequencing were performed by The Lausanne Genomic Technologies Facility with R (R version 3.6.1). For data processing, purity‐filtered reads were adapter‐ and quality‐trimmed with Cutadapt (v. 1.8, Martin, 2011). Reads matching to ribosomal RNA sequences were removed with fastq_screen (v. 0.11.1). Remaining reads were further filtered for low complexity with reaper (v. 15‐065, Davis *et al*, 2013). Reads were aligned against the *Mus musculus* GRCm38.98 genome using STAR (v. 2.5.3a, Dobin *et al*, 2013). The number of read counts per gene locus was summarized with htseq‐count (v. 0.9.1, Anders *et al*, 2015) using the *Mus musculus* GRCm38.92 gene annotation. Quality of the RNA‐seq data alignment was assessed using RSeQC (v. 2.3.7, Wang *et al*, 2012). Genes with low counts were filtered out according to the rule of 1 count(s) per million (cpm) in at least one sample. Library sizes were scaled using TMM normalization. Subsequently, the normalized counts were transformed to cpm values, and a log_2_ transformation was applied, by means of the function cpm with the parameter setting prior.counts = 1 (edgeR v 3.28.0; Robinson *et al*, 2010). After data normalization, a quality control analysis was performed through sample density distribution plots, hierarchical clustering, and sample PCA. Differential expression was computed with the R Bioconductor package limma (v. 3.42) by fitting data to a linear model. The approach limma‐trend was used. Fold changes were computed and a moderated *t*‐test was applied for pairwise comparison of selected conditions and for the interaction between the IL‐1β treatment effect and the genotype (*Ch25h* ^ECKO^ vs. *Ch25h* ^fl/fl^) effect. *P*‐values were adjusted globally on all resulting gene lists together, using the Benjamini–Hochberg (BH) method, which controls for the false discovery rate (FDR). Gene set enrichment analysis (GSEA) was conducted according to the method described by Subramanian *et al* (2005) against gene sets of the Gene Ontology (GO; Ashburner *et al*, 2000; Gene Ontology, 2021) Biological Processes. Gene set enrichment analysis was performed using the clusterProfiler (v.4.0.5; Yu *et al*, 2012) and the org.Mm.eg.db (v.3.13.0) packages within R (v.4.1.0). For each pairwise condition comparison, genes were sorted according to decreasing *t*‐statistic, and provided to the “gseaGO” function, using parameters eps = 1e‐60, minGSSize = 25, seed = T, and a seed set to 1,234. The list of gene sets with adjusted *P*‐value < 0.05 was manually parsed and representative GO terms were selected to create a dotplot of normalized enrichment scores using ggplot2 (v.3.3.5).

Gene set enrichment analysis (GSEA) was also performed with preranked gene list function of the GSEA software from the Broad Institute (Mootha *et al*, 2003; Subramanian *et al*, 2005) using the *t*‐statistic for ranking the input gene lists. Unsaturated fatty acid biosynthetic process was assessed using the Gene Ontology Biological Process (GO: BP) collection.

## Author contributions

**Florian Ruiz:** Conceptualization; data curation; formal analysis; investigation; methodology; writing – original draft; project administration. **Benjamin Peter:** Data curation; formal analysis; supervision; investigation; methodology; writing – review and editing. **Jessica Rebeaud:** Data curation; formal analysis; investigation; methodology; writing – review and editing. **Solenne Vigne:** Data curation; formal analysis; supervision; investigation; methodology; writing – review and editing. **Valentine Bressoud:** Formal analysis; methodology; writing – review and editing. **Martin Roumain:** Data curation; formal analysis; investigation. **Tania Wyss:** Formal analysis; methodology; writing – review and editing. **Yannick Yersin:** Data curation; formal analysis; investigation. **Ingrid Wagner:** Data curation. **Mario Kreutzfeldt:** Conceptualization; data curation; formal analysis. **Marisa Pimentel Mendes:** Formal analysis. **Camille Kowalski:** Data curation; formal analysis. **Gael Boivin:** Conceptualization; data curation. **Léonard Roth:** Data curation; formal analysis. **Markus Schwaninger:** Data curation. **Doron Merkler:** Conceptualization; formal analysis; supervision. **Giulio G Muccioli:** Formal analysis; methodology. **Stephanie Hugues:** Conceptualization; Data curation; formal analysis; funding acquisition. **Tatiana V Petrova:** Conceptualization; resources; supervision; funding acquisition. **Caroline Pot:** Conceptualization; resources; data curation; formal analysis; supervision; funding acquisition; validation; visualization; methodology; writing – original draft; project administration; writing – review and editing.

## Disclosure and competing interests statement

The Authors declare that they have no conflict of interest.

## Ethics approval

All experiments were performed in accordance with guidelines from the Cantonal Veterinary Service of state Vaud (authorization #VD3393, #VD3767).

## Supporting information



Appendix

Expanded View Figures PDF

Dataset EV1

Dataset EV2

Dataset EV3

Dataset EV4

Dataset EV5

PDF+

## Data Availability

All data, code, and materials used in the analysis are available to any researcher for purposes of reproducing or extending the analysis. All data are available in the main text or the Supporting Information. RNA‐seq data produced in this study have been submitted to Gene Expression Omnibus (GEO) and are available under the accession number GSE217431 (http://www.ncbi.nlm.nih.gov/geo/query/acc.cgi?acc=GSE217431).
