# Supplementary material for: Endothelial cell‐derived oxysterol ablation attenuates experimental autoimmune encephalomyelitis
Source: EMBO Rep. 2023 Jan 30;24(3):e55328. doi: 10.15252/embr.202255328 (PMC9986812; doi:10.15252/embr.202255328)
Supplement: Supplementary file 1 — Appendix [file EMBR-24-e55328-s005.pdf]

Appendix:

Endothelial cell-derived oxysterol ablation attenuates experimental autoimmune encephalomyelitis

Ruiz Florian, Peter Benjamin, Rebeaud Jessica, Vigne Solenne, Bressoud Valentine, Roumain Martin, Wyss Tania, Yersin Yannick, Wagner Ingrid, Kreutzfeldt Mario, Pimentel Mendes Marisa, Kowalski Camille, Boivin Gael, Roth Leonard, Schwaninger Markus, Merkler Doron, Muccioli Giulio G., Hugues Stephanie, Petrova Tatiana V, Pot Caroline

Table of content

Page
Appendix Figure S1…………………………………….………. 2
Appendix Figure S2………………………………...…...........4
Appendix Figure S3………………………………...…...........5
Appendix Figure S4………………………………...…...........6

Appendix Figure S1

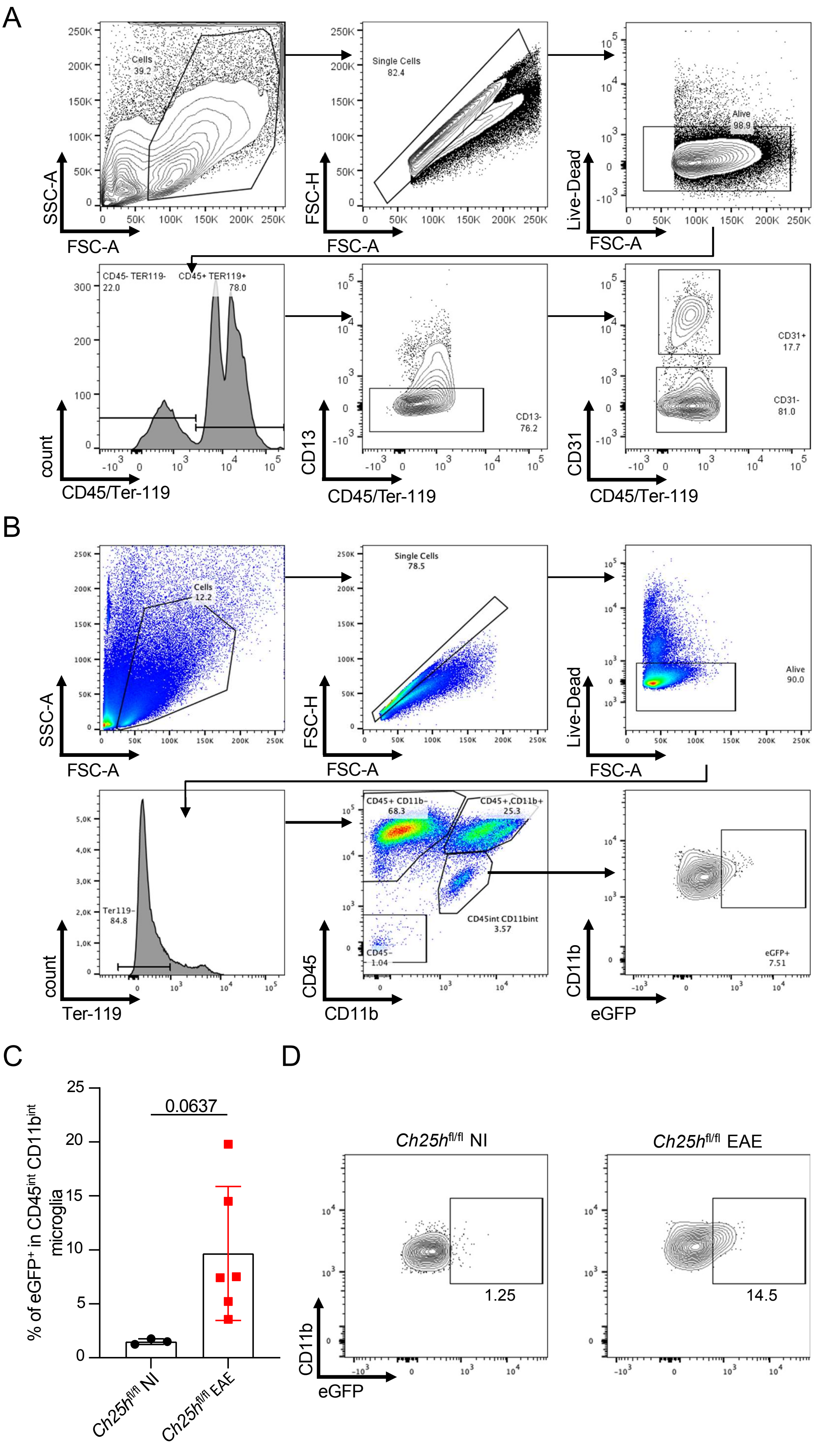

Appendix Figure S1. Related to Figure 1. Analysis of Ch25h-eGFP expression in the CNS. (A) Gating strategy for flow cytometry analysis of Ch25h-eGFP expression in cells of the CNS. Total cells were selected based on Forward Scatter (FSC-A) and side scatter plot (SSC-A). Doublet and dead cells were excluded. For endothelial cells and CNS resident cells (excluding microglial cells), $CD45^{-}TER119^{-}$ cells were selected. $CD13^{+}$ cells were excluded to avoid pericyte contamination. Endothelial cells were defined by CD31 expression and other CNS resident cells by the absence of CD31. **(B)** Gating strategy for flow cytometry analysis of Ch25h-eGFP expression in $TER119^{-}CD45^{int}$ $CD11b^{int}$ microglial cells. **(C)** Flow cytometry analysis of Ch25h-eGFP expression in $TER119^{-}CD45^{int}CD11b^{int}$ microglia in non-immunized (NI) *Ch25h*$^{fl/fl}$ mice (n=3 biological replicates) and at day 17 post-immunization (*Ch25h*$^{fl/fl}$ EAE, n= 6 biological replicates). Bars indicate mean ± SD. P values were determined by unpaired Student's t test. **(D)** Representative FACS plot of Ch25h-eGFP in NI and EAE *Ch25h*$^{fl/fl}$ mice.

Appendix Figure S2

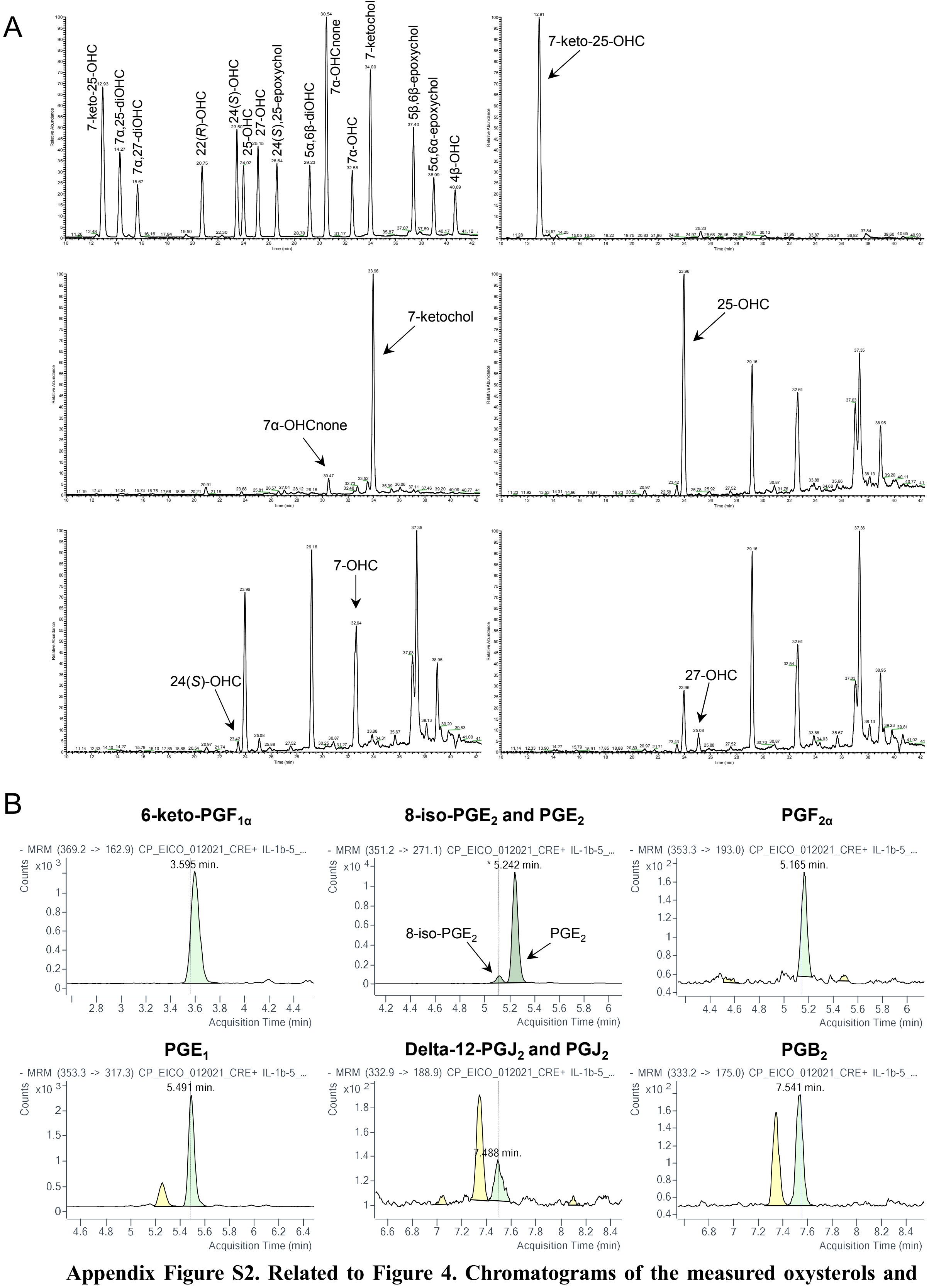

Appendix Figure S2. Related to Figure 4. Chromatograms of the measured oxysterols and eicosanoids. (A) Chromatograms of the measured oxysterols in pMBMEC supernatant isolated from a *Ch25h*$^{fl/fl}$+IL-1β mouse. Top left panel: equimolar (5 pmol.) mix of oxysterols, top right panel: Detection of 7-keto-25-OHC. Middle left panel: Detection of 7α-OHCnone and 7-ketochol. Middle right panel: Detection of 25-OHC. Bottom left panel: Detection of 24(*S*)-OHC and 7-OHC. Bottom right panel: Detection of 27-OHC. **(B)** HPLC-MS peaks of selected eicosanoids detected in pMBMECs supernatant isolated from *Ch25h*^{ECKO}+IL-1β mouse. Top left panel: Detection of 6-Keto-prostaglandin $F_{1α}$ (6-keto-$PGF_{1α}$), top middle panel: Detection of 8-isoprostaglandin E_2 and prostaglandin E_2 (8-iso-PGE_2 and PGE_2), top right panel: Detection of prostaglandin $F_{2α}$ ($PGF_{2α}$), bottom left panel: Detection of Prostaglandin E_1 (PGE_1), bottom middle panel: Detection of delta-12-Prostaglandin J_2 and prostaglandin J_2 (Delta-12-PGJ_2 and PGJ_2) and bottom right panel: detection of prostaglandin B_2 (PGB_2).

Appendix Figure S3

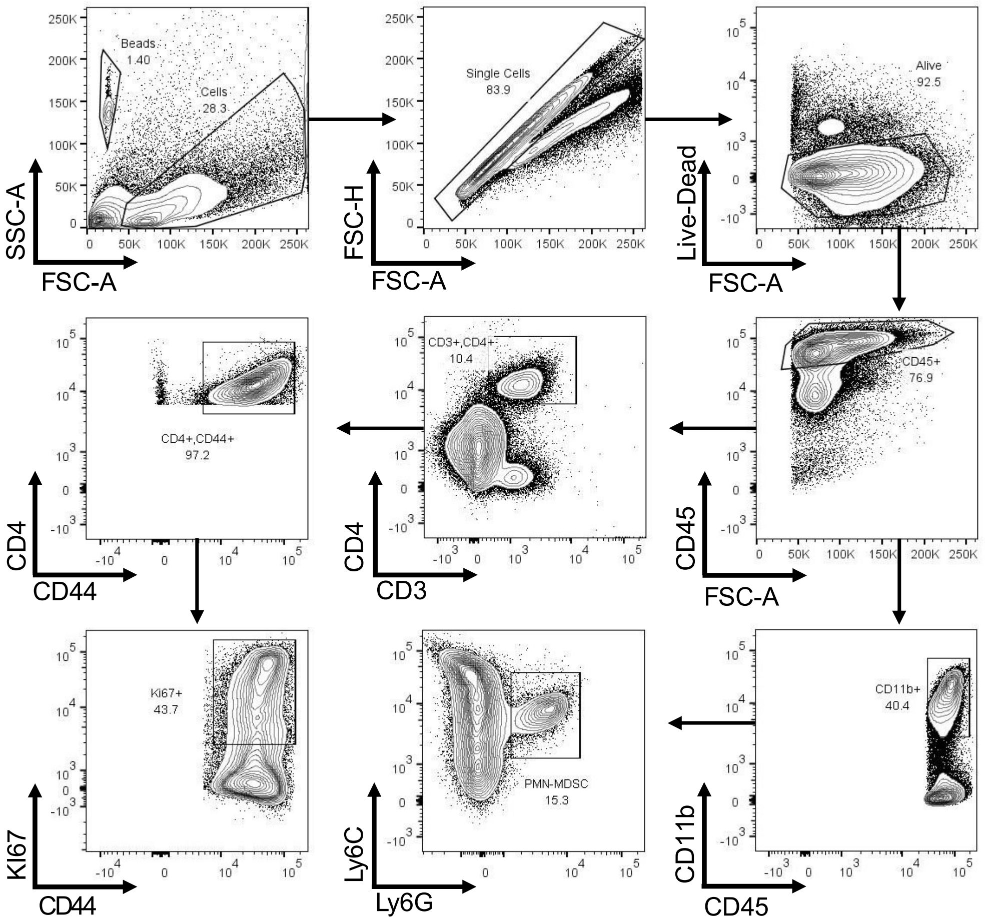

Appendix Figure S3. Related to Figure 5. Gating strategy for CNS infiltrating leukocytes Total cells are selected based on Forward Scatter (FSC-A) and side scatter plot (SSC-A). Doublet and dead cells are excluded. CD45^{+} cells are selected. Total CD11b^{+} are selected**.** PMN-MDSC are defined as Ly6C^{int}Ly6G^{+} Cells. CD4 T cells are selected based on CD4 and CD3 positivity. Memory CD4 T cells (CD44^{+}) are further selected**.** Ki67^{+} Cells are further selected.

Appendix Figure S4

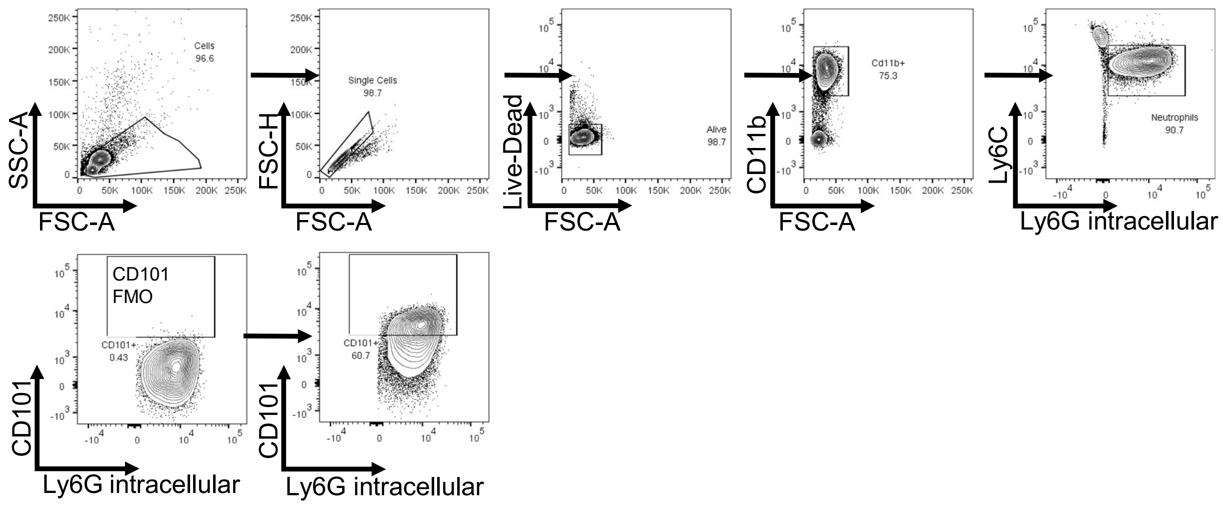

Appendix Figure S4. Related to Figure 6. Gating strategy for blood and CNS neutrophils. Gating strategy for analysis of blood neutrophils. Total Cells are selected based on Forward Scatter (FSC-A) and side scatter plot (SSC-A). Doublet and dead cells are excluded. $CD11b^+$ cells are selected. $Ly6C^{int}$Ly6G intracellular$^+$ cells are further selected. $CD101^+$ cells are defined base on a Fluorescence minus one (FMO).
