# Supplementary material for: Endothelial cell‐derived oxysterol ablation attenuates experimental autoimmune encephalomyelitis
Source: EMBO Rep. 2023 Jan 30;24(3):e55328. doi: 10.15252/embr.202255328 (PMC9986812; doi:10.15252/embr.202255328)
Supplement: Supplementary file 2 — Expanded View Figures PDF [file EMBR-24-e55328-s007.pdf]

# Expanded View Figures

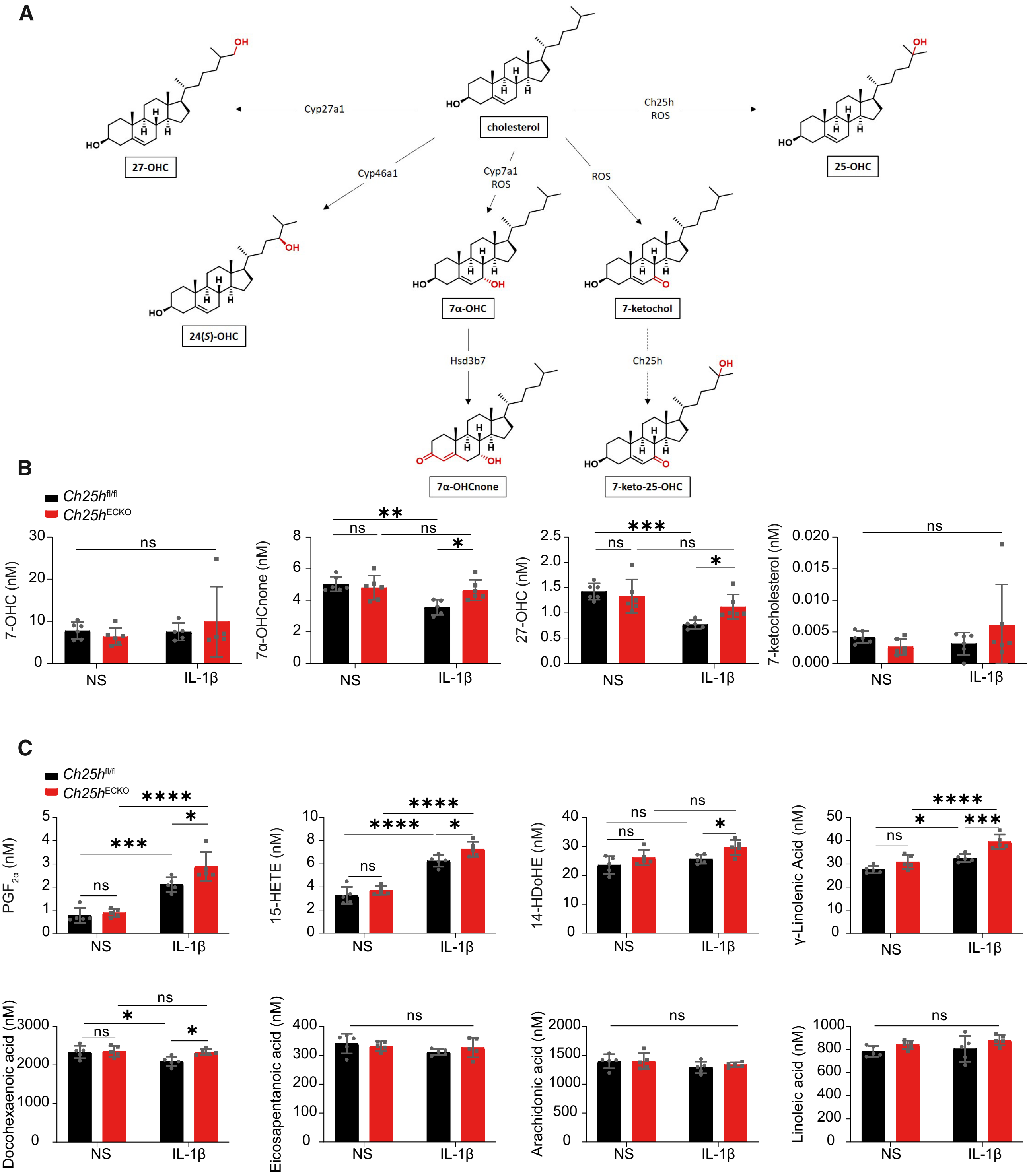


**Figure EV1.**

◀

**Figure EV1. Related to Fig 4 Oxysterol and eicosanoids levels.**

A Schematic representation of oxysterol metabolic pathways. Dashed arrow indicates a proposed pathway (as in Myers *et al*, 2013).

B Primary mouse brain microvascular endothelial cells (pMBMEC) were isolated from *Ch25h*$^{fl/fl}$ and *Ch25h*$^{fl/fl}$-Ve-CadherinCreERT2 mice (*Ch25h*$^{ECKO}$) injected with tamoxifen. Cells were left unstimulated (NS) or stimulated with IL-1β (10 ng/ml) during 24 h. Supernatant was then collected. Oxysterols were measured by HPLC-MS. 7-hydroxycholesterol (7-OHC), 7α-hydroxycholestenone (7α-OHCnone), 27-hydroxycholesterol (27-OHC), 7-ketocholesterol concentrations. $n = 6$ biological replicates/group except for *Ch25h*$^{fl/fl}$ IL-1β $n = 5$. Bars indicate mean ± SD.

C Same conditions as in (B), except that eicosanoids were measured by Liquid Chromatography-Mass Spectrometry. Prostaglandin $F_{2\alpha}$ ($PGF_{2\alpha}$), 15-Hydroxyeicosatetraenoic acid (15-HETE), 14-hydroxy-4Z,7Z,10Z,12 E,16Z,19Z-docosahexaenoic acid (14-HDoHE), γ-linolenic acid, Docohexanoic acid, Eicosapentanoic acid, Arachidonic acid, Linoleic acid concentrations. Bars indicates mean ± SD. $n = 5$ biological replicates/group.

Data information: ns, nonsignificant, *$P < 0.05$, **$P \leq 0.005$, ***$P \leq 0.0005$, ****$P \leq 0.00005$. *P*-values were determined by two-way ANOVA with Sidak's *post hoc* test.

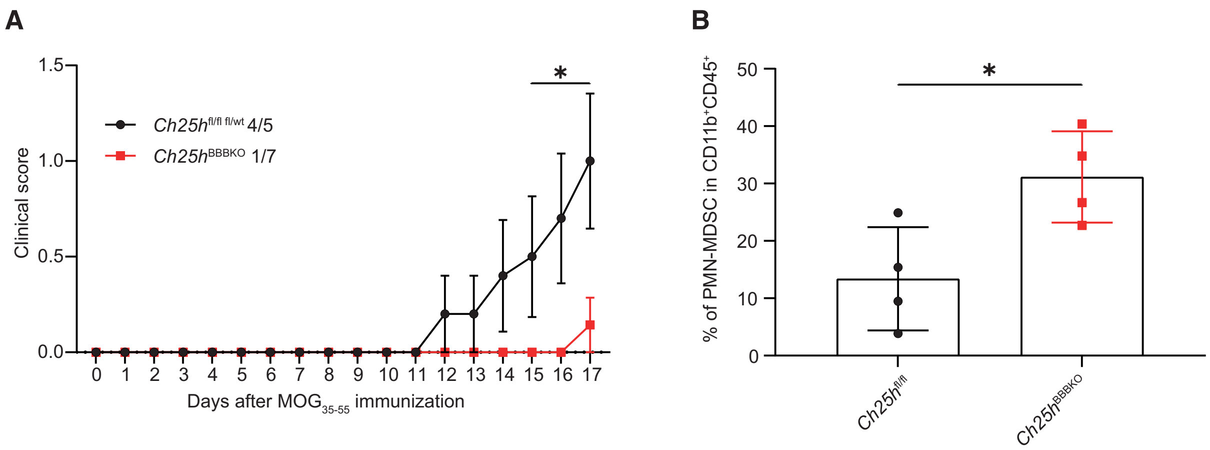


**Figure EV2. Related to Fig 5 CNS-specific *Ch25h* ECs deletion promotes CNS PMN-MDSC expansion.**

A EAE disease course in *Ch25h*$^{BBBKO}$ ($n = 7$ biological replicates) and Cre-negative littermates (*Ch25h*$^{fl/fl}$, $n = 5$ biological replicates). Bars indicate mean ± SEM.

B Percentage of CNS PMN-MDSC (live cells $CD45^+CD11b^+Ly6C^{int}Ly6G^+$) gated on $CD45^+CD11b^+$population assessed by flow cytometry in *Ch25h*$^{BBBKO}$ mice and *Ch25h*$^{fl/fl}$ at day 15 post-immunization ($n = 4$ biological replicates/group). Symbols depict individual mice and bars indicate mean ± SD.

Data information: *$P < 0.05$, ****$P < 0.00005$. *P*-values were determined by two-way ANOVA with Sidak's *post hoc* test (A) and by two-tailed unpaired *t*-test (B). The experiment was performed three times.

 © 2023 The Authors
