# Supplementary material for: Endothelial cell‐derived oxysterol ablation attenuates experimental autoimmune encephalomyelitis
Source: EMBO Rep. 2023 Jan 30;24(3):e55328. doi: 10.15252/embr.202255328 (PMC9986812; doi:10.15252/embr.202255328)
Supplement: Supplementary file 9 [file EMBR-24-e55328-s009.pdf]

EMBO reports

Endothelial cell-derived oxysterol ablation attenuates experimental autoimmune encephalomyelitis

Florian Ruiz, Benjamin Peter, Jessica Rebeaud, Solenne Vigne, Valentine Bressoud, Martin Roumain, Tania Wyss, Yannick Yersin, Ingrid Wagner, Mario Kreutzfeldt, Marisa Pimentel Mendes, Camille Kowalski, Gael Boivin, Léonard Roth, Markus Schwaninger, Doron Merkler, Giulio Muccioli, Stephanie Hugues, Tatiana Petrova, and Caroline Pot
DOI: 10.15252/embr.202255328

Corresponding author(s): Caroline Pot (Caroline.pot-kreis@chuv.ch) , Caroline Pot (Caroline.pot-kreis@chuv.ch)

Review Timeline:

Submission Date:	29th Apr 22
Editorial Decision:	8th Jun 22
Revision Received:	8th Nov 22
Editorial Decision:	8th Dec 22
Revision Received:	21st Dec 22
Accepted:	5th Jan 23

Editor: Esther Schnapp

Transaction Report:

(Note: With the exception of the correction of typographical or spelling errors that could be a source of ambiguity, letters and reports are not edited. Depending on transfer agreements, referee reports obtained elsewhere may or may not be included in this compilation. Referee reports are anonymous unless the Referee chooses to sign their reports.)

1st Editorial Decision 8th Jun 2022

Dear Prof. Pot,

Thank you for the submission of your research manuscript to our journal, which was now seen by three referees, whose reports are copied below.

We concur with the referees that the proposed role of endothelial-derived oxysterols downstream of Ch25h in regulation of myeloid-derived suppressor cells at the blood-brain barrier is in principle very interesting. However, referees also raise some concerns that need to be addressed to consider publication here.

I find the reports informed and constructive, and believe that addressing the concerns raised will significantly strengthen the manuscript. As the reports are below, and I think all points need to be addressed, I will not detail them here.

Given these positive recommendations, we would like to invite you to revise your manuscript with the understanding that the referee concerns (as in their reports) must be fully addressed and their suggestions taken on board. Please address all referee concerns in a complete point-by-point response. Acceptance of the manuscript will depend on a positive outcome of a second round of review. It is EMBO reports policy to allow a single round of revision only and acceptance or rejection of the manuscript will therefore depend on the completeness of your responses included in the next, final version of the manuscript.

We generally allow three months as standard revision time. As a matter of policy, competing manuscripts published during this period will not negatively impact on our assessment of the conceptual advance presented by your study. However, we request that you contact the editor as soon as possible upon publication of any related work, to discuss how to proceed. Should you foresee a problem in meeting this three-month deadline, please let us know in advance and we may be able to grant an extension.

*** Temporary update to EMBO Press scooping protection policy:
We are aware that many laboratories cannot function at full efficiency during the current COVID-19/SARS-CoV-2 pandemic and have therefore extended our 'scooping protection policy' to cover the period required for a full revision to address the experimental issues highlighted in the editorial decision letter. Please contact the scientific editor handling your manuscript to discuss a revision plan should you need additional time, and also if you see a paper with related content published elsewhere.***

IMPORTANT NOTE: we perform an initial quality control of all revised manuscripts before re-review. Your manuscript will FAIL this control and the handling will be DELAYED if the following APPLIES:
1. A data availability section providing access to data deposited in public databases is missing (where applicable).
2. Your manuscript contains statistics and error bars based on n=2. Please use scatter plots in these cases.

You can submit the revision either as a Scientific Report or as a Research Article. For Scientific Reports, the revised manuscript can contain up to 5 main figures and 5 Expanded View figures. If the revision leads to a manuscript with more than 5 main figures it will be published as a Research Article. In this case the Results and Discussion section should be separate. If a Scientific Report is submitted, these sections have to be combined. This will help to shorten the manuscript text by eliminating some redundancy that is inevitable when discussing the same experiments twice. In either case, all materials and methods should be included in the main manuscript file

Supplementary/additional data: The Expanded View format, which will be displayed in the main HTML of the paper in a collapsible format, has replaced the Supplementary information. You can submit up to 5 images as Expanded View. Please follow the nomenclature Figure EV1, Figure EV2 etc. The figure legend for these should be included in the main manuscript document file in a section called Expanded View Figure Legends after the main Figure Legends section. Additional Supplementary material should be supplied as a single pdf labeled Appendix. The Appendix includes a table of content on the first page with page numbers, all figures and their legends. Please follow the nomenclature Appendix Figure Sx throughout the text and also label the figures according to this nomenclature. For more details please refer to our guide to authors.

Please note that for all articles published beginning 1 July 2020, the EMBO Reports reference style will change to the Harvard style for all article types. Details and examples are provided at
https://www.embopress.org/page/journal/14693178/authorguide#referencesformat

When submitting your revised manuscript, please carefully review the instructions that follow below. Failure to include requested items will delay the evaluation of your revision.

1) a .docx formatted version of the manuscript text (including legends for main figures, EV figures and tables). Please make sure that the changes are highlighted to be clearly visible.

2) individual production quality figure files as .eps, .tif, .jpg (one file per figure).

3) a .docx formatted letter INCLUDING the reviewers' reports and your detailed point-by-point responses to their comments. As part of the EMBO Press transparent editorial process, the point-by-point response is part of the Review Process File (RPF), which will be published alongside your paper. For more details on our Transparent Editorial Process, please visit our website: https://www.embopress.org/page/journal/14693178/authorguide#transparentprocess
You are able to opt out of this by letting the editorial office know (emboreports@embo.org). If you do opt out, the Review Process File link will point to the following statement: "No Review Process File is available with this article, as the authors have chosen not to make the review process public in this case."

4) a complete author checklist, which you can download from our author guidelines (<http://embor.embopress.org/authorguide>). Please insert information in the checklist that is also reflected in the manuscript. The completed author checklist will also be part of the RPF.

5) Please note that all corresponding authors are required to supply an ORCID ID for their name upon submission of a revised manuscript (<https://orcid.org/>). Please find instructions on how to link your ORCID ID to your account in our manuscript tracking system in our Author guidelines (<http://embor.embopress.org/authorguide>).

6) We replaced Supplementary Information with Expanded View (EV) Figures and Tables that are collapsible/expandable online. A maximum of 5 EV Figures can be typeset. EV Figures should be cited as 'Figure EV1, Figure EV2" etc... in the text and their respective legends should be included in the main text after the legends of regular figures.

- For the figures that you do NOT wish to display as Expanded View figures, they should be bundled together with their legends in a single PDF file called *Appendix*, which should start with a short Table of Content. Appendix figures should be referred to in the main text as: "Appendix Figure S1, Appendix Figure S2" etc. See detailed instructions regarding expanded view here: <http://embor.embopress.org/authorguide#expandedview>.

- Additional Tables/Datasets should be labeled and referred to as Table EV1, Dataset EV1, etc. Legends have to be provided in a separate tab in case of .xls files. Alternatively, the legend can be supplied as a separate text file (README) and zipped together with the Table/Dataset file.

7) We would also encourage you to include the source data for figure panels that show essential data.

Numerical data should be provided as individual .xls or .csv files (including a tab describing the data). For blots or microscopy, uncropped images should be submitted (using a zip archive if multiple images need to be supplied for one panel). Additional information on source data and instruction on how to label the files are available <http://embor.embopress.org/authorguide#sourcedata>.

8) Our journal encourages inclusion of *data citations in the reference list* to directly cite datasets that were re-used and obtained from public databases. Data citations in the article text are distinct from normal bibliographical citations and should directly link to the database records from which the data can be accessed. In the main text, data citations are formatted as follows: "Data ref: Smith et al, 2001" or "Data ref: NCBI Sequence Read Archive PRJNA342805, 2017". In the Reference list, data citations must be labeled with "[DATASET]". A data reference must provide the database name, accession number/identifiers and a resolvable link to the landing page from which the data can be accessed at the end of the reference. Further instructions are available at <http://embor.embopress.org/authorguide#datacitation>.

9) Please make sure to include a Data Availability Section before submitting your revision - if it is not applicable, make a statement that no data were deposited in a public database. Primary datasets (and computer code, where appropriate) produced in this study need to be deposited in an appropriate public database (see <http://embor.embopress.org/authorguide#dataavailability>).

Please remember to provide a reviewer password if the datasets are not yet public.

The accession numbers and database should be listed in a formal "Data Availability " section (placed after Materials & Method) that follows the model below. Please note that the Data Availability Section is restricted to new primary data that are part of this study.

Data availability

The datasets (and computer code) produced in this study are available in the following databases:

- RNA-Seq data: Gene Expression Omnibus GSE46843 (https://www.ncbi.nlm.nih.gov/geo/query/acc.cgi?acc=GSE46843)
- [data type]: [name of the resource] [accession number/identifier/doi] ([URL or identifiers.org/DATABASE:ACCESSION])

*** Note - All links should resolve to a page where the data can be accessed. ***

10) Regarding data quantification, please ensure to specify the name of the statistical test used to generate error bars and P values, the number (n) of independent experiments underlying each data point (not replicate measures of one sample), and the test used to calculate p-values in each figure legend. Discussion of statistical methodology can be reported in the materials and methods section, but figure legends should contain a basic description of n, P and the test applied.
Please note that error bars and statistical comparisons may only be applied to data obtained from at least three independent biological replicates.
Please also include scale bars in all microscopy images.

We would also welcome the submission of cover suggestions, or motifs to be used by our Graphics Illustrator in designing a cover.

11) The journal requires a statement specifying whether or not authors have competing interests (defined as all potential or actual interests that could be perceived to influence the presentation or interpretation of an article). In case of competing interests, this must be specified in your disclosure statement. Further information: https://www.embopress.org/competing-interests

I look forward to seeing a revised version of your manuscript when it is ready. Please let me know if you have questions or comments regarding the revision.

Kind regards,

Deniz Senyilmaz Tiebe

Deniz Senyilmaz Tiebe, PhD
Editor
EMBO Reports

Referee #1:

TO THE AUTHORS
This certainly is a very interesting manuscript linking a mouse model of multiple sclerosis with Ch25h, 25-hydroxycholesterol and PGE2. I have a few suggestions the authors may like to consider.

Major Suggestions.
1. As this paper covers a rather wide scientific area, a scheme could be added showing the interactions of the different enzymes, metabolites, cells and immunological processes. Few readers will be expert in each of the different areas.
2. Being familiar with the work of Cyster on EAE and Ch25h I was forever wondering when it would be mentioned. I think some mention of these conflicting results could come in the Introduction.
3. It would be nice to have a better feel of how much oxysterol is actually being secreted by cells. Figure 3 has concentrations of eicosanoids in nM. Could the same units be used for oxysterols. In this regard it is also important to know the cell number. This is important as the concentration of 7-keto-25-hydroxycholesterol appears to be an order of magnitude greater than that of 25-hydroxycholesterol.
4. Following on from the last point, the authors present good evidence for Ch25h expression in BEC, but I wonder how important Ch25h expression in these cells is compared to macrophages expression. Both Diczfalusy and Bauman have shown activated macrophages generate high concentrations of 25-hydroxycholesterol in man and mouse. Again the use of concentration units would give the reader some idea.
5. With respect to experiments with IL-1β, Cyster and Russell have shown that 25-hydroxycholesterol attenuates IL-1β formation. This should be considered in the context of the experiments and their interpretation.

Other Suggestions.
1. Figure 3 and Experimental. More information should be given with respect to metabolites and cell cultures. The medium used and the cell number should be given.
2. Figure S2A and page 8. It is interesting that 24S-hydroxycholesterol is found in pMBMECs incubations. 24S-HC is thought to be a metabolite produced by Cyp46a1 in neurons. Similarly, 7α-hydroxycholestenone is generated by Cyp7a1 which is a hepatic enzyme. Some comment is needed.
3. Figure S2. Again it would be useful to have an idea of the concentrations of oxysterol so the reader can get a feel of the levels being discussed. Are these very minor oxysterols compared to 25-hydroxycholeasterol or more abundant? I would recommend a chromatogram being shown in the Supplemental.

4. Page 8. Could the authors provide a list of the 49 eicosanoids consistently detected above threshold. It would be nice if a chromatogram was included in the supplemental.
5. Page 9 and elsewhere. Perhaps some discussion of SREBP-1a is require. From the classic paper by Horton (PNAS 2003) it seems that γ-linolenic acid formation is regulated by this transcription factor.
6. Figure 4. What were the incubation concentrations of PGE2 and 25-OHC?
7 Experimental. It would be good to indicate the deuterated standards used for quantification of metabolites.
8. Oxysterol measurements. What was the volume of cell supernatant added to extraction solvent?

In summary a very interesting paper, but could be improved by providing the reader with a little more quantitative and experimental data.

Referee #2:

The manuscript by Ruiz et al explores the role of the enzyme Ch25h and its downstream metabolites in brain endothelial cells (ECs) during neuroinflammation. The authors have previously demonstrated that Ch25h full knockout mice develop less severe EAE, a finding that was then linked to Ch25h expression in hematopoietic cells (Chalmin et al 2015). In the current manuscript the authors explore the role of Ch25h in endothelial cells, first demonstrating that Ch25h expression is upregulated in brain ECs during EAE. Using several mouse strains to remove Ch25h expression from ECs (including BBB ECs), the authors demonstrate a partial protection from developing EAE in the absence of Ch25h. This is the major finding of the manuscript and represents a novel and interesting discovery. The authors then go on to explore the transcriptional and lipidomic profile of Ch25h deficient ECs in the presence or absence of IL1b. Finally the authors attempt to link the diminished EAE severity observed in Ch25h knockout mice with an accumulation of suppressive neutrophils in the CNS. The neutrophil-related findings are potentially interesting but would have to be addressed by additional experiments in order to better link the attenuated EAE with the accumulation of neutrophils in the CNS.

Major points:

1. The results in Figure 1 would be considerably strengthened by showing Ch25h-eGFP expression in tissue sections. The authors claim that increased expression during EAE is restricted to the EC compartment, however the comparison is made with CNS resident and CNS hematopoietic cells, which each represent pools of several different cell types.

2. Figure 4C: The suppressive capacity of the identified CNS-infiltrating neutrophils relies entirely on this figure. However, it lacks a proper control. Simply showing that the addition of CNS-derived neutrophils limits proliferation of anti-CD3/CD28-stimulated T-cells could have multiple explanations. If the authors claim that PMN-MDSCs isolated from the EAE CNS are indeed suppressive they should compare them to "naive" neutrophils isolated for example from blood or bone marrow of a naive mouse. If authors think the suppression is mediated by soluble factors, an alternative approach could be to use conditioned media.

3. The authors need to better link the accumulation of neutrophils in the CNS with the abrogation of disease. Depletion of circulating neutrophils appears to, counterintuitively, cause accumulation of neutrophils in the CNS. Could the authors instead attempt to boost accumulation of suppressive neutrophils using for example G-CSF or PGE2 administration (or other means)?

Minor points:

1. It would be interesting to understand where the neutrophils accumulate inside the CNS. Are they mainly located in subarachnoid/meningeal/perivascular spaces or do they infiltrate the brain parenchyma?

2. The authors should report counts instead of % of live cells as the latter can be misleading. See Fig 4f, 6d-f.

3. The GBM data is interesting but is mostly correlative and not backed up by any experimental data in for example a mouse model. Taking this into account, it is my opinon tha the authors should revise their discussion on the GBM data accordingly.

4. For figure 3 it would be helpful to guide the reader by including a schematic figure of cholesterol metabolism including enzymes and metabolite steps.

Referee #3:

The manuscript by Florian Ruiz et al. "Endothelial-derived oxysterols regulate myeloid-derived suppressor cells at the blood-brain barrier" describes a new role for the enzyme Ch25h and its product 25-OHC in EAE, namely the inhibition of myeloid suppressor cell expansion in the inflammatory process at the blood brain barrier. Using a new mouse strain, made in the lab, which reports expression of Ch25h and which can be used for tissue-specific deletion of the enzyme, they show that endothelial cells of the CNS are the main cell type expressing Ch25h in EAE and that its deletion in endothelial cells and importantly in CNS-ECs leads to a delayed and lowered EAE, similar to mice lacking CH25h body-wide as shown in a previous paper by the lab. From that point they show that the lack of Ch25h in inflamed endothelial cells changes expression of lipid biosynthetic pathways leading finally to a shift from 25-OHC to PGE2 in mice lacking Ch25h in ECs. In vitro data shows that PGE2 promotes differentiation of MDSCs whereas 25-OHC strongly inhibits their differentiation. These findings could explain the found expansion of MDSCs in the CNS of CH25hEC-KO mice with EAE compared to control mice. Finally, depletion of mature neutrophils at DPI 14 led to a near complete resistance of EAE in CH25hEC-KO mice and a further expansion of MDSCs in the CNS. Additionally, one figure shows analyses of data from human glioblastoma ECs in context to Ch25h expression and its similarity to ECs of mice lacking Ch25h in EAE CNS.

This manuscript is very interesting, well written and the experiments are thoroughly performed. Nevertheless, some points are not clear enough yet for the main claim to be made and further experiments should be performed to make the main conclusions more solid.

1. The main claim of the paper is that EAE is lower in CH25hEC-KO due to expansion of MDSCs in the CNS. Several points are unclear here.
a) Expansion: in all experiments showing MDSCs in the CNS only percentages of live cells are shown. Since EAE is lower in CH25hEC-KO mice, also infiltration of T cells and of monocytes should be reduced. Hence, the higher percentage of MDSCs in the CNS in EAE in these mice might only be relative. Therefore, it is necessary to determine the absolute cell numbers and to show them next to the percentages. This accounts also for data showing T cell-infiltrates.
b) MDSCs in the CNS: As also discussed by the authors, it is difficult to distinguish PMN-MDSCs from normal PMNs in the mouse and an alternative scenario of the effectiveness of the PMN depletion might be their pathogenic effect in EAE as claimed by several others previously combined with a lack of depletion of PMNs in the CNS. To be sure that the CNS PMNs are indeed MDSCs, one should perform an Arginase 1 staining (e.g. intracellular with clone A1exf5) and compare this to PMNs in the blood, spleen and BM.
c) How do 25-OHC/PGE2 act on immature PMN? Unfortunately, PMN/ MDSCs of controls and CH25hEC-KO were only compared in the CNS and not in blood, spleen and BM (eg. Figure 4f). This should at least clarify whether 25-OHC/PGE2 have a more systemic or local impact at the CNS.
d) Are MDSCs also expanded in the CNS of CH25h-BBBKO mice? Since CH25hEC-KO mice may show an activation of ECs in the whole body and an increased level of PGE2 over 25-OHC, it might be possible that the increase of PMN/MDSCs occurs already at the level of the BM and due to an expansion there, more of these cells may migrate to the CNS during inflammation. Therefore, it is also important to know whether also CH25h-BBBKO mice, which show the same EAE phenotype, also show an enhanced level of PMN/MDSCs in the CNS.

2. Since EAE is mediated by T helper cells, an analysis of CNS-infiltrating T cells is lacking in respect to Th17/Th1/ThGMCSF/Tregs. These should also be analyzed in spleen/dLNs and blood. All in % and in absolute numbers.

3. Ch25h is important in generating the EBI2 ligand 7a,25-OHC. This ligand was previously shown to be upregulated in EAE in the CNS. Since your data show that EC are the main cells expressing Ch25h in the CNS in EAE one would expect a lack of 7a,25-OHC upregulation in the CNS of CH25h-BBBKO mice. Showing this would make your findings using the reporter mouse even more convincing. In the same line a western blot against CH25h of full CNS vs. CNS-ECs of healthy vs. EAE of control vs. CH25hEC-KO would be a great confirmation of your reporter data.

4. Ch25h expression in EAE: Microglia cells should be properly stained with CD11b and CD45 and then gated on CD11b (int) and CD45(int) for analysis of reporter expression. Ter119 staining to exclude erythrocytes should be performed on a separate channel. In addition, the FACS staining should also be shown reversed by gating on all live cells expressing Ch25h-eGFP and then checking in which population these positive cells fall. By such a gating a staining of CNS from CH25hEC-KO mice should reveal a near complete absence of eGFP in the live cell population. Since FACS shows only a part of CNS resident cells, histology against eGFP in Ch25h reporter mice with EAE should reveal a more complete picture of Ch25h expression in the CNS in EAE.

Minor Points:
5. Although very interesting, the data shown on GBM ECs is rather confounding the overall story and not necessary for the main points to make. I would suggest to omit this and to put this rather in another small publication.
6. Some histology of infiltration and demyelination of controls versus CH25hEC-KO in EAE would be nice to have to see whether there might be also other qualitative changes.
7. For EAE experiments it would be nice to see for each group shown the exact N number of the mice directly in the figure.
8. Mean max score: Was this calculated only of diseased mice?

9. Figure 2e seems to show the averaged data of 3 measurements for each condition. I would suggest to show them individually.
10. Figure 4c shows that PMN-MDSCs can inhibit T cell proliferation. A control showing the specificity of these cells by using PMN from BM would strengthen that point.
11. Correlation should be rather -0.68, what do the ** mean here?
12. Figure 6a: There seems to be at least some impact of the combo treatment also in controls.
13. In Figure 6e and f the comparison of controls vs. CH25hEC-KO in EAE should also be shown.
14. For Figure 6f one should rather show bars than lines since the loss of PMNs occurs most likely only on day 14 and not before.
15. Figure 6f right: axes labels were forgotten
16. Page 16 mentions deletion of Ch25h in GBM ECs although it is rather likely "not expressed" what was meant.
17. Although in general well written the grammar needs to be checked.

POINT-BY-POINT RESPONSE TO REVIEWERS COMMENTS

Referee #1:

Major Suggestions

1. As this paper covers a rather wide scientific area, a scheme could be added showing the interactions of the different enzymes, metabolites, cells and immunological processes. Few readers will be expert in each of the different areas.

We are now providing a graphical abstract that summarizes our model and is included as a synopsis.

2. Being familiar with the work of Cyster on EAE and Ch25h I was forever wondering when it would be mentioned. I think some mention of these conflicting results could come in the Introduction.

We are now mentioning these contrasting results in the introduction on page 3 – Lines: 71-75.

3. It would be nice to have a better feel of how much oxysterol is actually being secreted by cells. Figure 3 has concentrations of eicosanoids in nM. Could the same units be used for oxysterols. In this regard it is also important to know the cell number. This is important as the concentration of 7-keto-25-hydroxycholesterol appears to be an order of magnitude greater than that of 25-hydroxycholesterol.

We now show all the oxysterols, including 25-hydroxycholesterol, in concentration in nM (rebuttal Figure 1, new Figure 3 and new Figure EV1 related to Figure 3) according to the referee's comment. Concerning the comparison of 7-keto-25-hydroxycholesterol with 25-hydroxycholesterol, we could previously not perform a direct comparison between these two oxysterols when represented in the form of a *ratio oxysterol/ d-oxysterol*. Indeed the intensity of the peaks measured by HPLC-MS is not necessarily directly proportional to the compound concentration as it also depends on the intrinsic ionizability of the molecule. We are now showing the concentrations of the oxysterols that enables us a direct comparison. The level of 25-hydroxycholesterol is in the same range as of the levels of 7-keto 25-hydroxycholesterol with 25-OHC being however at a higher concentration in all conditions. We have now included those new results in the new manuscript (new Figure 3 and new Figure EV1 related to Figure 3).

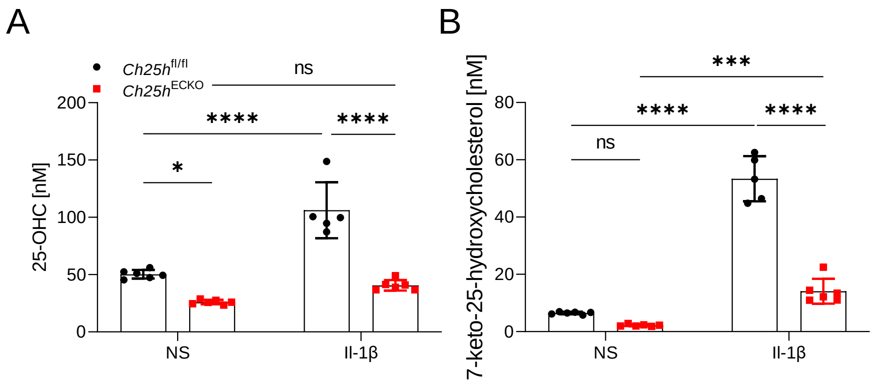

Rebuttal Figure 1. 25-OHC and 7-keto-25-hydroxycholesterol nanomolar concentration measured by HPLC-MS in the supernatant of pMBMECs isolated from *Ch25h*$^{fl/fl}$ mice and *Ch25h*^{ECKO} mice. Cells were left unstimulated (NS) or stimulated by IL-1β. N = 6 biological replicates/group except for Ch25$^{fl/fl}$ IL-1β were n = 5 biological replicates. ns= non-significant, * P < 0.05, ** P ≤ 0.005, **** P ≤ 0.00005. p values were determined by two-way ANOVA with Sidak's post hoc test.

Concerning the question of the cell number, pMBMECs are cultivated in 96 well plates and plated at a concentration of 51 000 digested capillaries/cm^2 onto matrigel-coated wells. The purpose of the digestion step of the vascular pellet prior to pMBMECs seeding is not to obtain single cells but capillary fragment, which is the approach that gives the best results for microvascular ECs culture. Therefore, the seeding density is not based on the number of single cells but on the number of capillary fragments. Oxysterol measurements were performed when cells were confluent to avoid a potential proliferation bias induced by IL-1β stimulation. Furthermore IL-1β is only added in the culture when cells are confluent for 24hours. Thus, the cell count was not performed at the time of supernatant collection.

4. Following on from the last point, the authors present good evidence for Ch25h expression in BEC, but I wonder how important Ch25h expression in these cells is compared to macrophages expression. Both Diczfalusy and Bauman have shown activated macrophages generate high concentrations of 25-hydroxycholesterol in man and mouse. Again the use of concentration units would give the reader some idea.

We agree that 25-OHC concentration is an important determinant of its biological activity and we have adapted the manuscript accordingly. Please also see point 3 above and referee 2 major point 1. Comparison of 25-hydroxycholesterol *in vitro* in two different subsets of primary cells might not reflect the *in vivo* biological effect. In order to answer this question and question 1 of referee 2, we have now performed additional experiments and quantified Ch25h expression by RNA scope in phagocytes (macrophages/microglia) and endothelial cells (ECs) in the CNS of non-immunized and EAE mice (rebuttal Figure 3 below and new Figure 1). We found that Ch25h was significantly more increased in ECs compared to phagocytes (macrophages/microglia) in the CNS both in non-immunized and EAE conditions. Despite increased expression of Ch25h in both phagocytes and ECs during EAE, our results show that the sole deletion of Ch25h in CNS ECs is sufficient to attenuate EAE.

5. With respect to experiments with IL-1β, Cyster and Russell have shown that 25-hydroxycholesterol attenuates IL-1β formation. This should be considered in the context of the experiments and their interpretation.

We agree that activated innate immune cells are a major source of IL-1β and within this context; this has to be taken in consideration. However, all the IL-1β-stimulation experiments were performed on pMBMECs and our RNAseq analysis indicates either low or absence of IL-1β mRNA in these cells. In light with this, IL-1β was not identified as a differentially expressed gene in *Ch25h*^{ECKO} versus *Ch25h*$^{fl/fl}$ pMBMECs. This suggests that IL-1β is either expressed at very low levels or not expressed by brain endothelial cells. We also refer the referee to the vascular single cell database from the mouse brain from Christer Betsholtz laboratory (http://betsholtzlab.org/VascularSingleCells/database.html) where it can be seen that IL-1β transcripts are only detectable in Microglia at steady state. Similarly, the RNAseq analysis of CNS ECs published by Munji et al shows that IL-1β mRNA expression in CNS ECs is very low both at steady state and during EAE (Munji *et al*, 2019). Therefore, we consider that a bias of the interpretation of our results, resulting from increased production of IL-1β by CNS ECs in absence of 25-OHC, is unlikely.

Other Suggestions

1. Figure 3 and Experimental. More information should be given with respect to metabolites and cell cultures. The medium used and the cell number should be given.

The medium used in culture is now included in the Material and Method section: isolation and culture of primary brain microvascular cells, page 20 - lines: 588-592. Regarding the cell number please refer to the rebuttal response of major point 3 above.

2. Figure S2A and page 8. It is interesting that 24S-hydroxycholesterol is found in pMBMECs incubations. 24S-HC is thought to be a metabolite produced by Cyp46a1 in neurons. Similarly, 7α-hydroxycholestenone is generated by Cyp7a1, which is a hepatic enzyme. Some comment is needed.

Concerning the 24*(S)*-hydroxycholesterol in the pMBMECs supernatant, its concentration is 10 times lower than the levels of 25-hydroxycholesterol and 7-keto-25-hydroxycholesterol. We did not detect Cyp46a1 in our pMBMECs on the RNAseq data. We further performed RT-qPCR on pMBMECs under all conditions (*Ch25h*^{ECKO} and *Ch25h*$^{fl/fl}$ pMBMECs treated or not with IL-1β) and confirmed the absence of Cyp46a1 mRNA detection (Rebuttal Fig 2a). We propose that Cyp46a1 is not expressed or at undetectable levels in our setting. Cyp46a1 has however been described in endothelial cells in the retina (Saadane *et al*, 2019) and in a protein atlas (https://www.proteinatlas.org/ENSG00000036530-CYP46A1/single+cell+type). Furthermore, it is not excluded that 24*(S)*-hydroxycholesterol is present in the Fetal Bovine Serum used in pMBMECs culture media. Its modest and just significant (p.value = 0.0281) increase upon IL-1β stimulation in *Ch25h*^{ECKO} pMBMECs could be an indirect results of a reduced Cyp39a1 activity, the enzyme that catalyzes its hydroxylation in 7α,24(S)-OHC. However, in our RNAseq, Cyp39a1 mRNA expression was increased just reaching significance upon IL-1β stimulation (adjusted p.value = 0.053 and 0.045, FC 1.29 and 1.3 for *Ch25h*^{ECKO} and *Ch25h*$^{fl/fl}$ pMBMECs respectively) but did not statistically differ between *Ch25h*^{ECKO} and *Ch25h*$^{fl/fl}$ pMBEMCs. However, Cyp39a1 mRNA expression is not necessarily correlated with its activity at this specific time point (24 hours of IL-1β stimulation). 24S-HC and concentration measurement in cell-free culture medium and in Cy46a1 K.O pMBMECs-derived supernatant might help address this specific point, however, we consider that this out of the scope of our manuscript.

The enzyme responsible for 7α-hydroxycholestenone synthesis is HSD3B7 that synthesizes 7α-hydroxycholestenone from 7α-OHC. 7α-OHC is produced by Cyp7a1 or in a ROS dependent manner. It could also be present in Fetal Bovine Serum used in pMBMECs culture media. We therefore performed RT-qPCR on *Ch25h*$^{fl/fl}$ and *Ch25h*^{ECKO} pMBMECs and found that Cyp7a1 is expressed by pMBMECs at low levels compared to the liver control and its expression is increased upon IL-1β stimulation in *Ch25h*$^{fl/fl}$ pMBMECs (Rebuttal Fig 2b). This contrasts with the results obtained in the supernatant showing that 7α-OHC levels are not impacted by the deletion or IL-1β stimulation but does suggest that Cyp7a1 expression is not restricted to the liver. In light with this, 7α-hydroxycholestenone reduction observed in *Ch25h*$^{fl/fl}$ pMBMECs supernatant could be due to a reduction of HSD3B7 activity induced by IL-1β stimulation. Furthermore, we did not detected significant changes in HDS3B7 mRNA levels in our RNAseq when comparing either *Ch25h*$^{fl/fl}$ NS and *Ch25h*$^{fl/fl}$ IL-1β (adjusted p.value = 0.33, Table S2) or *Ch25h*$^{fl/fl}$ IL-1β and *Ch25h*^{ECKO} IL-1β (adjusted p.value = 0.7, Table S3).

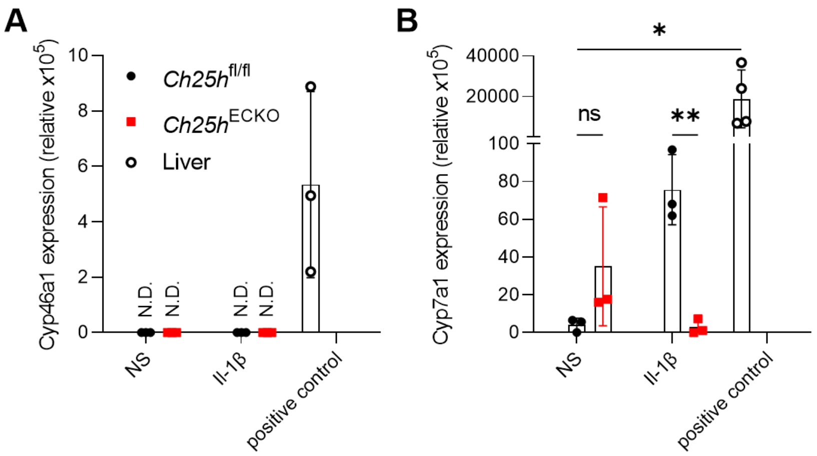

Rebuttal Figure 2. Cyp46a1 and Cyp7a1 expressions by pMBMECs. RNA isolated from confluent pMBMECs non-stimulated (NS) or stimulated by IL-1β (10ng/ml, 24 hours) was subjected to quantitative real-time PCR (RT-PCR) relative to the expression of mRNA encoding GAPDH ($2^{-\Delta CT}$ x100000) to examine expression of (a) Cyp46a1 and (b) Cyp7a1. Liver was used as a positive control for mRNA expression. N=3 biological replicates/group. ns= non-significant, * $P < 0.05$, ** $P \leq 0.005$, **** $P \leq 0.00005$. p values were determined by two-way ANOVA with Sidak's post hoc test.

3. Figure S2. Again it would be useful to have an idea of the concentrations of oxysterol so the reader can get a feel of the levels being discussed. Are these very minor oxysterols compared to 25-hydroxycholesterol or more abundant? I would recommend a chromatogram being shown in the Supplemental.

We now show all oxysterols with their concentrations in the revised manuscript and Figures (rebuttal Figure 1, new Figure 3 and new Figure EV1 related to Figure 3). This now enables a direct comparison of oxysterol concentrations in the pMBMECs supernatants. 25-hydroxycholesterol and 7-keto-25-hydroxycholesterol are the most abundant oxysterols measured. For example, the concentration of 24-(*S*)OHC and 27-OHC are ten times lower than the one of 25-hydroxycholesterol and 7-keto-25-hydroxycholesterol *in-vitro*. This is in clear contrast with the levels of 24*(S)*-OHC and 25-OHC in the total spinal cord (new Figure 3D) showing that, as previously described in many studies, 24*(S)*-OHC is the most abundant oxysterol. This further emphasize our hypothesis that local concentrations of 25-OHC at the level of the microvascular endothelium is different from the concentrations at the tissue scale. We changed the text of the manuscript accordingly.

The chromatograms are shown below in Rebuttal Figure 3. We did not include them in the manuscript as discussed above (Referee 1, question 3) because the intensity of the peaks measured by HPLC-MS is not necessarily directly proportional to the compound concentration as it depends on the intrinsic ionizability of the molecule. We however now show the concentrations for all the measured oxysterols.

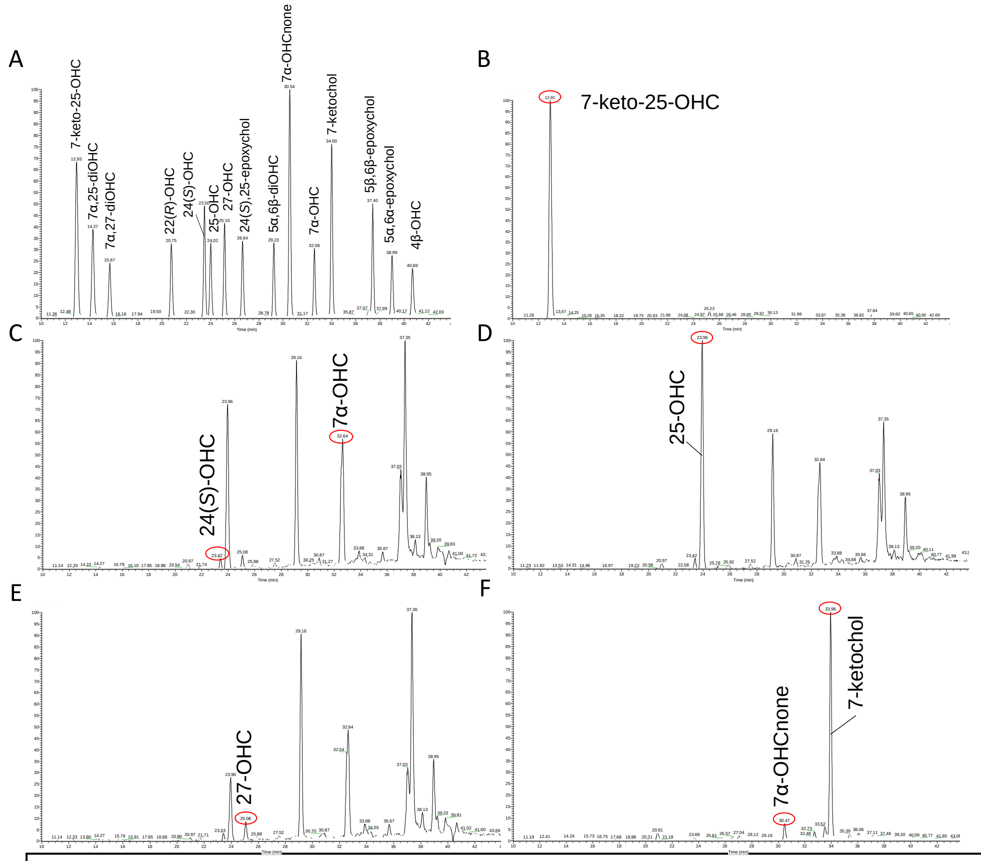

Rebuttal Figure 3: Chromatograms of the measured oxysterols. A) Equimolar mix of oxysterols, 5 pmol., B) Detection of 7-keto-25-OHC in pMBMEC supernatant isolated from a *Ch25h*$^{fl/fl}$+IL-1β mouse. C) Detection of 24*(S)*-OHC and 7α-OHC in pMBMEC supernatant isolated from a *Ch25h*$^{fl/fl}$+IL-1β mouse. D) Detection of 25-OHC in pMBMEC supernatant isolated from a *Ch25h*$^{fl/fl}$+IL-1β mouse. E) Detection of 27-OHC in pMBMEC supernatant isolated from a *Ch25h*$^{fl/fl}$+IL-1β mouse. F) Detection of 7α-OHCnone and 7-ketochol in pMBMEC supernatant isolated from a *Ch25h*$^{fl/fl}$+IL-1β mouse.

4. Page 8. Could the authors provide a list of the 49 eicosanoids consistently detected above threshold. It would be nice if a chromatogram was included in the supplemental.

Please find the list of the eicosanoids that were detected above threshold:

6-keto-PGF1a, 5-iPF2a-VI, 8-iso-PGE2, PGF2a, PGE2, PGE1, Delta 12-PGJ2, PGJ2, PGB2, 17,18-DiHETE, 5,15-DiHETE, 14,15-DiHETE, 12,13-DiHOME, 9,10-DiHOME, 14,15-DiHETrE, 12(S)-HHTrE, 19,20-DiHDPA, 11,12-DiHETrE, EKODE, 15-deoxy-PGJ2, 15-HEPE, 20-HETE, 12-HEPE, 5,6-DiHETrE, 13-HODE, 9-HODE, 13-KODE, 15-HETE, 17-HDoHE, 15-KETE, 9-KODE, 11-HETE, 14-HDoHE, 12-HETE, 9-HETE, 11-HDoHE, 15(S)-HETrE, 5-HETE, 12(13)-EpOME, 9(10)-EpOME, 4-HDoHE, 16(17)-EpDPE, 11-HEDE, 5-HETrE, Eicosapentanoic acid, Alpha-LinolenicAcid, Gamma-LinolenicAcid, docohexaenoic acid, arachidonic acid, Linoleic acid.

Rebuttal Figure 4 is showing the chromatograms of the eicosanoids displayed in the manuscript. As the intensity of the peaks measured by HPLC-MS it is not directly proportional to the compound concentration, we prefer to show only the concentrations of the eicosanoids in the manuscript.

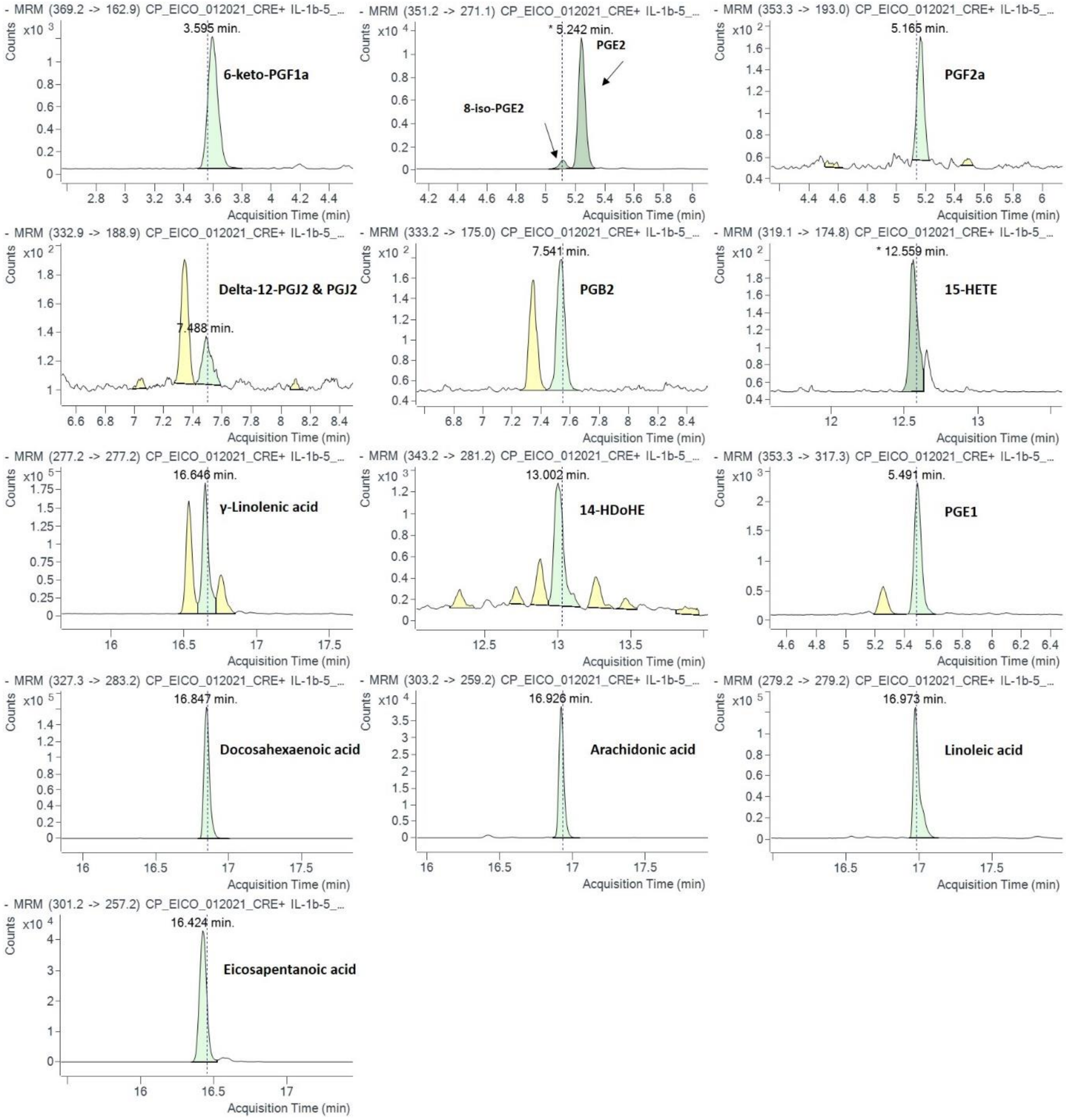

Rebuttal Figure 4. HPLC-MS peaks of selected eicosanoids detected in pMBMECs supernatant as in Rebuttal Figure 3.

5. Page 9 and elsewhere. Perhaps some discussion of SREBP-1a is require. From the classic paper by (Horton (PNAS 2003) it seems that γ-linolenic acid formation is regulated by this transcription factor.

We have now added a discussion about the potential involvement of SREBP-1a in increased production of *γ*-linolenic acid and FADS2 synthesis in absence of 25-OHC on pages 16 – lines: 457-460.

6. Figure 4. What were the incubation concentrations of PGE2 and 25-OHC?

The concentration of PGE_2 and 25-OHC were 20nM and 1µM respectively. They are provided in the Material and Methods section of the manuscript in the "Bone marrow derived cell culture and MDSC polarization" at page 23- lines: 692-693. We have now added the concentration in the Figure 4 legend.

7 Experimental. It would be good to indicate the deuterated standards used for quantification of metabolites.

We used d7-4β-OHC and d7-24(R/S)-OHC. Method is published (Mutemberezi *et al*, 2016) and cited in the Material and Methods section page 21 – lines: 630-631.

8. Oxysterol measurements. What was the volume of cell supernatant added to extraction solvent?

The volume of the supernatant for oxysterol measurements was 200µL, it was added to the extraction solvent (8ml dichloromethane, 4 ml methanol with BHT and 2ml of water with EDTA). We have now included this information in Material and Methods section page 21 – lines: 630.

In summary a very interesting paper, but could be improved by providing the reader with a little more quantitative and experimental data. This certainly is a very interesting manuscript linking a mouse model of multiple sclerosis with Ch25h, 25-hydroxycholesterol and PGE2. I have a few suggestions the authors may like to consider.

We thank the referee for his suggestions, we believe that with the proposed modifications and addition of new results in the manuscript and Figures according to his comments have strengthened our manuscript.

Referee #2:

The manuscript by Ruiz et al explores the role of the enzyme Ch25h and its downstream metabolites in brain endothelial cells (ECs) during neuroinflammation. The authors have previously demonstrated that Ch25h full knockout mice develop less severe EAE, a finding that was then linked to Ch25h expression in hematopoietic cells (Chalmin et al 2015). In the current manuscript the authors explore the role of Ch25h in endothelial cells, first demonstrating that Ch25h expression is upregulated in brain ECs during EAE. Using several mouse strains to remove Ch25h expression from ECs (including BBB ECs), the authors demonstrate a partial protection from developing EAE in the absence of Ch25h. This is the major finding of the manuscript and represents a novel and interesting discovery. The authors then go on to explore the transcriptional and lipidomic profile of Ch25h deficient ECs in the presence or absence of IL1b. Finally the authors attempt to link the diminished EAE severity observed in Ch25h knockout mice with an accumulation of suppressive neutrophils in the CNS. The neutrophil-related findings are potentially interesting but would have to be addressed by additional experiments in order to better link the attenuated EAE with the accumulation of neutrophils in the CNS.

Major points:

1. The results in Figure 1 would be considerably strengthened by showing Ch25h-eGFP expression in tissue sections. The authors claim that increased expression during EAE is restricted to the EC compartment, however the comparison is made with CNS resident and CNS hematopoietic cells, which each represent pools of several different cell types.

We thank the referee for this important comment. We initially focused on ECs and only evaluated Ch25h-eGFP expression by flow cytometry on CNS $CD45^+$ cells on a bulk cellular extraction dedicated to ECs and not on leukocyte extraction. Those results suggested that Ch25h-eGFP expression was restricted to ECs. We now properly evaluated Ch25h-eGFP expression on dedicated leukocyte extraction by flow cytometry and on tissue sections. We show that microglia also express Ch25h-eGFP as suggested by the referee. Flow cytometry results are discussed in detail on referee 3 major point 4.

We were not able to visualize eGFP expression in CNS tissue section, as eGFP expression is sensitive to fixation. In line with this observation, Ch25h-eGFP was only detectable in flow cytometry analysis in unfixed cells that were acquired immediately after staining which might explain that eGFP expression is below detection threshold in fixed tissues. To overcome this, we performed RNA *in-situ* hybridization of Ch25h in CNS tissue sections, comparing non immunized mice and mice at the peak of EAE (Score 2,5-3). Isolectin B4 and IBA1 were used to identify ECs and activated macrophages (phagocytes)/microglia respectively. We used IBA1 as increased Ch25h expression in microglia had been described in EAE (Wanke *et al*, 2017). We identified Ch25h expression in ECs and macrophages/microglia; its expression increased during EAE in both cell types (Rebuttal Fig. 5a and new Fig. 1A). Ch25h expression was significantly higher in ECs both at steady state and during EAE (Rebuttal Fig. 5b and new Fig. 1B). These results, together with the attenuated EAE observed in *Ch25h*^{ECKO}, *Ch25h*^{BBBKO} and *Ch25h*^{BECKO} confirm the importance of endothelial cells as a source of *Ch25h* and nuances the previous studies suggesting that activated myeloid cells are the main source of 25-OHC in inflamed tissue. Please also refer to the responses of Referee 3 major points 3 and 4 and new Fig. 3 D. However, we do not rule out that Ch25h deletion in macrophages/microglia can modulate EAE but this was not the scope of our manuscript.

These new results unambiguously demonstrate that Ch25h expression is not restricted to the EC compartment. We have now adapted the manuscript accordingly. The results of the RNA *in-situ* hybridization are now shown in new Fig. 1 A and B of the manuscript and the discussion have been adapted in accordance to the new results. Despite increased expression of Ch25h in both phagocytes and ECs during EAE, our results show that the sole deletion of Ch25h in CNS ECs is sufficient to attenuate EAE.

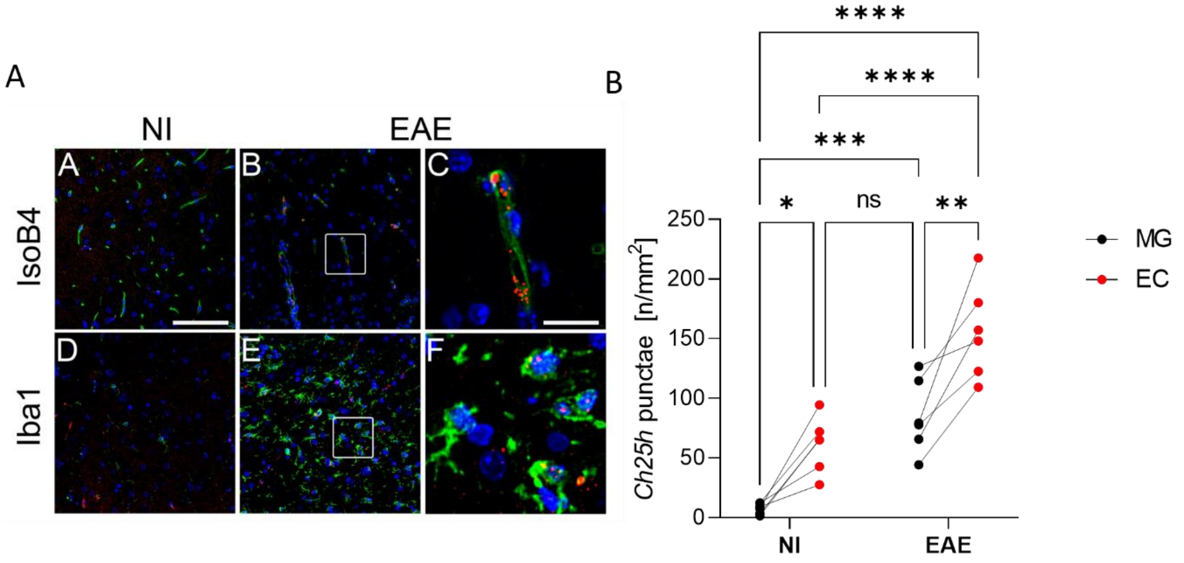

Rebuttal Figure 5. Ch25h mRNA expression in endothelial cell and phagocytes (macrophages/microglia). A): RNAscope fluorescence in-situ hybridization of Ch25h transcripts (red) in the spinal of non-immunized mice (NI) (images A and D, low magnification) and mice and the peak of EAE (images B and E low magnification and images C and F high magnification). Ch25h mRNA expression is shown in endothelial cells (Isolectin B4, IsoB4, in green top panels) and activated macrophages/microglia (Ionized calcium biding adaptor molecule 1, IBA1, in green lower panels). Nuclei are shown in blue. Scale bars, 100 µm; insets, 20 µm. **B)**: quantitative analysis of Ch25h mRNA expression in the spinal cord NI and EAE mice comparing expression in macrophages/microglia (MG) and endothelial cells (EC). N=6/group. ns= non significant, * : $p<0.05$, **: $p<0.005$, ***: $p<0.0005$, ****: $p<0.00005$. p values were determined by two-way ANOVA with Sidak's post hoc test.

2. Figure 4C: The suppressive capacity of the identified CNS-infiltrating neutrophils relies entirely on this figure. However, it lacks a proper control. Simply showing that the addition of CNS-derived neutrophils limits proliferation of anti-CD3/CD28-stimulated T-cells could have multiple explanations. If the authors claim that PMN-MDSCs isolated from the EAE CNS are indeed suppressive they should compare them to "naive" neutrophils isolated for example from blood or bone marrow of a naive mouse. If authors think the suppression is mediated by soluble factors, an alternative approach could be to use conditioned media.

To address this important question, we performed additional experiments to evaluate the suppressive activity of granulocytes within different organs. Live cells $CD45^+$ $CD11b^+$ $Ly6G^+$ $Ly6C^{int}$ were sorted from the bone marrow of naïve and EAE mice and co-cultured with $CD4^+$ T cell isolated from the spleen, labelled with CSFE and stimulated with anti-CD3/28 beads in the exact same conditions as Live cells $CD45^+$ $CD11b^+$ $Ly6G^+$ $Ly6C^{int}$ sorted from the CNS at the peak of EAE in former Figure 4C (Rebuttal Fig. 6 and new Fig. 4C). We did not observed a reduction in the percentage of $CFSE^{low}$ CD4 T cells with BM-derived Live cells $CD45^+CD11b^+Ly6G^+Ly6C^{int}$. These results, combined with the experiment for Arginase-1 staining proposed by referee 3 at point 1B (see below) suggest that CNS derived live cells $CD45^+$ $CD11b^+$ $Ly6G^+$ $Ly6C^{int}$ are indeed PMN-MDSC while the same population derived from either the blood, the spleen or the BM rather correspond to Bona-fide neutrophils. This is line with the current model of PMN-MDSC development proposed by the Contamide et al. suggesting that the suppressive capacity is acquired in peripheral tissues (Condamine *et al*, 2015), more specifically in the CNS in our model. This is also in accordance with what was previously proposed by Knier et al (Knier *et al*, 2018).

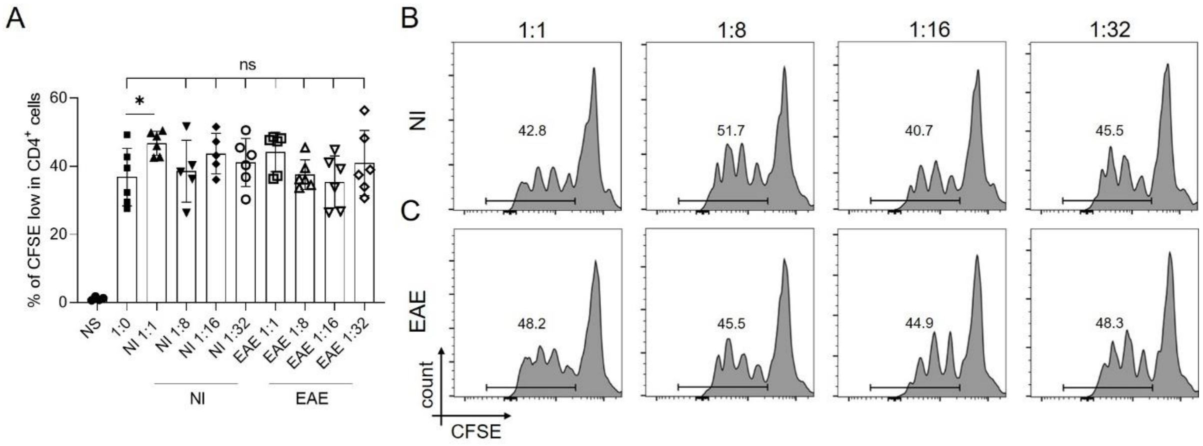

***Rebuttal Figure 6. Proliferation assay of CD4+ T cells co-cultured Live cells CD45+ CD11b+ Ly6G+ Ly6Cint sorted from the bone marrow of* non-immunized (NI) and EAE mice. (A)** Impact of BM-PMN on CD4 T cell proliferation (anti CD3/CD28) assessed by CFSE dilution using flow cytometry. PMN-MDSC were FACS-sorted from the BM of WT mice in non-immunized (NI) mice and at the peak of EAE. NS= non stimulated. n= 6 biological replicates/group**.** ns= non significant, * : $p<0.05$. p values were determined by two tailed unpaired t-test (B) Illustrative images of CFSE analysis.

3. The authors need to better link the accumulation of neutrophils in the CNS with the abrogation of disease. Depletion of circulating neutrophils appears to, counterintuitively, cause accumulation of neutrophils in the CNS. Could the authors instead attempt to boost accumulation of suppressive neutrophils using for example G-CSF or PGE2 administration (or other means)?

We thank the referee for these suggestions. Knier et al showed that G-CSF administration partially attenuates EAE disease course, linking accumulation of suppressive neutrophils and EAE attenuation (Knier *et al.*, 2018). Furthermore, Zehntner et al. also demonstrated that $GR1^{High}$ cells sorted from the CNS could supress $CD4^+$ T cell proliferation (Zehntner *et al*, 2005). Finally, Arg1 expression in CNS $CD45^+$ $CD11b^+$ $Ly6G^+$ $Ly6C^{int}$ cells at the peak of the disease that we describe in referee 3 major point 1b combined the proliferation assay that we performed with PMN-MDSC sorted from the CNS in Fig.4C further indicate that this population shows a suppressive phenotype. We thus consider that there is a substantial amount of evidence showing that a subset of $CD45^+$ $CD11b^+$ $Ly6G^+$ $Ly6C^{int}$ cells infiltrating the CNS during EAE display a suppressive phenotype.

Administration of a stable form of PGE_2 (dm-PGE_2) has also been shown to attenuate EAE (Esaki *et al*, 2010). Given that CNS endothelial-secreted PGE_2 is increased in *Ch25h*^{ECKO} pMBMECs, we asked if PGE_2 administration favors CNS PMN-MDSC accumulation. We reproduced the experiment initially performed by Esaki et al. We confirmed that subcutaneous injections of a stable form of dm-PGE_2, starting from day 7 after immunization attenuates EAE in WT mice (Rebuttal Fig. 6a). However, this phenotype seems to be independent of CNS-infiltrating PMN-MDSC, as neither the percentage nor the absolute number of these cells differed between controls and dm-PGE_2-injected mice (Rebuttal Figure 6b right upper panel). Moreover, absolute numbers and percentage of $Ki67^+$ $CD4^+$ T cells was also similar between these two conditions. We believe that an elevation of the systemic levels of PGE_2 is not sufficient to recapitulate our model and hypothesize that systemic injection of PGE_2 induces PMN-MDSC outside the CNS, but we have not evaluated this hypothesis due to the time allocated to perform the revisions. Knier et al.(Knier *et al.*, 2018) previously suggested that the acquisition of the PMN-MDSC phenotype by granulocytes is restricted to the CNS during EAE. The experiment that we performed to address referee 2, major point 2 and referee 3 major point 1b confirm these results. Additionally, as already mentioned, this is highly consistent with model of PMN-MDSC development proposed by Gabrilovitch. Here, we propose that the increased expression of Ch25h by microvascular endothelial cells during EAE results in increased levels of 25-OHC and decreased levels of PGE_2 which restrain PMN-

MDSC expansion at the level of their extravasation site within the CNS which are microvascular endothelial cells and thus not be modelled by systemic injections of PGE_2. This is again, consistent, with mode of action of 25-OHC which is believed to be autocrine and paracrine.

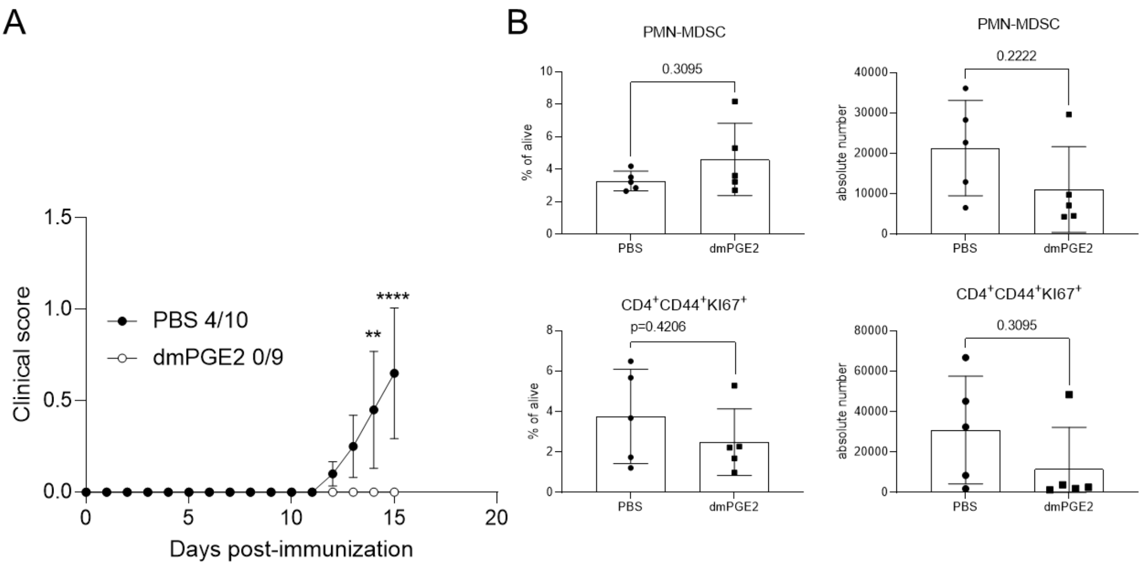

Figure 7. dm-PGE2 impact on EAE disease course and CNS leukocyte infiltration. (A) EAE was induced in eight-week-old C57BL/6 mice. dm-PGE_2 (200 µg/kg) or PBS was injected subcutaneously, 2 times/day starting from day 7 after immunization. Mice were scored and weighted daily until day 15 after immunization. **(B)** Percentage (left panels) and absolute number (right panels) of CNS Live cells $CD45^+CD11b^+Ly6C^{int}$ $Ly6G^+$ (upper panels, PMN-MDSC) and of Live cells $CD45^+CD4^+CD44^+KI67^+$ (lower panels) were determined flow cytometry at day 15 post-immunization. n=5 biological replicates/group. ns= non significant, **: p<0.005 ****: p<0.00005**.** p values were determined by two-way ANOVA with Sidak's post hoc test (a) and by two-tailed unpaired t-test (b).

Minor points:

1. It would be interesting to understand where the neutrophils accumulate inside the CNS. Are they mainly located in subarachnoid/meningeal/perivascular spaces or do they infiltrate the brain parenchyma?

Single cell RNA analysis of CNS myeloid cells during EAE revealed the presence neutrophils in perivascular space, parenchyma, leptomeninges and choroid plexus (Jordao *et al*, 2019). Knier et al., have also identified that $Ly6G^+$ cells interact with B cells in the leptomeninges (Knier *et al.*, 2018). We performed IHC on CNS tissues section of mice during EAE. Briefly, after deparaffination, antigen retrieval was performed using a Pascal Citrate buffer at pH 6.0. To inactivate endogenous peroxidases, slides were incubated with Dako REAL peroxidase-blocking solution (Dako, K0672) and subsequently blocked with Fab Fragment Goat anti-mouse IgG (Jackson ImmunoResearch, 115-007-003) and 2.5% Goat Serum in PBS (Vector Laboratories, S-100). Tissue sections were stained with a rat anti-Ly6G antibody (Biolegend, 127622) diluted in Dako REAL antibody diluent (Dako, S2022) at RT, 1 hour. To visualize the specific signal, anti-rat HRP (Vector Laboratories, MP-7444) together with TSA Vivid 650 (TOCRIS, 7536) was used as secondary antibody and amplification system. After washing, slides were incubated goat serum to avoid subsequent unspecific binding. To perform the vessels staining, sections were incubated with a rabbit anti-van Willebrand Factor antibody (Abcam, ab6994). Conjugated secondary antibodies corresponding to the species was used to visualize specific staining. Nuclei were stained with DAPI (Invitrogen, D1306). Slides were mounted with Fluoromount aqueous mounting medium (Sigma-Aldrich, F4680). For image acquisition, slides were scanned with the Pannoramic 250 FLASH II (3DHISTECH) Digital Slide Scanner at 20× magnification. We identified $Ly6G^+$ cells in the

perivascular space (Rebuttal Fig. 8a), leptomeninges (Rebuttal Fig. 8b), and the parenchyma (Rebuttal Fig. 8c).

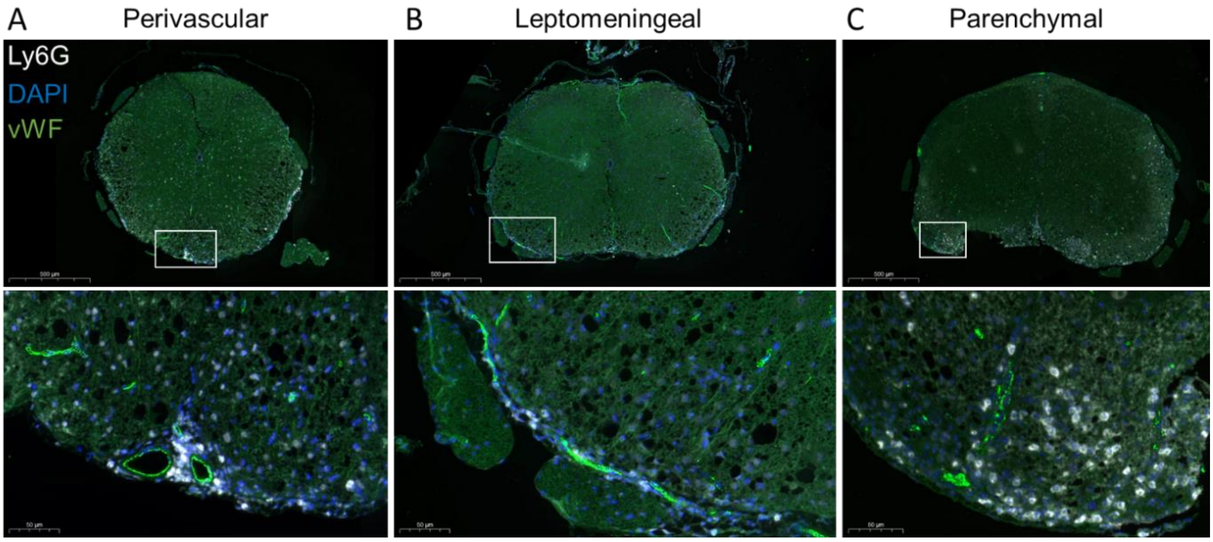

Rebuttal Figure 8. Immunofluorescence analysis using markers for neutrophils (Ly6G), ECs (vWF) and nuclei (DAPI) in spinal cord sections of WT mice at early disease recovery (d17); scale bars 500 μm; higher power; scale bars 50 μm.

2. The authors should report counts instead of % of live cells as the latter can be misleading. See Fig 4f, 6d-f.

We agree that this point was unclear in the initial submission. One of our main finding is increased percentage of PMN-MDSC relative to total live cells and decreased percentage of proliferating $CD4^+$ T cells in the CNS of *Ch25h*^{ECKO} mice compared with *Ch25h*$^{fl/fl}$ mice during EAE. We did not observe significant differences in the abundance of these two populations using absolute numbers as readout (Rebuttal Fig. A, B and new Fig. 4). However, we observed an increased variance of both live cells $CD45^+$ $CD4^+$ $CD44^+$ $Ki67^+$ and live cells $CD45^+$ $CD11b^+$ $Ly6G^+$ $Ly6C^{int}$ (PMN-MDSC) in *Ch25h*^{ECKO} mice compared with *Ch25h*$^{fl/fl}$ mice (F test, P values = 0.0053 and 0.0236 respectively). Furthermore, the ratio PMN-MDSC/proliferating $CD4^+$ T cells was increased while the ratio CD11b/proliferating $CD4^+$ T cell is unchanged (Rebuttal Fig. 9C and D). These data suggests a *remodeling* of CNS infiltrating leukocytes in *Ch25h*^{ECKO} mice in favor of PMN-MDSC expansion. Furthermore, results shown in new Fig. 4 and the additional experiments performed to address the major point 2 of referee 2 and major point 1b of referee 3 increase our confidence that $CD45^+$ $CD11b^+$ $Ly6G^+$ $Ly6C^{int}$ suppress $CD4^+$ T cell proliferation. Moreover, expression of ARG1 (Rebuttal Fig.11 and new Fig. 4E) is reminiscent of pro-regenerative neutrophils (Sas *et al*, 2020) described in a mouse model of optic nerve injury.

Therefore, we propose, that PMN-MDSC expansion *relative* to other immune cell populations contributes to attenuated EAE and that the phenotype that we observe is explained by a qualitative rather than a quantitative change in leukocyte infiltrates. We suggest that a *relative* increase in the fraction of cells displaying immunosuppressive and potentially wound healing functions is sufficient to attenuate EAE clinical scores. However, we do not exclude that this phenomenon could be transient, as in some experiments *Ch25h*^{ECKO} mice displayed a delayed EAE disease course kinetic (Fig. 5A). In summary, we consider that absolute numbers of infiltrating leukocytes might not be the most appropriate readout in our settings. For these reasons, we are using the percentages as our main readout throughout the paper. However, we added absolute numbers in new Fig. 4H and a discussion regarding these relative changes in our manuscript.

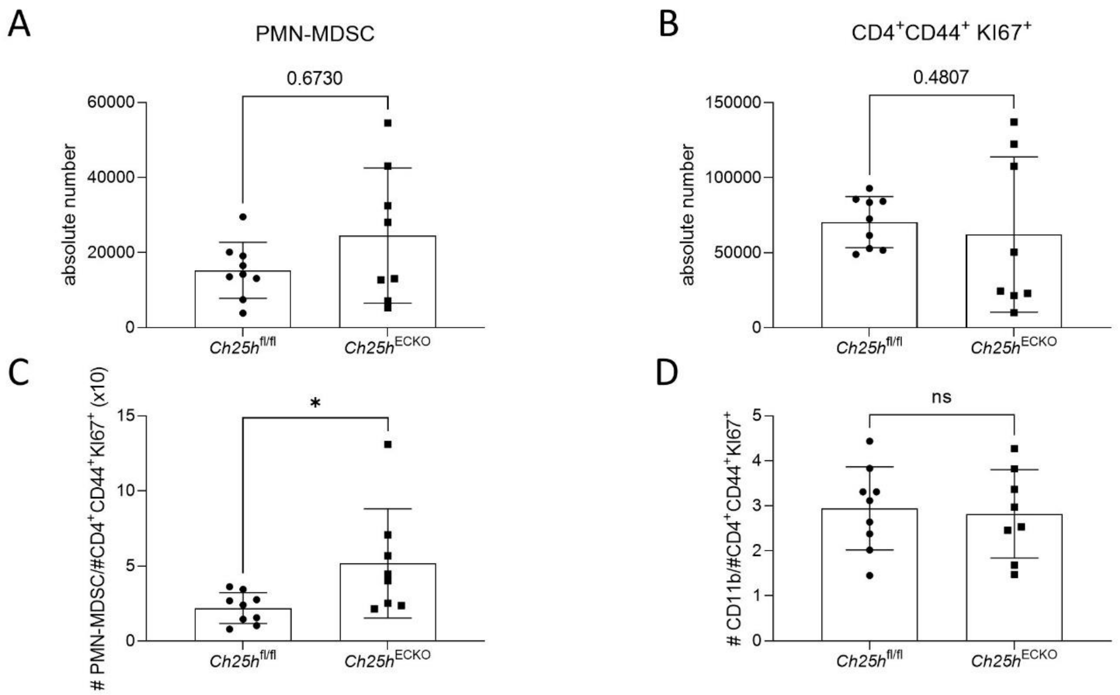

Rebuttal Figure 9 .Absolute number of CNS PMN-MDSC and proliferating CD4 T cell in Ch25h$^{fl/fl}$ and Ch25h^{ECKO} mice . (A): Absolute number of CNS Live cells CD45^{+}CD11b^{+}Ly6C^{int}Ly6G^{+} and **(B)** of Live cells CD45^{+}CD4^{+}CD44^{+}KI67^{+} were determined by flow cytometry in Ch25h^{ECKO} and Ch25h$^{fl/fl}$ mice at day 16 post-immunization. **(C)** : ratio of absolute number of PMN-MDSC and proliferation CD4 T cells. **(D):** ratio of absolute number of Live cells CD45^{+} CD11b $^{+}$(Cd11b). Ch25h$^{fl/fl}$ n=9, Ch25h^{ECKO} n=8 biological replicates. p values were determined by two tailed unpaired t-test. ns= non significant, * : p<0.05.

3. The GBM data is interesting but is mostly correlative and not backed up by any experimental data in for example a mouse model. Taking this into account, it is my opinon that the authors should revise their discussion on the GBM data accordingly.

We have now removed the GBM data from the manuscript.

4. For figure 3 it would be helpful to guide the reader by including a schematic figure of cholesterol metabolism including enzymes and metabolite steps.

We have now added a schematic figure of cholesterol metabolism (Rebuttal Figure 10) in the new Figure EV1.

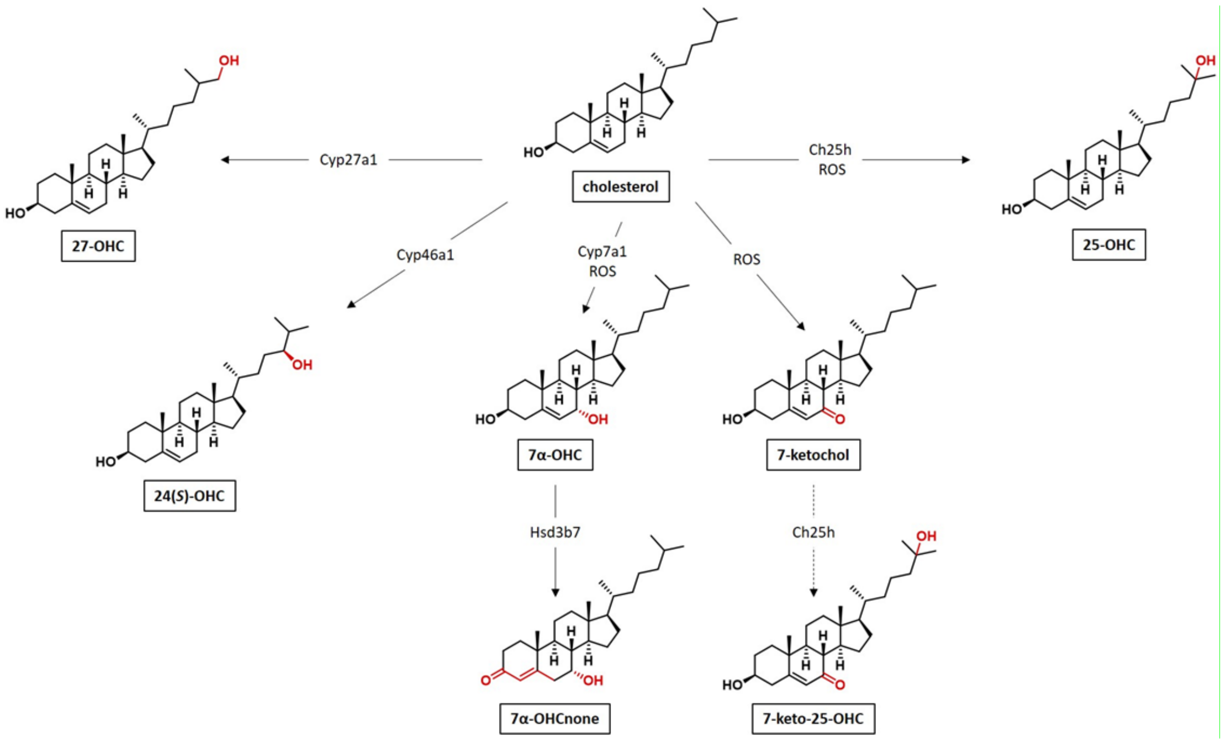

Rebuttal Figure 10. Schematic illustration of cholesterol metabolism.

Referee #3:

The manuscript by Florian Ruiz et al. "Endothelial-derived oxysterols regulate myeloid-derived suppressor cells at the blood-brain barrier" describes a new role for the enzyme Ch25h and its product 25-OHC in EAE, namely the inhibition of myeloid suppressor cell expansion in the inflammatory process at the blood brain barrier. Using a new mouse strain, made in the lab, which reports expression of Ch25h and which can be used for tissue-specific deletion of the enzyme, they show that endothelial cells of the CNS are the main cell type expressing Ch25h in EAE and that its deletion in endothelial cells and importantly in CNS-ECs leads to a delayed and lowered EAE, similar to mice lacking CH25h body-wide as shown in a previous paper by the lab. From that point they show that the lack of Ch25h in inflamed endothelial cells changes expression of lipid biosynthetic pathways leading finally to a shift from 25-OHC to PGE2 in mice lacking Ch25h in ECs. In vitro data shows that PGE2 promotes differentiation of MDSCs whereas 25-OHC strongly inhibits their differentiation. These findings could explain the found expansion of MDSCs in the CNS of CH25hEC-KO mice with EAE compared to control mice. Finally, depletion of mature neutrophils at DPI 14 led to a near complete resistance of EAE in CH25hEC-KO mice and a further expansion of MDSCs in the CNS. Additionally, one figure shows analyses of data from human glioblastoma ECs in context to Ch25h expression and its similarity to ECs of mice lacking Ch25h in EAE CNS.

This manuscript is very interesting, well written and the experiments are thoroughly performed. Nevertheless, some points are not clear enough yet for the main claim to be made and further experiments should be performed to make the main conclusions more solid.

1. The main claim of the paper is that EAE is lower in CH25hEC-KO due to expansion of MDSCs in the CNS. Several points are unclear here. a) Expansion: in all experiments showing MDSCs in the CNS only

percentages of live cells are shown. Since EAE is lower in CH25hEC-KO mice, also infiltration of T cells and of monocytes should be reduced. Hence, the higher percentage of MDSCs in the CNS in EAE in these mice might only be relative. Therefore, it is necessary to determine the absolute cell numbers and to show them next to the percentages. This accounts also for data showing T cell-infiltrates.

We propose that the expansion of PMN-MDSC is relative to the other leukocyte populations. We now show the absolute numbers in new Fig. 4H and discussed our reasoning in the manuscript. Please refer to Referee 2 minor point 2 for the discussion.

b) MDSCs in the CNS: As also discussed by the authors, it is difficult to distinguish PMN-MDSCs from normal PMNs in the mouse and an alternative scenario of the effectiveness of the PMN depletion might be their pathogenic effect in EAE as claimed by several others previously combined with a lack of depletion of PMNs in the CNS. To be sure that the CNS PMNs are indeed MDSCs, one should perform an Arginase 1 staining (e.g. intracellular with clone A1exf5) and compare this to PMNs in the blood, spleen and BM.

We thank the referee for this advice. We performed additional EAE experiment and show that, in agreement with the data obtained from the suppression assays for the major point 2 of referee 2, Arg1 expression is restricted to CNS Live cells $CD45^{+}CD11b^{+}Ly6C^{int}$ $Ly6G^{+}$ during EAE (Rebuttal Fig. 11 and new Fig. 4E and F). Therefore, we propose that $CD45^{+}CD11b^{+}Ly6C^{int}Ly6G^{+}$ acquire their suppressive properties in the CNS in our model, as already suggested (Knier *et al.*, 2018). Those results are now included in new Fig. 4 in addition to the suppression assay.

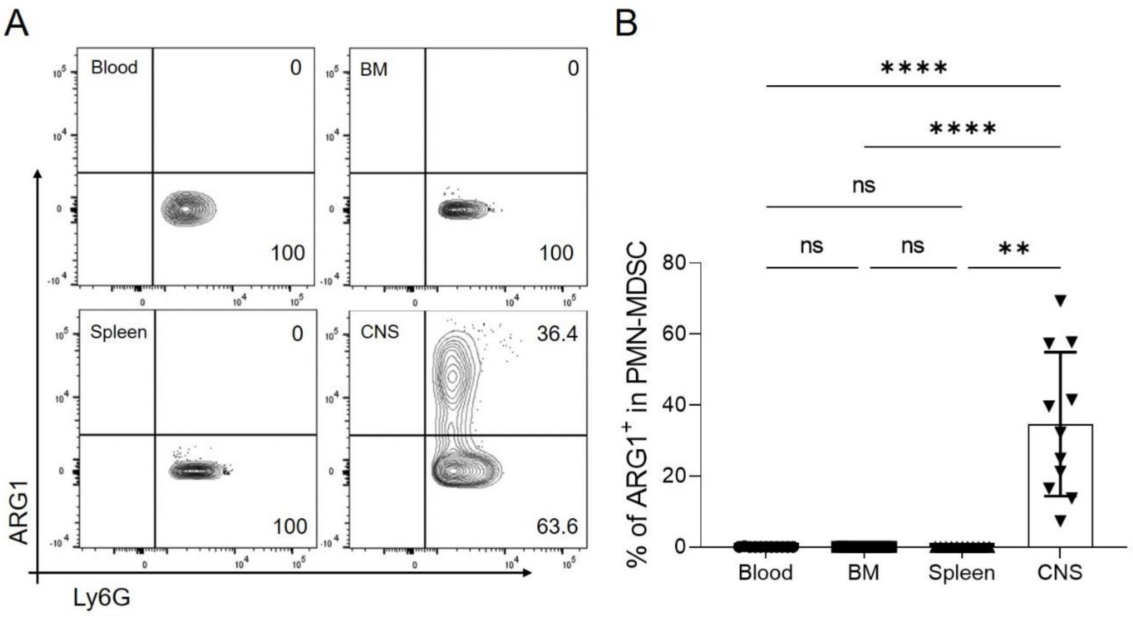

Rebuttal Figure 11. ARG1 expression in Live cells $CD45^{+}CD11b^{+}Ly6C^{int}Ly6G^{+}$ in the bone marrow, blood, spleen and CNS during EAE. A) representative contour plots of ARG1 staining in the bone marrow (BM), blood, spleen and CNS assessed by flow cytometry and gated of live cells $CD45^{+}CD11b^{+}Ly6C^{int}Ly6G^{+}$.**B)** as in **a** except that quantitative analysis is shown. n= 11 biological replicates. ns= non significant, * : p<0.05, ***: p<0.0005 ****: p<0.00005**.** p values were determined by Kruskal-Wallis test with Dunn's post test.

c) How do 25-OHC/PGE2 act on immature PMN? Unfortunately, PMN/ MDSCs of controls and CH25hEC-KO were only compared in the CNS and not in blood, spleen and BM (eg. Figure 4f). This should at least clarify whether 25-OHC/PGE2 have a more systemic or local impact at the CNS.

We showed in former Figure S5B, the percentage of Live cells, $CD45^{+}$ $CD11b^{+}$ $Ly6C^{int}$ $Ly6G^{+}$ (neutrophils) in the blood of *Ch25h*$^{fl/fl}$ and *Ch25h*^{ECKO} in isotype and combo treated mice. We did not observe differences in the percentage of neutrophils in the blood of the isotype treated mice suggesting that

the expansion of CD45$^+$ CD11b$^+$ Ly6G$^+$ Ly6C^{int} cells is CNS restricted. This result is now discussed in the new manuscript and included in new Fig. 5E and F. Please also refer to point d below.

d) Are MDSCs also expanded in the CNS of CH25h-BBBKO mice? Since CH25hEC-KO mice may show an activation of ECs in the whole body and an increased level of PGE2 over 25-OHC, it might be possible that the increase of PMN/MDSCs occurs already at the level of the BM and due to an expansion there, more of these cells may migrate to the CNS during inflammation. Therefore, it is also important to know whether also CH25h-BBBKO mice, which show the same EAE phenotype, also show an enhanced level of PMN/MDSCs in the CNS.

We thank the reviewer for this important question. We repeated the experiment in *Ch25h*$^{fl/fl}$ and *Ch25h*^{BBKO} and showed that *Ch25h*^{BBKO} mice phenocopied *Ch25h*^{ECKO} for both EAE attenuation and CNS PMN-MDSC expansion (Rebuttal Fig. 12A and B and new Fig. EV2). We now show this result in the manuscript together with the corresponding disease course.

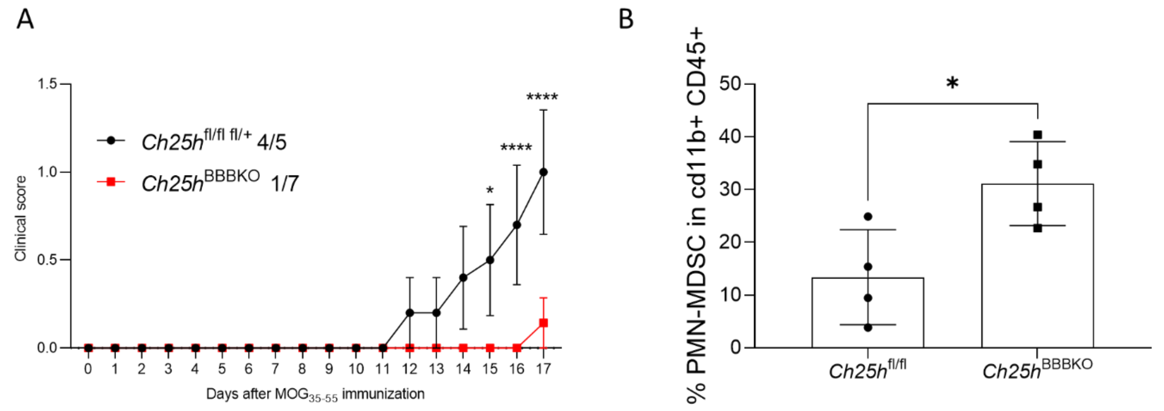

Rebuttal Figure 12 EAE disease course and CNS PMN-MDSC in Ch25^{BBBKO} mice. A) EAE disease course in Ch25h^{BBBKO} (n=9) and Cre negative littermates (Ch25h$^{fl/fl}$, n=5). **B)** percentage of CNS PMN-MDSC (live cells CD45$^+$CD11b$^+$Ly6C^{int}Ly6G$^+$) gated on CD45$^+$CD11b$^+$population assessed by flow cytometry in Ch25h^{BBBKO} mice and Ch25h$^{f/fl}$ from the EAE disease shown in A) at day 15 post-immunization. * : $p<0.05$, ****: $p<0.00005$. p values were determined by two-way ANOVA with Sidak's post hoc test (a) and by two-tailed unpaired t-test(b).

2. Since EAE is mediated by T helper cells, an analysis of CNS-infiltrating T cells is lacking in respect to Th17/Th1/ThGMCSF/Tregs. These should also be analyzed in spleen/dLNs and blood. All in % and in absolute numbers.

We now evaluated different subsets of T cells both in percentage and absolute numbers of Th17 (CD4$^+$CD44$^+$RORgt$^+$IL-17$^+$), Th1 (CD4$^+$CD44$^+$Tbet$^+$IFN-γ^+), ThGMCSF (CD4$^+$CD44$^+$GM-CSF$^+$), Tregs (CD4$^+$CD44$^+$Foxp3$^+$) in the CNS, blood, LN and spleen at the peak of EAE disease. While we observed different percentages and absolute numbers of different T cell subsets between organs, we did not observe significant alteration in the CD4$^+$ T cell subsets within the different organs analysed in Ch25h^{ECKO} mice compared with Ch25h$^{fl/fl}$ mice (Rebuttal Fig. 19). Overall, those results are in accordance with the data from major point 2 of Referee 2. This suggest that the impact of PMN-MDSC expansion in our model does not target a specific CD4$^+$ T cell subset. However, we do not exclude that, FACS analysis performed at different time points of EAE, especially at early time points of the disease, could differ.

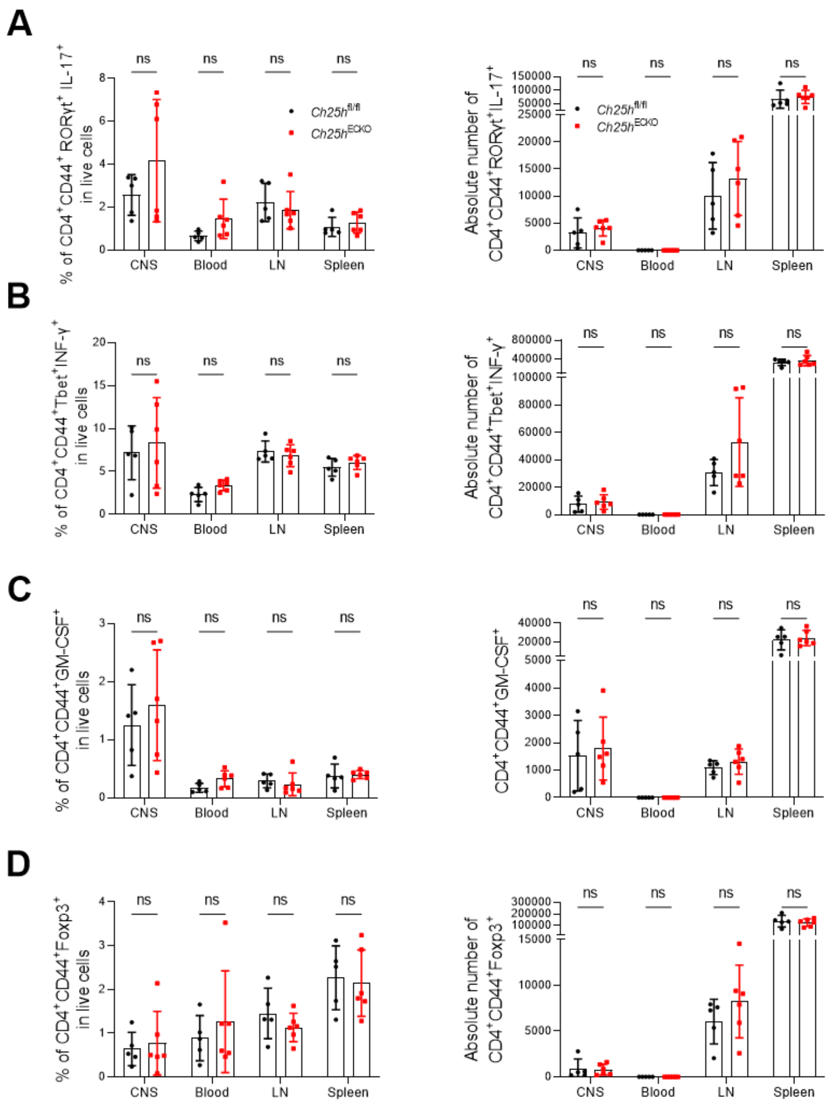

Rebuttal Figure 13 Flow cytometry analysis of Th17/Th1/ThGMCSF/Tregs in ch25h$^{fl/fl}$ and Ch25h^{ECKO} mice during EAE. Left panels are showing percentage of live cells and right panels absolute numbers of live cells CD45^{+}CD4^{+}CD44^{+} **(A)** Th17 (CD4^{+}CD44^{+}RORγt^{+}IL-17^{+}) , **(B)** Th1 (CD4^{+}CD44^{+}Tbet^{+}IFN–γ^{+}) **(C)** ThGMCSF (CD4^{+}CD44^{+}GM-CSF^{+}), **(D)**: Tregs (CD4^{+}CD44^{+}Foxp3^{+}). Blood, CNS, draining lymph nodes (LN) and spleen are compared. *Ch25h*$^{fl/fl}$ n=5, *Ch25h*^{ECKO} n=6. ns= non significant, * : $p<0.05$. p values were determined p values were determined by two-way ANOVA with Sidak's post hoc test.

3. Ch25h is important in generating the EBI2 ligand 7a,25-OHC. This ligand was previously shown to be upregulated in EAE in the CNS. Since your data show that EC are the main cells expressing Ch25h in the CNS in EAE one would expect a lack of 7a,25-OHC upregulation in the CNS of CH25h-BBBKO mice. Showing this would make your findings using the reporter mouse even more convincing. In the same line a western blot against CH25h of full CNS vs. CNS-ECs of healthy vs. EAE of control vs. CH25hEC-KO would be a great confirmation of your reporter data.

Ch25h is important for generating 25-OHC by hydroxylation of cholesterol on the C25 position. 25-OHC can be further metabolized and is the precursor of the biosynthesis of 7α,25-OHC. Ch25h is also important for the generation of 7-keto-25-OHC from the precursor 7α-OHC. We did not detect 7α,25-OHC *in-vitro.* It is possible that it is under the limit of detection of our technic. 25-OHC levels have been shown to be increased in the spinal cord of EAE mice (Wanke *et al.*, 2017).

To answer the question of the referee, we now measured oxysterols from spinal cords collected from *Ch25h*$^{fl/fl}$ and *Ch25h*^{ECKO}/*Ch25h*^{BBKO} female mice during EAE (16 days after immunization). We observed a significant reduced production of both 25-OHC and 7-keto-25OHC in the mice lacking Ch25h in ECs (Ch25h^{ECKO}/Ch25hB^{BBKO}) compared to the controls (Ch25h$^{fl/fl}$) (rebuttal Figure 14 left and middle panel and new Figure EV1). 7α,25-OHC was not detected. 24*(S)*-OHC levels, the main oxysterol of the CNS, was higher than 25-OHC and 7-keto-OHC but did not differ between the two mouse groups (Rebuttal Fig. 14 and new Fig. 3D). As Ch25h remains expressed in other cell types than ECs and as our mouse model is a Knockdown and not a full KO, we did not expect a higher decrease in 25-OHC and 7-keto-25-OHC levels. This is in accordance with the results shown in Rebuttal Fig. 5 and Rebuttal Fig. 15 b showing Ch25h expression in microglia.

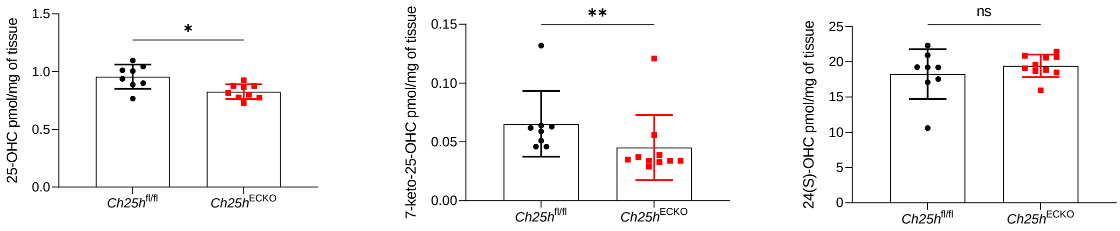

Rebuttal Figure 14: 25-OHC is decreased in the spinal cord in mice lacking Ch25h solely in the ECs. 25-OHC, 7-keto-25 OHC and 24*(S)*-OHC levels in pmol/mg of the spinal cord tissue of female mice 16 days after immunization for EAE.

4. Ch25h expression in EAE: Microglia cells should be properly stained with CD11b and CD45 and then gated on CD11b (int) and CD45(int) for analysis of reporter expression. Ter119 staining to exclude erythrocytes should be performed on a separate channel. In addition, the FACS staining should also be shown reversed by gating on all live cells expressing Ch25h-eGFP and then checking in which population these positive cells fall. By such a gating a staining of CNS from CH25hEC-KO mice should reveal a near complete absence of eGFP in the live cell population. Since FACS shows only a part of CNS resident cells, histology against eGFP in Ch25h reporter mice with EAE should reveal a more complete picture of Ch25h expression in the CNS in EAE.

We thank the referee for this important question. We have now performed the experiment (rebuttal Fig. 15) as suggested with a FACS staining for Ch25h-eGFP on leukocytes extracted with a different protocol than for ECs to properly assess microglia. Briefly, CNS tissue was digested in a DMEM containing collagenase D (2.5 mg/ml Sigma) and Dnase 1 (1 mg/ml Sigma) to give a single-cell suspension. Of note, for ECs, brain enzymatic digestion is performed with Collagenase/Dispase (2mg/ml), DNAse I (10µg/ml) and Nα-Tosyl-L-Lysin-chlormethyl keton hydrochlorid (TLCK, 0.147µg/ml). Using this extraction protocol, we detected a Ch25h-eGFP signal in microglia in the CNS of non-immunized (NI) mice at low levels with an almost significant increase of Ch25h expression in the CNS of EAE mice (Rebuttal Fig. 15). We have now adapted the text of our manuscript accordingly, included those new results in Appendix Fig. S1 and discussed them in the manuscript on page 5 – lines: 132-137. For a detailed discussion of that specific point, please refer to referee 2 major point 1.

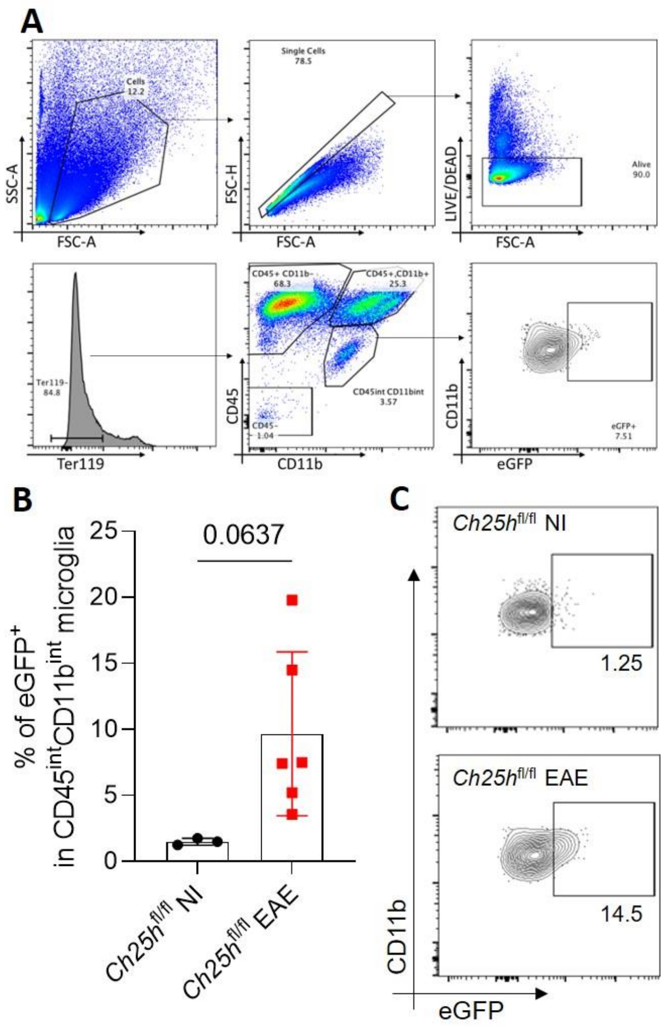

Rebuttal Figure 15: Analysis of Ch25h-eGFP expression in microglia. A) Gating strategy for flow cytometry analysis of Ch25h-eGFP expression in TER119^{-}CD45^{int} CD11b^{int} microglial cells. **B)** Flow cytometry analysis of Ch25h-eGFP expression in TER119^{-} CD45^{+}CD11b^{int} microglia in non-immunized (NI) Ch25h$^{fl/fl}$ mice (n=3 biological replicates) and at day 17 post-immunization (EAE, n= 6 biological replicates). P value was determined by unpaired Student's t test. **C)** Representative FACS plot of Ch25h-eGFP in NI and EAE Ch25h$^{fl/fl}$ mice.

The reverse gating strategy is a very good suggestion. However the necessity to use two different extraction methods (for the leukocytes and the ECs) impairs the comparison of the different cell type. Indeed, in the extraction for the leukocytes, ECs are not detected as they necessitate specific digestion protocols (collagenase/dispase) to be released from their entrapment in the basement membrane. We however performed an RNA-scope and additional FACS analysis that confirm and compare the expression of Ch25h in ECs and also in microglia (Rebuttal Fig. 5 and new Fig. 1A and B).

Minor Points:

5. Although very interesting, the data shown on GBM ECs is rather confounding the overall story and not necessary for the main points to make. I would suggest to omit this and to put this rather in another small publication.

We have now removed the GBM data from the manuscript as it was proposed by 2 referees.

6. Some histology of infiltration and demyelination of controls versus CH25hEC-KO in EAE would be nice to have to see whether there might be also other qualitative changes.

We performed H&E staining in Ch25h^{ECKO} mice and Ch25h$^{fl/fl}$ mice during EAE and found a significant reduced number of lesions Ch25h^{ECKO} compared to Ch25h$^{fl/fl}$(rebuttal Fig. 16 and new Fig. EV2).

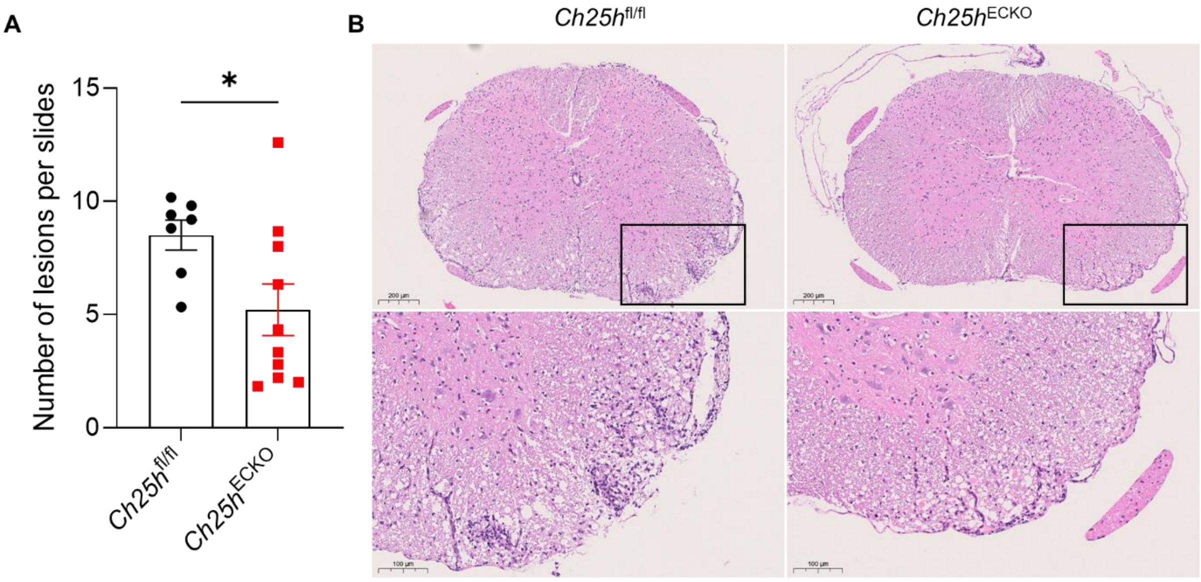

Rebuttal Figure 16. Histopathological A) quantifications and B) representatives staining of spinal cord sections of immunized *Ch25h*$^{fl/fl}$ mice (n=7) and *Ch25h*^{ECKO} (n=10) at day 21 post-immunization for cellular infiltration (H&E). Five sections per mouse were quantified. Scale bars 200 µm (top panels), 100 µm (bottom panels). *P* values were determined by unpaired Student's *t* test

7. For EAE experiments it would be nice to see for each group shown the exact N number of the mice directly in the figure.

We have now included the number of mice for each EAE graphs on the Figures.

8. Mean max score: Was this calculated only of diseased mice?

Mean and max scores are calculated for all the EAE mice.

9. Figure 2e seems to show the averaged data of 3 measurements for each condition. I would suggest to show them individually.

We have modified Fig. 2E accordingly and are now showing the z-score of CPM for each replicate.

10. Figure 4c shows that PMN-MDSCs can inhibit T cell proliferation. A control showing the specificity of these cells by using PMN from BM would strengthen that point.

We have now performed additional experiment and have included a suppressive test with PMN-MDSCs from the BM showing that they are not able to inhibit T cell proliferation (Rebuttal Fig. 5 and new Fig. 4). See referee 2, question 2.

*11. Fig. 4d Correlation should be rather -0.68, what do the ** mean here?*

The former figure 4D now 4F has been modified accordingly.

12. Figure 6a: There seems to be at least some impact of the combo treatment also in controls.

This is correct, there is a trend towards a protective impact of the combo treatment in controls that did however not reach statistical significance in our setting.

13. In Figure 6e and f the comparison of controls vs. CH25hEC-KO in EAE should also be shown.

We have now modified the new Fig. 5E and 5F.

14. For Figure 6f one should rather show bars than lines since the loss of PMNs occurs most likely only on , day 14 and not before.

We now show the results on bar graph and not with lines in new Fig. 5F.

15. Figure 6f right: axes labels were forgotten

This has been corrected in new Figure 5.

16. Page 16 mentions deletion of Ch25h in GBM ECs although it is rather likely "not expressed" what was meant.

We have now removed the section on GBM as proposed by referees 1 and 2.

17. Although in general well written the grammar needs to be checked.

The grammar has been checked.

References

Condamine T, Mastio J, Gabrilovich DI (2015) Transcriptional regulation of myeloid-derived suppressor cells. *J Leukoc Biol* 98: 913-922

Esaki Y, Li Y, Sakata D, Yao C, Segi-Nishida E, Matsuoka T, Fukuda K, Narumiya S (2010) Dual roles of PGE2-EP4 signaling in mouse experimental autoimmune encephalomyelitis. *Proc Natl Acad Sci U S A* 107: 12233-12238

Jordao MJC, Sankowski R, Brendecke SM, Sagar, Locatelli G, Tai YH, Tay TL, Schramm E, Armbruster S, Hagemeyer N *et al* (2019) Single-cell profiling identifies myeloid cell subsets with distinct fates during neuroinflammation. *Science* 363

Knier B, Hiltensperger M, Sie C, Aly L, Lepennetier G, Engleitner T, Garg G, Muschaweckh A, Mitsdorffer M, Koedel U *et al* (2018) Myeloid-derived suppressor cells control B cell accumulation in the central nervous system during autoimmunity. *Nat Immunol* 19: 1341-1351

Munji RN, Soung AL, Weiner GA, Sohet F, Semple BD, Trivedi A, Gimlin K, Kotoda M, Korai M, Aydin S *et al* (2019) Profiling the mouse brain endothelial transcriptome in health and disease models reveals a core blood-brain barrier dysfunction module. *Nat Neurosci* 22: 1892-1902

Mutemberezi V, Masquelier J, Guillemot-Legris O, Muccioli GG (2016) Development and validation of an HPLC-MS method for the simultaneous quantification of key oxysterols, endocannabinoids, and ceramides: variations in metabolic syndrome. *Anal Bioanal Chem* 408: 733-745

Saadane A, Mast N, Trichonas G, Chakraborty D, Hammer S, Busik JV, Grant MB, Pikuleva IA (2019) Retinal Vascular Abnormalities and Microglia Activation in Mice with Deficiency in Cytochrome P450 46A1-Mediated Cholesterol Removal. *Am J Pathol* 189: 405-425

Sas AR, Carbajal KS, Jerome AD, Menon R, Yoon C, Kalinski AL, Giger RJ, Segal BM (2020) A new neutrophil subset promotes CNS neuron survival and axon regeneration. *Nat Immunol* 21: 1496-1505

Wanke F, Moos S, Croxford AL, Heinen AP, Graf S, Kalt B, Tischner D, Zhang J, Christen I, Bruttger J *et al* (2017) EBI2 Is Highly Expressed in Multiple Sclerosis Lesions and Promotes Early CNS Migration of Encephalitogenic CD4 T Cells. *Cell Rep* 18: 1270-1284

Zehntner SP, Brickman C, Bourbonniere L, Remington L, Caruso M, Owens T (2005) Neutrophils that infiltrate the central nervous system regulate T cell responses. *J Immunol* 174: 5124-5131

1st Revision - Editorial Decision 8th Dec 2022

Dear Prof. Pot,

Thank you for the submission of your revised manuscript. We have now received the enclosed reports from the referees. Referee 1 still has a few minor suggestions that I would like you to address and incorporate before we can proceed with the official acceptance of your manuscript.

A few editorial requests will also need to be addressed:

- Your manuscript has 5 main figures but separate results and discussion sections. Please either add one more main figure to turn this into an article, or combine the results and discussion sections to publish it as a short report. You can find more information about our article types in our guide to authors online.

- Please correct the conflict of interest subheading to "DISCLOSURE AND COMPETING INTERESTS STATEMENT"

- Please remove the author credits from the ms file. We now use CRediT to specify the contributions of each author in the journal submission system. CRediT replaces the author contribution section. Please use the free text box to provide more detailed descriptions, if you wish. See also guide to authors https://www.embopress.org/page/journal/14693178/authorguide#authorshipguidelines.

- Some funding information is missing in our online manuscript handling system, please add.

- The Supplementary Tables 1-5 need to be renamed Datasets EV1-EV5 and called out accordingly; the legends should be removed from the manuscript text (legend for Table S5 is missing) and inserted in a separate sheet of each Excel file. Supplementary Table 5 is not called out, please add this callout.

- The page numbers are missing from the table of content of the APPENDIX file, please add. The figures in the file and table of content should be renamed to Appendix Figure S1-S3; Appendix Supplementary Tables 1-5 should be removed from the table of content.

- I attach to this email a related ms file with comments by our data editors. Please address all comments in the final ms.

I would like to suggest some minor changes to the title and abstract. Please let me know whether you agree with the following (and please clarify my questions):

Endothelial-derived oxysterols inhibit myeloid-derived suppressor cell expansion at the blood-brain barrier

or

Endothelial cell-derived oxysterol ablation attenuates experimental autoimmune encephalomyelitis

The vasculature is a key regulator of leukocyte trafficking into the central nervous system (CNS) during inflammatory diseases including multiple sclerosis (MS). However, the impact of endothelial-derived factors on CNS immune responses remains unknown. Bioactive lipids, in particular oxysterols downstream [downstream of? or: products of?] Cholesterol-25-hydroxylase (Ch25h), promote neuroinflammation but their functions in the CNS are not well understood.
Using a floxed-reporter Ch25h knock-in mice, we trace Ch25h expression to CNS endothelial cells (ECs) and myeloid cells and demonstrate that Ch25h ablation specifically from ECs attenuates experimental autoimmune encephalomyelitis (EAE). Mechanistically, inflamed Ch25h-deficient CNS ECs display altered lipid metabolism favoring polymorphonuclear myeloid-derived suppressor cell (PMN-MDSC) expansion, which suppresses encephalitogenic T lymphocyte proliferation. Additionally, endothelial Ch25h-deficiency combined with the mobilization of immature neutrophils in the circulation [??] nearly completely protects mice from EAE. Our findings reveal a central role for CNS endothelial Ch25h in promoting neuroinflammation by inhibiting the expansion of immunosuppressive myeloid cell populations.

I look forward to seeing a final version of your manuscript as soon as possible. Please use this link to submit your revision: https://embor.msubmit.net/cgi-bin/main.plex

Best regards,
Esther

Esther Schnapp, PhD
Senior Editor
EMBO reports

Referee #1:

This paper links 25-hydroxycholesterol (25-OHC) and prostaglandin E2 (PGE2) produced in mouse brain endothelial cells with the severity of EAE, a mouse model of multiple sclerosis. I have only minor suggestions for the authors to consider prior to publication.

1.Line 183 - 184. It is surprising that the cholesterol synthesis pathway was not changed by IL-1β stimulation or Ch25h deletion. This would argue against the suggestion on lines 447 - 451 that a reduction of 25-OHC results in an increase in FADS2 expression through relief of SREBP2 inhibition. Similarly on lines 464 - 467, if deletion of Ch25h does not alter cholesterol synthesis, then it should not relieve inhibition of SREBP1 processing either.
2. Figure 3C. While the authors give concentrations in molarity, which is good for comparison of relative abundance, it is not possible for the reader to know how much oxysterol is secreted per e.g. million cells. This is important if the work is to be repeated by others. The reader needs to know how many cells are present per unit volume if molarity is to be given. I would also strongly recommend that the authors give the internal standards used for quantification in the supplementary data and included relevant chromatograms and mass spectra. This is important as the community moves towards minimal reporting standards. I accept that this information was given in the rebuttal to previous reviews.
3. Line 237-238. A difference is either significant or it is not, no need for the qualifying comment "just reaching".
4. Line 254 - 258. Again, it would be helpful if the list of internal standards was given, this is particularly important with respect to the eicosanoids in Figure 3G. The individual points should be highlighted in Figure 3G.
5. Line 409. I recommend the authors include the chromatogram displaying the detection of 7-keto-25-OHC in the medium. This is an elusive oxysterol and as the authors have the data I recommend they include it, at least as a supplemental. Remember there a very few replicated reports of this molecule in the literature.

In summary a very nice paper that could be published as it is, but would be improved if the authors included the suggested changes.

Referee #2:

The authors have addressed all my concerns satisfactorily. I would recommend publication of the manuscript in its current form.

Referee #3:

The revised version has answered all my major points thoroughly and is a nice piece of research. I wasn't able to locate the synopsis though.

POINT-BY-POINT RESPONSE TO REVIEWERS COMMENTS

Referee #1:

This paper links 25-hydroxycholesterol (25-OHC) and prostaglandin E2 (PGE2) produced in mouse brain endothelial cells with the severity of EAE, a mouse model of multiple sclerosis. I have only minor suggestions for the authors to consider prior to publication.

1. Line 183 - 184. It is surprising that the cholesterol synthesis pathway was not changed by IL-1β stimulation or Ch25h deletion. This would argue against the suggestion on lines 447 - 451 that a reduction of 25-OHC results in an increase in FADS2 expression through relief of SREBP2 inhibition. Similarly on lines 464 - 467, if deletion of Ch25h does not alter cholesterol synthesis, then it should not relieve inhibition of SREBP1 processing either.

We thank the reviewer for this comment. We propose that the release of SREBP2 inhibition is a plausible hypothesis for the increased expression of FADS2. The conclusion that can be drawn from the unbiased gene set enrichment analysis is that, when comparing *Ch25h*^{ECKO} and *Ch25h*$^{fl/fl}$ PMBMECs at 24 hours after IL-1β stimulation, the "regulation of the cholesterol metabolic process" is not among the most significantly enriched pathways in the differentially expressed genes (New Fig. 3C). This does not necessarily indicates that this pathway is not affected by *Ch25h* deletion in endothelial cells and might come up has significantly altered in a biased analysis. However, we agree that the description of these results was confusing. Therefore, we have rephrased the manuscript new lines 185-187.

2. Figure 3C. While the authors give concentrations in molarity, which is good for comparison of relative abundance, it is not possible for the reader to know how much oxysterol is secreted per e.g. million cells. This is important if the work is to be repeated by others. The reader needs to know how many cells are present per unit volume if molarity is to be given. I would also strongly recommend that the authors give the internal standards used for quantification in the supplementary data and included relevant chromatograms and mass spectra. This is important as the community moves towards minimal reporting standards. I accept that this information was given in the rebuttal to previous reviews.

We have now included the internal standards and relevant chromatograms in the Appendix Figure S2A Related to new Figure 4. We have also added the information in the "Material and Methods", lines 632-634.

Concerning the question of the cell number, pMBMECs are cultivated in 96 well plates and plated at a concentration of 51 000 digested capillaries/cm2 onto matrigel-coated wells. The purpose of the digestion step of the vascular pellet prior to pMBMECs seeding is not to obtain single cells but capillary fragment, which is the approach that gives the best results for microvascular ECs culture. Therefore, the seeding density is not based on the number of single cells but on the number of capillary fragments. Oxysterol measurements were performed when cells were confluent to avoid a potential proliferation bias induced by IL-1β stimulation. Furthermore IL-1β is only added in the culture when cells are confluent for 24hours. Thus, the cell count was not performed at the time of supernatant collection and we can thus not express the oxysterols secreted per million cells. We have detailed the experimental methods in our manuscript, Lines 687- 702.

3. Line 237-238. A difference is either significant or it is not, no need for the qualifying comment "just reaching".

We agree and have now changed the text accordingly (new Lines 240-241)

4. Line 254 - 258. Again, it would be helpful if the list of internal standards was given, this is particularly important with respect to the eicosanoids in Figure 3G. The individual points should be highlighted in Figure 3G.

The list of internal standards with respect to the eicosanoids in new Figure 4G is now provided in the dataset EV5.

We have now included the individual points in new Figure 4G.

*5. Line 409. I recommend the authors include the **chromatogram** displaying the detection of **7-keto-25-OHC in the medium (figure added in appendix)**. This is an elusive oxysterol and as the authors have the data I recommend they include it, at least as a supplemental. Remember there a very few replicated reports of this molecule in the literature.*

We have now included the chromatogram of selected measured oxysterols and eicosanoids in the medium including 7-keto-25-OHC in the Appendix Figure S2 Related to Figure 4.

In summary a very nice paper that could be published as it is, but would be improved if the authors included the suggested changes.

We thank the reviewer for his constructive comments and suggestions that have largely contributed to the improvement of our manuscript.

Referee #2:
The authors have addressed all my concerns satisfactorily. I would recommend publication of the manuscript in its current form.
We also thank the reviewer for his constructive comments and suggestions that have largely contributed to the improvement of our manuscript.

Referee #3:
The revised version has answered all my major points thoroughly and is a nice piece of research. I wasn't able to locate the synopsis though.

We also thank the reviewer for his constructive comments and suggestions that have largely contributed to the improvement of our manuscript

Editorial requests:
1. Your manuscript has 5 main figures (new figure # Changed in text and changed in appendix legend) but separate results and discussion sections. Please either add one more main figure to turn this into an article, or combine the results and discussion sections to publish it as a short report. You can find more information about our article types in our guide to authors online.

We have now 6 Figures to keep our manuscript as an article.

2. Please correct the conflict of interest subheading to "DISCLOSURE AND COMPETING INTERESTS STATEMENT"

We have now corrected the subheading (disclosure and competing interests Statement), line 762.

3. Please remove the author credits from the ms file. We now use CRediT to specify the contributions of each author in the journal submission system. CRediT replaces the author contribution section. Please use the free text box to provide more detailed descriptions, if you wish. See also guide to authors https://www.embopress.org/page/journal/14693178/authorguide#authorshipguidelines.
We have now removed the author credits from ms file and specified the contributions of each author in the journal submission system.

4. Some funding information is missing in our online manuscript handling system, please add.
We have now added the funding information in the online manuscript handling system.

5. The Supplementary Tables 1-5 need to be renamed Datasets EV1-EV5 (Changed in text) and called out accordingly; the legends should be removed from the manuscript text (legend for Table S5 is missing – table S5 is related to glioblastoma data – to exclude from the submission) and inserted in a separate sheet of each Excel file.
Supplementary Table 5 is not called out, please add this callout (table 5 is no longer art of the doc).
We have now renamed the Datasets EV1-EV5 and added the legends in a separate sheet of each Excel file. We removed the old version of table S5 related to the glioblastoma data.

6. The page numbers are missing from the table of content of the APPENDIX file, please add. The figures in the file and table of content should be renamed to Appendix Figure S1-S3; Appendix Supplementary Tables 1-5 should be removed from the table of content.
We have now added the page numbers from the table of content of the APPENDIX file. The figures in the file and table of content have been renamed.

7. I attach to this email a related ms file with comments by our data editors. Please address all comments in the final ms.

We have now performed all the changes and addressed all the comments in the final manuscript.

8. I would like to suggest some minor changes to the title and abstract. Please let me know whether you agree with the following (and please clarify my questions):
Endothelial-derived oxysterols inhibit myeloid-derived suppressor cell expansion at the blood-brain barrier
or
Endothelial cell-derived oxysterol ablation attenuates experimental autoimmune encephalomyelitis

We have changed the title for "Endothelial cell-derived oxysterol ablation attenuates experimental autoimmune encephalomyelitis". Thank you for the suggestions.

We have modified the abstract accordingly to the questions in the text:

"The vasculature is a key regulator of leukocyte trafficking into the central nervous system (CNS) during inflammatory diseases including multiple sclerosis (MS). However, the impact of endothelial-derived

factors on other aspects of CNS immune responses remains unknown. Bioactive lipids, in particular oxysterols downstream of the enzyme Cholesterol-25-hydroxylase (Ch25h), promote neuroinflammation. Nevertheless their CNS functions are largely unravelled. Using a floxed-reporter Ch25h knock-in mice, we traced Ch25h expression to CNS endothelial cells (ECs) and myeloid cells and demonstrated that Ch25h-specific ablation in ECs attenuates experimental autoimmune encephalomyelitis (EAE). Mechanistically, inflamed Ch25h-deficient CNS ECs displayed altered lipid metabolism favoring polymorphonuclear myeloid-derived suppressor cells (PMN-MDSC) expansion that suppresses encephalitogenic T lymphocyte proliferation. Additionally, endothelial Ch25h-deficiency combined with immature neutrophils mobilization into the blood circulation resulted in nearly complete EAE protection. Our findings reveal a central role for CNS endothelial Ch25h in promoting neuroinflammation by regulating the expansion of immunosuppressive myeloid cell populations."

Prof. Caroline Pot
Lausanne University Hospital
Switzerland

Dear Prof. Pot,

I am very pleased to accept your manuscript for publication in the next available issue of EMBO reports. Thank you for your contribution to our journal.

At the end of this email I include important information about how to proceed. Please ensure that you take the time to read the information and complete and return the necessary forms to allow us to publish your manuscript as quickly as possible.

As part of the EMBO publication's Transparent Editorial Process, EMBO reports publishes online a Review Process File to accompany accepted manuscripts. As you are aware, this File will be published in conjunction with your paper and will include the referee reports, your point-by-point response and all pertinent correspondence relating to the manuscript.

If you do NOT want this File to be published, please inform the editorial office within 2 days, if you have not done so already, otherwise the File will be published by default [contact: emboreports@embo.org]. If you do opt out, the Review Process File link will point to the following statement: "No Review Process File is available with this article, as the authors have chosen not to make the review process public in this case." Please note that the author checklist will still be published even if you opt out of the transparent process.

Thank you again for your contribution to EMBO reports and congratulations on a successful publication. Please consider us again in the future for your most exciting work.

Yours sincerely,

Esther Schnapp, PhD
Senior Editor
EMBO reports

****

THINGS TO DO NOW:

Please note that you will be contacted by Wiley Author Services to complete licensing and payment information. The required 'Page Charges Authorization Form' is available here: https://www.embopress.org/pb-assets/embo-site/er_apc.pdf - please download and complete the form and return to embopressproduction@wiley.com

EMBO Press participates in many Publish and Read agreements that allow authors to publish Open Access with reduced/no publication charges. Check your eligibility: https://authorservices.wiley.com/author-resources/Journal-Authors/open-access/affiliation-policies-payments/index.html

You will receive proofs by e-mail approximately 2-3 weeks after all relevant files have been sent to our Production Office; you should return your corrections within 2 days of receiving the proofs.

Please inform us if there is likely to be any difficulty in reaching you at the above address at that time. Failure to meet our deadlines may result in a delay of publication, or publication without your corrections.

All further communications concerning your paper should quote reference number EMBOR-2022-55328V3 and be addressed to emboreports@wiley.com.

Should you be planning a Press Release on your article, please get in contact with emboreports@wiley.com as early as possible, in order to coordinate publication and release dates.

EMBO Press Author Checklist

Corresponding Author Name: Caroline Pot	
Journal Submitted to: EMBO Reports	
Manuscript Number: EMBOR-2022-55328V3	

USEFUL LINKS FOR COMPLETING THIS FORM
The EMBO Journal - Author Guidelines
EMBO Reports - Author Guidelines
Molecular Systems Biology - Author Guidelines
EMBO Molecular Medicine - Author Guidelines

Reporting Checklist for Life Science Articles (updated January 2022)
This checklist is adapted from Materials Design Analysis Reporting (MDAR) Checklist for Authors. MDAR establishes a minimum set of requirements in transparent reporting in the life sciences (see Statement of Task: 10.31222/osf.io/9sm4x). Please follow the journal's guidelines in preparing your manuscript.
Please note that a copy of this checklist will be published alongside your article.

Abridged guidelines for figures
1. Data
The data shown in figures should satisfy the following conditions:
- the data were obtained and processed according to the field's best practice and are presented to reflect the results of the experiments in an accurate and unbiased manner.
- ideally, figure panels should include only measurements that are directly comparable to each other and obtained with the same assay.
- plots include clearly labeled error bars for independent experiments and sample sizes. Unless justified, error bars should not be shown for technical replicates.
- if n<5, the individual data points from each experiment should be plotted. Any statistical test employed should be justified.
- Source Data should be included to report the data underlying figures according to the guidelines set out in the authorship guidelines on Data Presentation.

2. Captions
Each figure caption should contain the following information, for each panel where they are relevant:
- a specification of the experimental system investigated (eg cell line, species name).
- the assay(s) and method(s) used to carry out the reported observations and measurements.
- an explicit mention of the biological and chemical entity(ies) that are being measured.
- an explicit mention of the biological and chemical entity(ies) that are altered/varied/perturbed in a controlled manner.
- the exact sample size (n) for each experimental group/condition, given as a number, not a range;
- a description of the sample collection allowing the reader to understand whether the samples represent technical or biological replicates (including how many animals, litters, cultures, etc.).
- a statement of how many times the experiment shown was independently replicated in the laboratory.
- definitions of statistical methods and measures:
  - common tests, such as t-test (please specify whether paired vs. unpaired), simple χ2 tests, Wilcoxon and Mann-Whitney tests, can be unambiguously identified by name only, but more complex techniques should be described in the methods section;
  - are tests one-sided or two-sided?
  - are there adjustments for multiple comparisons?
  - exact statistical test results, e.g., P values = x but not P values < x;
  - definition of 'center values' as median or average;
  - definition of error bars as s.d. or s.e.m.

Please complete ALL of the questions below.
Select "Not Applicable" only when the requested information is not relevant for your study.

Materials

Newly Created Materials	Information included in the manuscript?	In which section is the information available? (Reagents and Tools Table, Materials and Methods, Figures, Data Availability Section)
New materials and reagents need to be available; do any restrictions apply?	Not Applicable	N.A.

Antibodies	Information included in the manuscript?	In which section is the information available? (Reagents and Tools Table, Materials and Methods, Figures, Data Availability Section)
For **antibodies** provide the following information:<br>- Commercial antibodies: RRID (if possible) or supplier name, catalogue number and or/clone number<br>- Non-commercial: RRID or citation	Yes	Materials and methods

DNA and RNA sequences	Information included in the manuscript?	In which section is the information available? (Reagents and Tools Table, Materials and Methods, Figures, Data Availability Section)
**Short novel DNA or RNA including primers, probes**: provide the sequences.	Yes	Materials and methods

Cell materials	Information included in the manuscript?	In which section is the information available? (Reagents and Tools Table, Materials and Methods, Figures, Data Availability Section)
**Cell lines:** Provide species information, strain. Provide accession number in repository **OR** supplier name, catalog number, clone number, and/**OR** RRID.	Not Applicable	N.A.
**Primary cultures:** Provide species, strain, sex of origin, genetic modification status.	Yes	Materials and methods
Report if the cell lines were recently **authenticated** (e.g., by STR profiling) and tested for mycoplasma contamination.	Not Applicable	N.A.

Experimental animals	Information included in the manuscript?	In which section is the information available? (Reagents and Tools Table, Materials and Methods, Figures, Data Availability Section)
**Laboratory animals or Model organisms:** Provide species, strain, sex, age, genetic modification status. Provide accession number in repository **OR** supplier name, catalog number, clone number, **OR** RRID.	Yes	Materials and methods
**Animal observed in or captured from the field:** Provide species, sex, and age where possible.	Not Applicable	N.A.
Please detail **housing and husbandry conditions**.	Yes	Materials and methods

Plants and microbes	Information included in the manuscript?	In which section is the information available? (Reagents and Tools Table, Materials and Methods, Figures, Data Availability Section)
**Plants:** provide species and strain, ecotype and cultivar where relevant, unique accession number if available, and source (including location for collected wild specimens).	Not Applicable	N.A.
**Microbes:** provide species and strain, unique accession number if available, and source.	Not Applicable	N.A.

Human research participants	Information included in the manuscript?	In which section is the information available? (Reagents and Tools Table, Materials and Methods, Figures, Data Availability Section)
If collected and within the bounds of privacy constraints report on age, sex and gender or ethnicity for all study participants.	Not Applicable	N.A.

Core facilities	Information included in the manuscript?	In which section is the information available? (Reagents and Tools Table, Materials and Methods, Figures, Data Availability Section)
If your work benefited from core facilities, was their service mentioned in the acknowledgments section?	Yes	Acknowledgments

Design

Study protocol	Information included in the manuscript?	In which section is the information available? (Reagents and Tools Table, Materials and Methods, Figures, Data Availability Section)
If study protocol has been **pre-registered, provide DOI in the manuscript**. For clinical trials, provide the trial registration number **OR** cite DOI.	Not Applicable	N.A.
Report the **clinical trial registration number** (at ClinicalTrials.gov or equivalent), where applicable.	Not Applicable	N.A.

Laboratory protocol	Information included in the manuscript?	In which section is the information available? (Reagents and Tools Table, Materials and Methods, Figures, Data Availability Section)
Provide DOI OR other citation details if **external detailed step-by-step protocols** are available.	Yes	Material and methods and References

Experimental study design and statistics	Information included in the manuscript?	In which section is the information available? (Reagents and Tools Table, Materials and Methods, Figures, Data Availability Section)
Include a statement about **sample size** estimate even if no statistical methods were used.	Yes	Materials and methods
Were any steps taken to minimize the effects of subjective bias when allocating animals/samples to treatment (e.g. **randomization procedure**)? If yes, have they been described?	Not Applicable	N.A.
Include a statement about **blinding** even if no blinding was done.	Yes	Materials and methods
Describe **inclusion/exclusion criteria** if samples or animals were excluded from the analysis. Were the criteria pre-established?<br><br>If sample or data points were omitted from analysis, report if this was due to attrition or intentional exclusion and provide justification.	Not Applicable	N.A.
For every figure, are **statistical tests** justified as appropriate? Do the data meet the assumptions of the tests (e.g., normal distribution)? Describe any methods used to assess it. Is there an estimate of variation within each group of data? Is the variance similar between the groups that are being statistically compared?	Yes	In the figure legends and Statistical analysis

Sample definition and in-laboratory replication	Information included in the manuscript?	In which section is the information available? (Reagents and Tools Table, Materials and Methods, Figures, Data Availability Section)
In the figure legends: state number of times the experiment was **replicated** in laboratory.	Yes	In the figures legends
In the figure legends: define whether data describe **technical or biological replicates**.	Yes	In the figures legends

Ethics

Ethics	Information included in the manuscript?	In which section is the information available? (Reagents and Tools Table, Materials and Methods, Figures, Data Availability Section)
Studies involving **human participants**: State details of **authority granting ethics approval** (IRB or equivalent committee(s), provide reference number for approval.	Not Applicable	N.A.
Studies involving **human participants**: Include a statement confirming that **informed consent** was obtained from all subjects and that the experiments conformed to the principles set out in the WMA Declaration of Helsinki and the Department of Health and Human Services Belmont Report.	Not Applicable	N.A.
Studies involving **human participants:** For publication of **patient photos**, include a statement confirming that consent to publish was obtained.	Not Applicable	N.A.
Studies involving experimental **animals**: State details of **authority granting ethics approval** (IRB or equivalent committee(s), provide reference number for approval. Include a statement of compliance with ethical regulations.	Yes	Ethics approval
Studies involving **specimen and field samples:** State if relevant **permits** obtained, provide details of authority approving study; if none were required, explain why.	Not Applicable	N.A.

Dual Use Research of Concern (DURC)	Information included in the manuscript?	In which section is the information available? (Reagents and Tools Table, Materials and Methods, Figures, Data Availability Section)
Could your study fall under dual use research restrictions? Please check biosecurity documents and list of **select agents and toxins** (CDC): https://www.selectagents.gov/sat/list.htm	Not Applicable	N.A.
If you used a select agent, is the security level of the lab appropriate and reported in the manuscript?	Not Applicable	N.A.
If a study is subject to dual use research of concern regulations, is the name of the **authority granting approval and reference number** for the regulatory approval provided in the manuscript?	Not Applicable	N.A.

Reporting

The MDAR framework recommends adoption of discipline-specific guidelines, established and endorsed through community initiatives. Journals have their own policy about requiring specific guidelines and recommendations to complement MDAR.

Adherence to community standards	Information included in the manuscript?	In which section is the information available? (Reagents and Tools Table, Materials and Methods, Figures, Data Availability Section)
State if relevant guidelines or checklists (e.g., **ICMJE, MIBBI, ARRIVE, PRISMA**) have been followed or provided.	Not Applicable	N.A.
For **tumor marker prognostic studies**, we recommend that you follow the **REMARK** reporting guidelines (see link list at top right). See author guidelines, under 'Reporting Guidelines'. Please confirm you have followed these guidelines.	Not Applicable	N.A.
For **phase II and III randomized controlled trials**, please refer to the **CONSORT** flow diagram (see link list at top right) and submit the CONSORT checklist (see link list at top right) with your submission. See author guidelines, under 'Reporting Guidelines'. Please confirm you have submitted this list.	Not Applicable	N.A.

Data Availability

Data availability	Information included in the manuscript?	In which section is the information available? (Reagents and Tools Table, Materials and Methods, Figures, Data Availability Section)
Have **primary datasets** been deposited according to the journal's guidelines (see 'Data Deposition' section) and the respective accession numbers provided in the Data Availability Section?	Yes	Data Availability,
Were **human clinical and genomic datasets** deposited in a public access-controlled repository in accordance to ethical obligations to the patients and to the applicable consent agreement?	Not Applicable	N.A.
Are **computational models** that are central and integral to a study available without restrictions in a machine-readable form? Were the relevant accession numbers or links provided?	Not Applicable	N.A.
If publicly available data were reused, provide the respective **data citations in the reference list**.	Not Applicable	N.A.
